# Supplementary material for: Unraveling the Neuroprotective Effect of Natural Bioactive Compounds Involved in the Modulation of Ischemic Stroke by Network Pharmacology
Source: Pharmaceuticals (Basel). 2023 Sep 28;16(10):1376. doi: 10.3390/ph16101376 (PMC10609914; doi:10.3390/ph16101376)
Supplement: Supplementary file 1 [file pharmaceuticals-16-01376-s001.zip › pharmaceuticals-2596682-supplementary.pdf]

	Disease	Class
1	Sroke	Flavonoids
1	Sroke	Flavonoids
1	Sroke	Flavonoids
1	Sroke	Flavonoids
2	Sroke	Flavonoids
3	Sroke	Flavonoids
3	Sroke	Flavonoids
3	Sroke	Flavonoids
3	Sroke	Flavonoids
3	Sroke	Flavonoids
3	Sroke	Flavonoids
3	Sroke	Flavonoids
3	Sroke	Flavonoids
4	Sroke	Flavonoids
4	Sroke	Flavonoids
4	Sroke	Flavonoids
5	Sroke	Flavonoids
5	Sroke	Flavonoids
5	Sroke	Flavonoids
5	Sroke	Flavonoids
6	Sroke	Flavonoids
6	Sroke	Flavonoids
6	Sroke	Flavonoids
6	Sroke	Flavonoids
6	Sroke	Flavonoids
6	Sroke	Flavonoids
6	Sroke	Flavonoids
6	Sroke	Flavonoids
6	Sroke	Flavonoids
7	Sroke	Flavonoids
7	Sroke	Flavonoids
7	Sroke	Flavonoids
7	Sroke	Flavonoids
8	Sroke	Flavonoids
8	Sroke	Flavonoids
8	Sroke	Flavonoids
8	Sroke	Flavonoids
8	Sroke	Flavonoids
9	Sroke	Flavonoids
9	Sroke	Flavonoids
9	Sroke	Flavonoids
9	Sroke	Flavonoids
9	Sroke	Flavonoids
9	Sroke	Flavonoids
9	Sroke	Flavonoids
9	Sroke	Flavonoids

10	Sroke	Flavonoids
10	Sroke	Flavonoids
10	Sroke	Flavonoids
10	Sroke	Flavonoids
10	Sroke	Flavonoids
11	Sroke	Flavonoids
11	Sroke	Flavonoids
11	Sroke	Flavonoids
12	Sroke	Flavonoids
12	Sroke	Flavonoids
12	Sroke	Flavonoids
12	Sroke	Flavonoids
12	Sroke	Flavonoids
12	Sroke	Flavonoids
12	Sroke	Flavonoids
12	Sroke	Flavonoids
12	Sroke	Flavonoids
12	Sroke	Flavonoids
12	Sroke	Flavonoids
13	Sroke	Flavonoids
13	Sroke	Flavonoids
13	Sroke	Flavonoids
13	Sroke	Flavonoids
13	Sroke	Flavonoids
14	Sroke	Flavonoids
14	Sroke	Flavonoids
14	Sroke	Flavonoids
14	Sroke	Flavonoids
14	Sroke	Flavonoids
15	Sroke	Flavonoids
15	Sroke	Flavonoids
15	Sroke	Flavonoids
16	Sroke	Flavonoids
16	Sroke	Flavonoids
16	Sroke	Flavonoids
16	Sroke	Flavonoids
17	Sroke	Flavonoids
17	Sroke	Flavonoids
17	Sroke	Flavonoids
17	Sroke	Flavonoids
17	Sroke	Flavonoids
17	Sroke	Flavonoids
17	Sroke	Flavonoids
17	Sroke	Flavonoids
17	Sroke	Flavonoids
17	Sroke	Flavonoids

17	Sroke	Flavonoids
17	Sroke	Flavonoids
17	Sroke	Flavonoids
17	Sroke	Flavonoids
18	Sroke	Flavonoids
18	Sroke	Flavonoids
18	Sroke	Flavonoids
18	Sroke	Flavonoids
18	Sroke	Flavonoids
18	Sroke	Flavonoids
18	Sroke	Flavonoids
18	Sroke	Flavonoids
18	Sroke	Flavonoids
18	Sroke	Flavonoids
19	Sroke	Flavonoids
19	Sroke	Flavonoids
19	Sroke	Flavonoids
20	Sroke	Flavonoids
20	Sroke	Flavonoids
20	Sroke	Flavonoids
20	Sroke	Flavonoids
20	Sroke	Flavonoids
21	Sroke	Flavonoids
21	Sroke	Flavonoids
22	Sroke	Flavonoids
22	Sroke	Flavonoids
23	Sroke	Flavonoids
23	Sroke	Flavonoids
23	Sroke	Flavonoids
23	Sroke	Flavonoids
23	Sroke	Flavonoids
24	Sroke	Flavonoids
25	Sroke	Flavonoids
25	Sroke	Flavonoids
25	Sroke	Flavonoids
25	Sroke	Flavonoids
26	Sroke	Flavonoids
26	Sroke	Flavonoids
26	Sroke	Flavonoids
26	Sroke	Flavonoids
26	Sroke	Flavonoids
26	Sroke	Flavonoids
27	Sroke	Flavonoids
27	Sroke	Flavonoids
28	Sroke	Alkaloids
28	Sroke	Alkaloids

28	Sroke	Alkaloids
29	Sroke	Alkaloids
29	Sroke	Alkaloids
29	Sroke	Alkaloids
29	Sroke	Alkaloids
30	Sroke	Alkaloids
30	Sroke	Alkaloids
30	Sroke	Alkaloids
31	Sroke	Alkaloids
31	Sroke	Alkaloids
32	Sroke	Alkaloids
32	Sroke	Alkaloids
32	Sroke	Alkaloids
32	Sroke	Alkaloids
32	Sroke	Alkaloids
33	Sroke	Alkaloids
33	Sroke	Alkaloids
33	Sroke	Alkaloids
33	Sroke	Alkaloids
34	Sroke	Alkaloids
34	Sroke	Alkaloids
34	Sroke	Alkaloids
34	Sroke	Alkaloids
35	Sroke	Alkaloids
35	Sroke	Alkaloids
35	Sroke	Alkaloids
36	Sroke	Alkaloids
37	Sroke	Polyphenols
37	Sroke	Polyphenols
37	Sroke	Polyphenols
37	Sroke	Polyphenols
37	Sroke	Polyphenols
38	Sroke	Polyphenols
38	Sroke	Polyphenols
38	Sroke	Polyphenols
38	Sroke	Polyphenols
38	Sroke	Polyphenols
39	Sroke	Polyphenols
39	Sroke	Polyphenols
39	Sroke	Polyphenols
40	Sroke	Polyphenols
40	Sroke	Polyphenols
40	Sroke	Polyphenols
40	Sroke	Polyphenols
41	Sroke	Polyphenols
41	Sroke	Polyphenols

42	Sroke	Polyphenols
43	Sroke	Polyphenols
43	Sroke	Polyphenols
43	Sroke	Polyphenols
43	Sroke	Polyphenols
44	Sroke	Polyphenols
44	Sroke	Polyphenols
44	Sroke	Polyphenols
44	Sroke	Polyphenols
44	Sroke	Polyphenols
45	Sroke	Polyphenols
45	Sroke	Polyphenols
45	Sroke	Polyphenols
46	Sroke	Polyphenols
46	Sroke	Polyphenols
46	Sroke	Polyphenols
46	Sroke	Polyphenols
46	Sroke	Polyphenols
47	Sroke	Glycosides
47	Sroke	Glycosides
47	Sroke	Glycosides
47	Sroke	Glycosides
47	Sroke	Glycosides
47	Sroke	Glycosides
48	Sroke	Glycosides
48	Sroke	Glycosides
48	Sroke	Glycosides
49	Sroke	Glycosides
49	Sroke	Glycosides
49	Sroke	Glycosides
49	Sroke	Glycosides
49	Sroke	Glycosides
49	Sroke	Glycosides
49	Sroke	Glycosides
49	Sroke	Glycosides
49	Sroke	Glycosides
49	Sroke	Glycosides
49	Sroke	Glycosides
49	Sroke	Glycosides
50	Sroke	Glycosides
50	Sroke	Glycosides
50	Sroke	Glycosides
50	Sroke	Glycosides
51	Sroke	Glycosides
51	Sroke	Glycosides
51	Sroke	Glycosides

51	Sroke	Glycosides
52	Sroke	Iridoid glycoside
52	Sroke	Iridoid glycoside
52	Sroke	Iridoid glycoside
52	Sroke	Iridoid glycoside
52	Sroke	Iridoid glycoside
53	Sroke	Iridoid glycoside
54	Sroke	Iridoid glycoside
55	Sroke	Iridoid glycoside
55	Sroke	Iridoid glycoside
55	Sroke	Iridoid glycoside
56	Sroke	Iridoid glycoside
56	Sroke	Iridoid glycoside
56	Sroke	Iridoid glycoside
57	Sroke	Iridoid glycoside
58	Sroke	Iridoid glycoside
58	Sroke	Iridoid glycoside
58	Sroke	Iridoid glycoside
58	Sroke	Iridoid glycoside
58	Sroke	Iridoid glycoside
58	Sroke	Iridoid glycoside
58	Sroke	Iridoid glycoside
58	Sroke	Iridoid glycoside
58	Sroke	Iridoid glycoside
58	Sroke	Iridoid glycoside
59	Sroke	Iridoid glycoside
59	Sroke	Iridoid glycoside
59	Sroke	Iridoid glycoside
59	Sroke	Iridoid glycoside
60	Sroke	Saponin
60	Sroke	Saponin
60	Sroke	Saponin
61	Sroke	Saponin
62	Sroke	Saponin
62	Sroke	Saponin
62	Sroke	Saponin
63	Sroke	Saponin
63	Sroke	Saponin
64	Sroke	Saponin
65	Sroke	Saponin
65	Sroke	Saponin
65	Sroke	Saponin
65	Sroke	Saponin
65	Sroke	Saponin
65	Sroke	Saponin
65	Sroke	Saponin

65	Sroke	Saponin
65	Sroke	Saponin
66	Sroke	Saponin
66	Sroke	Saponin
66	Sroke	Saponin
66	Sroke	Saponin
66	Sroke	Saponin
66	Sroke	Saponin
67	Sroke	Saponin
67	Sroke	Saponin
67	Sroke	Saponin
67	Sroke	Saponin
68	Sroke	Terpenoids
68	Sroke	Terpenoids
69	Sroke	Terpenoids
70	Sroke	Terpenoids
71	Sroke	Terpenoids
71	Sroke	Terpenoids
71	Sroke	Terpenoids
71	Sroke	Terpenoids
71	Sroke	Terpenoids
72	Sroke	Terpenoids
72	Sroke	Terpenoids
72	Sroke	Terpenoids
72	Sroke	Terpenoids
73	Sroke	Terpenoids
73	Sroke	Terpenoids
73	Sroke	Terpenoids
73	Sroke	Terpenoids
73	Sroke	Terpenoids
73	Sroke	Terpenoids
73	Sroke	Terpenoids
73	Sroke	Terpenoids
74	Sroke	Terpenoids
74	Sroke	Terpenoids
74	Sroke	Terpenoids
75	Sroke	Terpenoids
75	Sroke	Terpenoids
75	Sroke	Terpenoids
75	Sroke	Terpenoids
76	Sroke	Terpenoids
76	Sroke	Terpenoids
76	Sroke	Terpenoids
77	Sroke	Terpenoids
77	Sroke	Terpenoids
77	Sroke	Terpenoids

77	Sroke	Terpenoids
78	Sroke	Terpenoids
78	Sroke	Terpenoids
78	Sroke	Terpenoids
78	Sroke	Terpenoids
78	Sroke	Terpenoids
78	Sroke	Terpenoids
78	Sroke	Terpenoids
79	Sroke	Terpenoids
79	Sroke	Terpenoids
79	Sroke	Terpenoids
79	Sroke	Terpenoids
79	Sroke	Terpenoids
79	Sroke	Terpenoids
79	Sroke	Terpenoids
79	Sroke	Terpenoids
80	Sroke	Terpenoids
80	Sroke	Terpenoids
80	Sroke	Terpenoids
80	Sroke	Terpenoids
81	Sroke	Antraquinone
81	Sroke	Antraquinone
81	Sroke	Antraquinone
82	Sroke	Antraquinone
82	Sroke	Antraquinone
82	Sroke	Antraquinone
82	Sroke	Antraquinone
83	Sroke	Adenosine analog
83	Sroke	Adenosine analog
83	Sroke	Adenosine analog
83	Sroke	Adenosine analog
83	Sroke	Adenosine analog
84	Sroke	Adenosine analog
85	Sroke	Adenosine analog
85	Sroke	Adenosine analog
85	Sroke	Adenosine analog
85	Sroke	Adenosine analog
85	Sroke	Adenosine analog
85	Sroke	Adenosine analog
86	Sroke	N-linked glycans
86	Sroke	N-linked glycans
86	Sroke	N-linked glycans
86	Sroke	N-linked glycans
86	Sroke	N-linked glycans
86	Sroke	N-linked glycans
86	Sroke	N-linked glycans

86	Sroke	N-linked glycans
86	Sroke	N-linked glycans
86	Sroke	N-linked glycans
87	Sroke	N-linked glycans
87	Sroke	N-linked glycans
87	Sroke	N-linked glycans
87	Sroke	N-linked glycans
87	Sroke	N-linked glycans
88	Sroke	N-linked glycans
88	Sroke	N-linked glycans
88	Sroke	N-linked glycans
88	Sroke	N-linked glycans
88	Sroke	N-linked glycans
91	Sroke	Phytocannabinoids
91	Sroke	Phytocannabinoids
92	Sroke	Phytocannabinoids
93	Sroke	Phytocannabinoids
94	Sroke	Phytocannabinoids
94	Sroke	Phytocannabinoids
94	Sroke	Phytocannabinoids
94	Sroke	Phytocannabinoids
95	Sroke	Trans-cinnamaldehyde
95	Sroke	Trans-cinnamaldehyde
95	Sroke	Trans-cinnamaldehyde
95	Sroke	Trans-cinnamaldehyde
95	Sroke	Trans-cinnamaldehyde
95	Sroke	Trans-cinnamaldehyde
95	Sroke	Trans-cinnamaldehyde
95	Sroke	Trans-cinnamaldehyde
95	Sroke	Trans-cinnamaldehyde
95	Sroke	Trans-cinnamaldehyde

Chemical compound
Baicalin
Baicalin
Baicalin
Baicalin
Baicalin
Baicalin
Baicalin
Baicalin
Baicalin
Baicalin
Baicalin
Baicalin
Baicalin
Baicalin
Baicalin
Baicalin
Baicalin
Baicalin
Baicalin
Baicalin
Baicalin
Baicalin
Baicalin
Baicalin
Baicalin
Baicalin
Baicalin
Baicalin
Baicalin
Scutellarin
Scutellarin
Scutellarin
Scutellarin
Scutellarin
Scutellarin
Scutellarin
Scutellarin
Scutellarin
Scutellarin
Scutellarin
Scutellarin
Scutellarin
Scutellarin
Scutellarin
Scutellarin
Scutellarin

Scutellarin
Scutellarin
Scutellarin
Scutellarin
Scutellarin
Vitexin
Vitexin
Vitexin
Vitexin
Vitexin
Vitexin
Vitexin
Vitexin
Vitexin
Vitexin
Vitexin
Vitexin
Vitexin
Vitexin
Apigenin
Apigenin
Apigenin
Apigenin
Apigenin
Apigenin
Apigenin
Apigenin
Apigenin
Apigenin
Icariin
Icariin
Icariin
Icariin
Icariin
Icariin
Icariin
Icariin
Icariin
Icariin
Icariin
Icariin
Icariin
Icariin
Icariin
Icariin
Icariin

Icariin
Icariin
Icariin
Icariin
Icariin
Icariin
Icariin
Icariin
Icariin
Icariin
Icariin
Icariin
Icariin
Icariin
Quercetin
Quercetin
Quercetin
Quercetin
Quercetin
Quercetin
Quercetin
Quercetin
Calycosin
Calycosin
Calycosin
Calycosin
Calycosin
Calycosin
Calycosin
Calycosin
Calycosin
Xanthohumol
Xanthohumol
Xanthohumol
Xanthohumol
Xanthohumol
Carthamin yellow
Carthamin yellow
Carthamin yellow
Carthamin yellow
Carthamin yellow
Carthamin yellow
Dihydromyricetin
Dihydromyricetin
Berberine
Berberine

Berberine
Berberine
Berberine
Berberine
Berberine
Ligustrazine/tetramethylpyrazine
Ligustrazine/tetramethylpyrazine
Ligustrazine/tetramethylpyrazine
Ligustrazine/tetramethylpyrazine
Ligustrazine/tetramethylpyrazine
Ligustrazine/tetramethylpyrazine
Ligustrazine/tetramethylpyrazine
Ligustrazine/tetramethylpyrazine
Ligustrazine/tetramethylpyrazine
Ligustrazine/tetramethylpyrazine
Daurisoline
Daurisoline
Daurisoline
Daurisoline
Tetrahydropalmatine
Tetrahydropalmatine
Tetrahydropalmatine
Tetrahydropalmatine
Tetrahydropalmatine
Tetrahydropalmatine
Tetrahydropalmatine
Neferine
Curcumin
Curcumin
Curcumin
Curcumin
Curcumin
Curcumin
Curcumin
Curcumin
Curcumin
Curcumin
Curcumin
Curcumin
Curcumin
Curcumin
Curcumin
Curcumin
Curcumin
Curcumin
Curcumin

Curcumin
Curcumin
Curcumin
Curcumin
Curcumin
Resveratrol
Resveratrol
Resveratrol
Resveratrol
Resveratrol
Resveratrol
Resveratrol
Resveratrol
Resveratrol
Resveratrol
Resveratrol
Resveratrol
Resveratrol
Asiaticoside
Asiaticoside
Asiaticoside
Asiaticoside
Asiaticoside
Asiaticoside
Asiaticoside
Asiaticoside
Asiaticoside
Salidroside
Salidroside
Salidroside
Salidroside
Salidroside
Salidroside
Salidroside
Salidroside
Salidroside
Salidroside
Salidroside
Salidroside
Salidroside
Salidroside
Salidroside
Salidroside
Salidroside
Salidroside
Salidroside

Salidroside
Geniposide
Geniposide
Geniposide
Geniposide
Geniposide
Geniposide
Geniposide
Catalpol
Catalpol
Catalpol
Picroside II
Picroside II
Picroside II
Picroside II
Picroside II
Picroside II
Picroside II
Picroside II
Picroside II
Picroside II
Picroside II
Picroside II
Picroside II
Picroside II
Picroside II
Picroside II
Picroside II
Picroside II
Ginsenoside Rg1
Ginsenoside Rg1
Ginsenoside Rg1
Ginsenoside Rg1
Ginsenoside Rg1
Ginsenoside Rg1
Ginsenoside Rg1
Astragaloside IV
Astragaloside IV
Astragaloside IV
Astragaloside IV
Astragaloside IV
Astragaloside IV
Astragaloside IV
Astragaloside IV
Astragaloside IV
Astragaloside IV

Astragaloside IV
Astragaloside IV
Astragaloside IV
Astragaloside IV
Astragaloside IV
Astragaloside IV
Astragaloside IV
Astragaloside IV
Panax notoginseng saponin (TSPN)
Panax notoginseng saponin (TSPN)
Panax notoginseng saponin (TSPN)
Panax notoginseng saponin (TSPN)
Andrographolide
Andrographolide
Andrographolide
Andrographolide
Andrographolide
Andrographolide
Andrographolide
Andrographolide
Andrographolide
Ginkgolide B
Ginkgolide B
Ginkgolide B
Ginkgolide B
Ginkgolide A (GA), ginkgolide B (GB), ginkgolide K (GK) and bilobalide (BB)
Ginkgolide A (GA), ginkgolide B (GB), ginkgolide K (GK) and bilobalide (BB)
Ginkgolide B (GB)
Ginkgolide B (GB)
Ginkgolide B (GB)
Ginkgolide B (GB)
Ginkgolide B (GB)
Ginkgolide B (GB)
Ginkgolides A, B and C
Ginkgolides A, B and C
Ginkgolides A, B and C
Ginkgolides A (GA), Ginkgolides B (GB), and Ginkgolides K (GK)
Ginkgolides A (GA), Ginkgolides B (GB), and Ginkgolides K (GK)
Ginkgolides A (GA), Ginkgolides B (GB), and Ginkgolides K (GK)
Ginkgolides A (GA), Ginkgolides B (GB), and Ginkgolides K (GK)
10-O-(N N-dimethylaminoethyl)-ginkgolide B methane-sulfonate (XQ-1H)
10-O-(N N-dimethylaminoethyl)-ginkgolide B methane-sulfonate (XQ-1H)
10-O-(N N-dimethylaminoethyl)-ginkgolide B methane-sulfonate (XQ-1H)
Borneol
Borneol
Borneol

Borneol
Borneol
Borneol
Borneol
Borneol
Borneol
Borneol
Borneol
Borneol
Borneol
Borneol
Borneol
Borneol
Borneol
Borneol
Borneol
Daidzein
Daidzein
Daidzein
Daidzein
Emodin
Emodin
Emodin
Emodin
Emodin
Emodin
Emodin
Cordycepin
Cordycepin
Cordycepin
Cordycepin
Cordycepin
Cordycepin
Cordycepin
Cordycepin
Cordycepin
Cordycepin
Cordycepin
Cordycepin
Fucose
Fucose
Fucose
Fucose
Fucose
Fucose
Fucose

Fucose
Fucose
Fucose
Fucose
Fucose
Fucose
Fucose
Fucose
Fucose
Fucose
Fucose
Fucose
Fucose
Cannabidiol
Cannabidiol
Cannabidiol
Cannabidiol
Cannabidiol
Cannabidiol
Cannabidiol
Cannabidiol
Cinnamaldehyde
Cinnamaldehyde
Cinnamaldehyde
Cinnamaldehyde
Cinnamaldehyde
Cinnamaldehyde
Cinnamaldehyde
Cinnamaldehyde
Cinnamaldehyde
Cinnamaldehyde

Model
Gerbils
Gerbils
Gerbils
Gerbils
Gerbils
Gerbils
Gerbils
Gerbils
Gerbils
Gerbils
Gerbils
Gerbils
Gerbils
Sprague–Dawley (SD) rats
Sprague–Dawley (SD) rats
Sprague–Dawley (SD) rats
Sprague–Dawley (SD) rats
Sprague–Dawley (SD) rats
Sprague–Dawley (SD) rats
Sprague–Dawley (SD) rats
Sprague–Dawley (SD) rats
Sprague–Dawley (SD) rats
Sprague–Dawley (SD) rats
Sprague–Dawley (SD) rats
Sprague–Dawley (SD) rats
Sprague–Dawley (SD) rats
Sprague–Dawley (SD) rats
Sprague–Dawley (SD) rats
Sprague–Dawley (SD) rats
Sprague–Dawley (SD) rats
Sprague–Dawley (SD) rats
Sprague–Dawley (SD) rats
Sprague–Dawley (SD) rats
Sprague–Dawley (SD) rats
Sprague–Dawley (SD) rats
Sprague–Dawley (SD) rats
Sprague–Dawley (SD) rats
Sprague–Dawley (SD) rats
Murine BV-2 microglial cell line
Murine BV-2 microglial cell line
Murine BV-2 microglial cell line
Murine BV-2 microglial cell line
Murine BV-2 microglial cell line
Murine BV-2 microglial cell line
Murine BV-2 microglial cell line
Murine BV-2 microglial cell line

Wistar rats
Wistar rats
Wistar rats
Wistar rats
Wistar rats
Human brain microvascular endothelial cells (HBMECs) cell culture with oxygen and glucose deprivation/reoxy
Human brain microvascular endothelial cells (HBMECs) cell culture with oxygen and glucose deprivation/reoxy
Human brain microvascular endothelial cells (HBMECs) cell culture with oxygen and glucose deprivation/reoxy
Sprague–Dawley (SD) rats
Sprague–Dawley (SD) rats
Sprague–Dawley (SD) rats
Sprague–Dawley (SD) rats
Sprague–Dawley (SD) rats
Sprague–Dawley (SD) rats
Sprague–Dawley (SD) rats
Sprague–Dawley (SD) rats
Sprague–Dawley (SD) rats
Sprague–Dawley (SD) rats
Sprague–Dawley (SD) rats
Human brain microvascular endothelial cells (HBMVECs)
Human brain microvascular endothelial cells (HBMVECs)
Human brain microvascular endothelial cells (HBMVECs)
Sprague–Dawley (SD) rats
Sprague–Dawley (SD) rats
Sprague–Dawley (SD) rats
Sprague–Dawley (SD) rats
Sprague–Dawley (SD) rats
Sprague–Dawley (SD) rats
Sprague–Dawley (SD) rats
Sprague–Dawley (SD) rats
Sprague–Dawley (SD) rats
Sprague–Dawley (SD) rats
Sprague–Dawley (SD) rats
Sprague–Dawley (SD) rats
Sprague–Dawley (SD) rats
Sprague–Dawley (SD) rats
Primary microglia culture
Primary microglia culture
Primary microglia culture
Primary microglia culture
Primary microglia culture
Primary microglia culture
Primary microglia culture
Primary microglia culture
Primary microglia culture
Primary microglia culture

Primary microglia culture
Primary microglia culture
Primary microglia culture
Primary microglia culture
Sprague–Dawley (SD) rats
Sprague–Dawley (SD) rats
Sprague–Dawley (SD) rats
Sprague–Dawley (SD) rats
Sprague–Dawley (SD) rats
Sprague–Dawley (SD) rats
Sprague–Dawley (SD) rats
Sprague–Dawley (SD) rats
Sprague–Dawley (SD) rats
Sprague–Dawley (SD) rats
Sprague–Dawley (SD) rats
Sprague–Dawley (SD) rats
Sprague–Dawley (SD) rats
Sprague–Dawley (SD) rats
Sprague–Dawley (SD) rats
Sprague–Dawley (SD) rats
Sprague–Dawley (SD) rats
Sprague–Dawley (SD) rats
Sprague–Dawley (SD) rats
Sprague–Dawley (SD) rats
Sprague–Dawley (SD) rats
Sprague–Dawley (SD) rats
Sprague–Dawley (SD) rats
Sprague–Dawley (SD) rats
Sprague–Dawley (SD) rats
Sprague–Dawley (SD) rats
Sprague–Dawley (SD) rats
Sprague–Dawley (SD) rats
Sprague–Dawley (SD) rats
Sprague–Dawley (SD) rats
Sprague–Dawley (SD) rats
Sprague–Dawley (SD) rats
Sprague–Dawley (SD) rats
Sprague–Dawley (SD) rats
Sprague–Dawley (SD) rats
Sprague–Dawley (SD) rats
Sprague–Dawley (SD) rats
Sprague–Dawley (SD) rats
Sprague–Dawley (SD) rats
Sprague–Dawley (SD) rats
PC12 oxygen-glucose-deprived (OGD) cells
PC12 oxygen-glucose-deprived (OGD) cells

PC12 oxygen-glucose-deprived (OGD) cells
Wistar rats
Wistar rats
Wistar rats
Wistar rats
Sprague–Dawley (SD) rats
Sprague–Dawley (SD) rats
Sprague–Dawley (SD) rats
Sprague–Dawley (SD) rats
Sprague–Dawley (SD) rats
Wistar rats
Wistar rats
Wistar rats
Wistar rats
Wistar rats
Sprague–Dawley (SD) rats
Sprague–Dawley (SD) rats
Sprague–Dawley (SD) rats
Sprague–Dawley (SD) rats
Sprague–Dawley (SD) rats
Sprague–Dawley (SD) rats
Sprague–Dawley (SD) rats
Sprague–Dawley (SD) rats
C57BL/6N mice
C57BL/6N mice
C57BL/6N mice
PC12 oxygen-glucose-deprived (OGD) cells
Sprague–Dawley (SD) rats
Sprague–Dawley (SD) rats
Sprague–Dawley (SD) rats
Sprague–Dawley (SD) rats
Sprague–Dawley (SD) rats
Sprague–Dawley (SD) rats
Sprague–Dawley (SD) rats
Sprague–Dawley (SD) rats
Sprague–Dawley (SD) rats
Sprague–Dawley (SD) rats
Primary cultures of cortical neurons
Primary cultures of cortical neurons
Primary cultures of cortical neurons
C57BL/6 mice
Mouse N2a cells culture
Mouse N2a cells culture
Mouse N2a cells culture
PC12 oxygen-glucose-deprived (OGD) cells
PC12 oxygen-glucose-deprived (OGD) cells

PC12 oxygen-glucose-deprived (OGD) cells
Sprague–Dawley (SD) rats
Sprague–Dawley (SD) rats
Sprague–Dawley (SD) rats
Sprague–Dawley (SD) rats
Wistar rats
Wistar rats
Wistar rats
Wistar rats
Wistar rats
Sprague–Dawley (SD) rats
Sprague–Dawley (SD) rats
Sprague–Dawley (SD) rats
Sprague–Dawley (SD) rats
Sprague–Dawley (SD) rats
Sprague–Dawley (SD) rats
Sprague–Dawley (SD) rats
Sprague–Dawley (SD) rats
ICR mice
ICR mice
ICR mice
ICR mice
ICR mice
ICR mice
Primary cultured mouse cortical neuron
Primary cultured mouse cortical neuron
Primary cultured mouse cortical neuron
C57BL/6 mice
C57BL/6 mice
C57BL/6 mice
C57BL/6 mice
C57BL/6 mice
C57BL/6 mice
C57BL/6 mice
C57BL/6 mice
C57BL/6 mice
C57BL/6 mice
C57BL/6 mice
C57BL/6 mice
OGD/R model in BV2 cells
OGD/R model in BV2 cells
OGD/R model in BV2 cells
OGD/R model in BV2 cells
Sprague–Dawley (SD) rats
Sprague–Dawley (SD) rats
Sprague–Dawley (SD) rats

Sprague–Dawley (SD) rats
OGD/R model in BV2 cells
OGD/R model in BV2 cells
OGD/R model in BV2 cells
OGD/R model in BV2 cells
OGD/R model in BV2 cells
Sprague–Dawley (SD) rats
Sprague–Dawley (SD) rats
Gerbils
Gerbils
Gerbils
Wistar rats
Wistar rats
Wistar rats
Wistar rats
Wistar rats
Wistar rats
Wistar rats
Wistar rats
Wistar rats
Wistar rats
Wistar rats
Wistar rats
Wistar rats
Wistar rats
Wistar rats
Wistar rats
Wistar rats
Wistar rats
Sprague–Dawley (SD) rats
Sprague–Dawley (SD) rats
Sprague–Dawley (SD) rats
Sprague–Dawley (SD) rats
Sprague–Dawley (SD) rats
Sprague–Dawley (SD) rats
Sprague–Dawley (SD) rats
Sprague–Dawley (SD) rats
Sprague–Dawley (SD) rats
ICR mice
Sprague–Dawley (SD) rats
Sprague–Dawley (SD) rats
Sprague–Dawley (SD) rats
Sprague–Dawley (SD) rats
Sprague–Dawley (SD) rats
Sprague–Dawley (SD) rats
Sprague–Dawley (SD) rats

Sprague–Dawley (SD) rats
Sprague–Dawley (SD) rats
Sprague–Dawley (SD) rats
Sprague–Dawley (SD) rats
Sprague–Dawley (SD) rats
Sprague–Dawley (SD) rats
Sprague–Dawley (SD) rats
Sprague–Dawley (SD) rats
Wistar rats
Wistar rats
Wistar rats
Wistar rats
ICR mice
ICR mice
Cerebral endothelial cells (CECs) cultures
Cerebral endothelial cells (CECs) cultures
Male 6-week-old specific pathogen-free (SPF) Kunming mice
Male 6-week-old specific pathogen-free (SPF) Kunming mice
Male 6-week-old specific pathogen-free (SPF) Kunming mice
Male 6-week-old specific pathogen-free (SPF) Kunming mice
Male 6-week-old specific pathogen-free (SPF) Kunming mice
Wistar rats
Wistar rats
Wistar rats
Wistar rats
SH-SY5Y cells
Sprague–Dawley (SD) rats
Sprague–Dawley (SD) rats
Sprague–Dawley (SD) rats
Sprague–Dawley (SD) rats
Sprague–Dawley (SD) rats
Sprague–Dawley (SD) rats
Sprague–Dawley (SD) rats
PC12 oxygen-glucose-deprived (OGD) cells
PC12 oxygen-glucose-deprived (OGD) cells
PC12 oxygen-glucose-deprived (OGD) cells
Sprague–Dawley (SD) rats
Sprague–Dawley (SD) rats
Sprague–Dawley (SD) rats
Sprague–Dawley (SD) rats
Wistar rats
PC12 oxygen-glucose-deprived (OGD) cells
PC12 oxygen-glucose-deprived (OGD) cells
Primary neuronal cultures of cerebral cortex
Primary neuronal cultures of cerebral cortex
Primary neuronal cultures of cerebral cortex

Primary neuronal cultures of cerebral cortex
Sprague–Dawley (SD) rats
Sprague–Dawley (SD) rats
Sprague–Dawley (SD) rats
Sprague–Dawley (SD) rats
Sprague–Dawley (SD) rats
Sprague–Dawley (SD) rats
Sprague–Dawley (SD) rats
Sprague–Dawley (SD) rats
Sprague–Dawley (SD) rats
Sprague–Dawley (SD) rats
Sprague–Dawley (SD) rats
Sprague–Dawley (SD) rats
Sprague–Dawley (SD) rats
Sprague–Dawley (SD) rats
Sprague–Dawley (SD) rats
ICR rats
ICR rats
ICR rats
ICR rats
HT22 cells
HT22 cells
HT22 cells
PC12 oxygen-glucose-deprived (OGD) cells
PC12 oxygen-glucose-deprived (OGD) cells
PC12 oxygen-glucose-deprived (OGD) cells
PC12 oxygen-glucose-deprived (OGD) cells
Male kunming mice
Male kunming mice
Male kunming mice
Male kunming mice
Male kunming mice
Sprague–Dawley (SD) rats
C57BL/6 mice
C57BL/6 mice
C57BL/6 mice
C57BL/6 mice
C57BL/6 mice
C57BL/6 mice
Sprague–Dawley (SD) rats
Sprague–Dawley (SD) rats
Sprague–Dawley (SD) rats
Sprague–Dawley (SD) rats
Sprague–Dawley (SD) rats
Sprague–Dawley (SD) rats
Sprague–Dawley (SD) rats

Sprague–Dawley (SD) rats
Sprague–Dawley (SD) rats
Sprague–Dawley (SD) rats
Gerbils
Gerbils
Gerbils
Gerbils
Gerbils
Gerbils
Gerbils
Gerbils
Gerbils
Gerbils
Wistar rats
Wistar rats
Wistar rats
Male Swiss mice
C57BL/6 mice
C57BL/6 mice
C57BL/6 mice
C57BL/6 mice
C57BL/6 mice
C57BL/6 mice
C57BL/6 mice
C57BL/6 mice
C57BL/6 mice
C57BL/6 mice
C57BL/6 mice
C57BL/6 mice
C57BL/6 mice
C57BL/6 mice

Study	Pathogeny / associated process
In vivo	Neuronal apoptosis
In vivo	Neuronal apoptosis
In vivo	Neuroplasticity
In vivo	Neuroplasticity
In vivo	Neuronal apoptosis
In vivo	Neurotransmition
In vivo	Neurotransmition
In vivo	Neurotransmition
In vivo	Neurotransmition
In vivo	Neurotransmition
In vivo	Neurotransmition
In vivo	Neurotransmition
In vivo	Neurotransmition
In vivo	Neuroinflamation
In vivo	Neuroinflamation
In vivo	Neuroplasticity
In vivo	Neuronal apoptosis
In vivo	Neuronal apoptosis
In vivo	Neuronal apoptosis
In vivo	Neuronal apoptosis
In vivo	Neuroinflamation
In vivo	Neuroinflamation
In vivo	Neuroinflamation
In vivo	Neuronal apoptosis
In vivo	Neuronal apoptosis
In vivo	Neuronal apoptosis
In vivo	Autophagy dysfunction
In vivo	Autophagy dysfunction
In vivo	Autophagy dysfunction
In vivo	Neuroplasticity
In vivo	Neuroplasticity
In vivo	Neuroplasticity
In vivo	Neuroplasticity
In vivo	Neuroinflamation
In vivo	Neuroinflamation
In vivo	Neuroinflamation
In vivo	Activated microglia
In vivo	Activated microglia
In vitro	Neuroinflamation
In vitro	Neuroinflamation
In vitro	Neuroinflamation
In vitro	Neuroinflamation
In vitro	Neuroinflamation
In vitro	Neuroinflamation
In vitro	Neuroinflamation
In vitro	Neuroinflamation

In vivo	Neurotransmition
In vivo	Neurotransmition
In vivo	Neurotransmition
In vivo	Neurotransmition
In vivo	Neurotransmition
In vitro	Neuronal apoptosis
In vitro	Neuronal apoptosis
In vitro	Oxidative stress
In vivo	Brain infarction
In vivo	Neuronal apoptosis
In vivo	Neuronal apoptosis
In vivo	Neuronal apoptosis
In vivo	Neuronal apoptosis
In vivo	Neuroinflamation
In vivo	Neuroinflamation
In vivo	Neuroinflamation
In vivo	Autophagy dysfunction
In vivo	Autophagy dysfunction
In vivo	Autophagy dysfunction
In vitro	Neuronal apoptosis
In vitro	Neuronal apoptosis
In vitro	Neuronal apoptosis
In vivo	Brain infarction
In vivo	Brain infarction
In vivo	Neuroplasticity
In vivo	Neuroplasticity
In vivo	Neuroplasticity
In vivo	Neuroplasticity
In vivo	Neuroplasticity
In vivo	Neuroinflamation
In vivo	Neuroinflamation
In vivo	Brain infarction
In vivo	Neuroinflamation
In vivo	Neuroinflamation
In vivo	Neuroinflamation
In vivo	Neuroinflamation
In vitro	Neuroinflamation
In vitro	Neuroinflamation
In vitro	Neuroinflamation
In vitro	Neuroinflamation
In vitro	Neuroinflamation
In vitro	Neuroinflamation
In vitro	Neuroinflamation
In vitro	Neuroinflamation
In vitro	Neuroinflamation
In vitro	Neuroinflamation

In vitro	Neuroinflamation
In vitro	Neuroinflamation
In vitro	Neuronal apoptosis
In vitro	Neuronal apoptosis
In vivo	Brain infarction
In vivo	Neuronal apoptosis
In vivo	Neuronal apoptosis
In vivo	Neuronal apoptosis
In vivo	Neuronal apoptosis
In vivo	Neuronal apoptosis
In vivo	Neuronal apoptosis
In vivo	Neuronal apoptosis
In vivo	Neuronal apoptosis
In vivo	Neuronal apoptosis
In vivo	Brain infarction
In vivo	Glutamate excitotoxicity
In vivo	Glutamate excitotoxicity
In vivo	Brain infarction
In vivo	Oxidative stress
In vivo	Oxidative stress
In vivo	Neuronal apoptosis
In vivo	Neuronal apoptosis
In vivo	Brain infarction
In vivo	Neuroinflamation
In vivo	Neuroplasticity
In vivo	Neuroplasticity
In vivo	Brain infarction
In vivo	Autophagy dysfunction
In vivo	Autophagy dysfunction
In vivo	Autophagy dysfunction
In vivo	Autophagy dysfunction
In vivo	Brain infarction
In vivo	Glutamate excitotoxicity
In vivo	Neuroinflamation
In vivo	Neuroinflamation
In vivo	Neuronal apoptosis
In vivo	Brain infarction
In vivo	Neuronal apoptosis
In vivo	Neuronal apoptosis
In vivo	Neuronal apoptosis
In vivo	Oxidative stress
In vivo	Oxidative stress
In vivo	Mitochondrial damage
In vivo	Mitochondrial damage
In vitro	Neuronal apoptosis
In vitro	Neuronal apoptosis

In vitro	Neuronal apoptosis
In vivo	Brain infarction
In vivo	Neuroinflamation
In vivo	Neuroinflamation
In vivo	Neuroinflamation
In vivo	Neuroinflamation
In vivo	Oxidative stress
In vivo	Oxidative stress
In vivo	Brain infarction
In vivo	Oxidative stress
In vivo	Oxidative stress
In vivo	Oxidative stress
In vivo	Oxidative stress
In vivo	Oxidative stress
In vivo	Oxidative stress
In vivo	Neuronal apoptosis
In vivo	Neuronal apoptosis
In vivo	Neuronal apoptosis
In vivo	Neuronal apoptosis
In vivo	Neuronal apoptosis
In vivo	Neuronal apoptosis
In vivo	Neuronal apoptosis
In vivo	Neuronal apoptosis
In vitro	Brain infarction
In vitro	Integrity of BBB
In vitro	Integrity of BBB
In vitro	Mitochondrial damage
In vivo	Neuroinflamation
In vivo	Neuroinflamation
In vivo	Neuroinflamation
In vivo	Autophagy dysfunction
In vivo	Autophagy dysfunction
In vivo	Brain infarction
In vivo	Neuroinflamation
In vivo	Neuroinflamation
In vivo	Neuroinflamation
In vivo	Neuroinflamation
In vitro	Neuronal apoptosis
In vitro	Neuronal apoptosis
In vitro	Neuronal apoptosis
In vivo	Brain infarction
In vitro	Neuronal apoptosis
In vitro	Neuronal apoptosis
In vitro	Neuronal apoptosis
In vitro	Autophagy dysfunction
In vitro	Autophagy dysfunction

In vitro	Brain infarction
In vivo	Mitochondrial damage
In vivo	Mitochondrial damage
In vivo	Mitochondrial damage
In vivo	Mitochondrial damage
In vivo	Activated microglia
In vivo	Neuroinflamation
In vivo	Neuroinflamation
In vivo	Neuroinflamation
In vivo	Neuroinflamation
In vivo	Brain infarction
In vivo	Integrity of BBB
In vivo	Integrity of BBB
In vivo	Brain infarction
In vivo	Autophagy dysfunction
In vivo	Neuroinflamation
In vivo	Neuroinflamation
In vivo	Neuroinflamation
In vivo	Neuroinflamation
In vivo	Neuroinflamation
In vivo	Neuroinflamation
In vivo	Activated microglia
In vivo	Activated microglia
In vivo	Activated microglia
In vitro	Neuronal apoptosis
In vitro	Neuronal apoptosis
In vitro	Glutamate excitotoxicity
In vivo	Brain infarction
In vivo	Activated microglia
In vivo	Activated microglia
In vivo	Activated microglia
In vivo	Activated microglia
In vivo	Activated microglia
In vivo	Activated microglia
In vivo	Neuroinflamation
In vivo	Neuroinflamation
In vivo	Neuroinflamation
In vivo	Neuroinflamation
In vivo	Neuroinflamation
In vitro	Neuronal apoptosis
In vitro	Neuroinflamation
In vitro	Neuroinflamation
In vitro	Neuroinflamation
In vivo	Neuroinflamation
In vivo	Neuroinflamation
In vivo	Neuroinflamation

In vivo	Neuroinflamation
In vitro	Neuroinflamation
In vitro	Neuroinflamation
In vitro	Neuroinflamation
In vitro	Neuroinflamation
In vitro	Neuroinflamation
In vivo	Integrity of BBB
In vivo	Oxidative stress
In vivo	Oxidative stress
In vivo	Oxidative stress
In vivo	Oxidative stress
In vivo	Neuroinflamation
In vivo	Neuroinflamation
In vivo	Neuroinflamation
In vivo	Neuronal apoptosis
In vivo	Neuroinflamation
In vivo	Neuroinflamation
In vivo	Neuroinflamation
In vivo	Neuroinflamation
In vivo	Neuroinflamation
In vivo	Neuroinflamation
In vivo	Neuroinflamation
In vivo	Neuroinflamation
In vivo	Neuroinflamation
In vivo	Neuroinflamation
In vivo	Brain infarction
In vivo	Oxidative stress
In vivo	Oxidative stress
In vivo	Oxidative stress
In vivo	Neuronal apoptosis
In vivo	Neuronal apoptosis
In vivo	Neuronal apoptosis
In vivo	Neuroinflamation
In vivo	Oxidative stress
In vivo	Oxidative stress
In vivo	Neuronal apoptosis
In vivo	Brain infarction
In vivo	Neuroinflamation
In vivo	Neuroinflamation
In vivo	Neuronal apoptosis
In vivo	Neuronal apoptosis
In vivo	Neuronal apoptosis
In vivo	Neuronal apoptosis
In vivo	Neuronal apoptosis
In vivo	Neuronal apoptosis
In vivo	Neuronal apoptosis

In vivo	Neuronal apoptosis
In vivo	Neuronal apoptosis
In vivo	Brain infarction
In vivo	Neuroinflamation
In vivo	Neuroinflamation
In vivo	Neuroinflamation
In vivo	Neuroinflamation
In vivo	Neuroinflamation
In vivo	Brain infarction
In vivo	Neuronal apoptosis
In vivo	Neuronal apoptosis
In vivo	Neuronal apoptosis
In vivo	Activated microglia
In vivo	Activated microglia
In vitro	Neuronal apoptosis
In vitro	Oxidative stress
In vivo	Neuronal apoptosis
In vivo	Neuronal apoptosis
In vivo	Neuroinflamation
In vivo	Neuroinflamation
In vivo	Neuroinflamation
In vivo	Brain infarction
In vivo	Neuroinflamation
In vivo	Neuroinflamation
In vivo	Neuroinflamation
In vitro	Oxidative stress
In vivo	Brain infarction
In vivo	Oxidative stress
In vivo	Oxidative stress
In vivo	Oxidative stress
In vivo	Oxidative stress
In vivo	Oxidative stress
In vivo	Oxidative stress
In vitro	Brain infarction
In vitro	Neuronal apoptosis
In vitro	Neuronal apoptosis
In vivo	Brain infarction
In vivo	Neuroinflamation
In vivo	Neuroinflamation
In vivo	Neuroinflamation
In vivo	Brain infarction
In vitro	Neuronal apoptosis
In vitro	Neuronal apoptosis
In vitro	Oxidative stress
In vitro	Oxidative stress
In vitro	Oxidative stress

In vitro	Oxidative stress
In vivo	Neuroplasticity
In vivo	Neuroplasticity
In vivo	Brain infarction
In vivo	Neuronal apoptosis
In vivo	Neuronal apoptosis
In vivo	Neuronal apoptosis
In vivo	Neuronal apoptosis
In vivo	Neuronal apoptosis
In vivo	Neuronal apoptosis
In vivo	Neuronal apoptosis
In vivo	Neuronal apoptosis
In vivo	Neuronal apoptosis
In vivo	Neuronal apoptosis
In vivo	Neuronal apoptosis
In vivo	Neuronal apoptosis
In vivo	Brain infarction
In vivo	Oxidative stress
In vivo	Neuroplasticity
In vivo	Neuroplasticity
In vitro	Neuronal apoptosis
In vitro	Neuronal apoptosis
In vitro	Neuronal apoptosis
In vitro	Brain infarction
In vitro	Oxidative stress
In vitro	Neurotransmition
In vitro	Neuronal apoptosis
In vivo	Brain infarction
In vivo	Neurotransmition
In vivo	Neurotransmition
In vivo	Oxidative stress
In vivo	Neuroinflamation
In vivo	Brain infarction
In vivo	Brain infarction
In vivo	Activated microglia
In vivo	Activated microglia
In vivo	Neuroinflamation
In vivo	Neuroinflamation
In vivo	Neuroinflamation
In vivo	Brain infarction
In vivo	Neuroinflamation
In vivo	Neuroinflamation
In vivo	Neuroinflamation
In vivo	Neuroinflamation
In vivo	Oxidative stress
In vivo	Oxidative stress

In vivo	Neuronal apoptosis
In vivo	Neuronal apoptosis
In vivo	Neuronal apoptosis
In vivo	Oxidative stress
In vivo	Oxidative stress
In vivo	Oxidative stress
In vivo	Oxidative stress
In vivo	Oxidative stress
In vivo	Activated microglia
In vivo	Oxidative stress
In vivo	Oxidative stress
In vivo	Oxidative stress
In vivo	Oxidative stress
In vivo	Brain infarction
In vivo	Neuroplasticity
In vivo	Brain infarction
In vivo	Brain infarction
In vivo	Brain infarction
In vivo	Activated microglia
In vivo	Activated microglia
In vivo	Neuroplasticity
In vivo	Brain infarction
In vivo	Oxidative stress
In vivo	Oxidative stress
In vivo	Oxidative stress
In vivo	Oxidative stress
In vivo	Oxidative stress
In vivo	Oxidative stress
In vivo	Neuroinflamation
In vivo	Neuroinflamation
In vivo	Neuroinflamation

**Dosis**
100 and 200mg/kg
100 and 200mg/kg
100 and 200mg/kg
100 and 200mg/kg
100 mg/kg
200 mg/kg
200 mg/kg
200 mg/kg
200 mg/kg
200 mg/kg
200 mg/kg
200 mg/kg
200 mg/kg
137.5, 68.75, 34.38, 17.19, 8.59, 4.30, 2.15, and 1.08 µg/ml
137.5, 68.75, 34.38, 17.19, 8.59, 4.30, 2.15, and 1.08 µg/ml
137.5, 68.75, 34.38, 17.19, 8.59, 4.30, 2.15, and 1.08 µg/ml
50, 100 or 200 mg/kg
50, 100 or 200 mg/kg
50, 100 or 200 mg/kg
50, 100 or 200 mg/kg
200 mg/kg
200 mg/kg
200 mg/kg
200 mg/kg
200 mg/kg
200 mg/kg
200 mg/kg
200 mg/kg
200 mg/kg
100 mg/kg
100 mg/kg
100 mg/kg
100 mg/kg
100 mg/kg
100 mg/kg
100 mg/kg
100 mg/kg
100 mg/kg
2, 10, 50 µM for 30 min
2, 10, 50 µM for 30 min
2, 10, 50 µM for 30 min
2, 10, 50 µM for 30 min
2, 10, 50 µM for 30 min
2, 10, 50 µM for 30 min
2, 10, 50 µM for 30 min
2, 10, 50 µM for 30 min

0.09, 0.17, 0.35, 0.70, 1.40 mmol/kg
0.09, 0.17, 0.35, 0.70, 1.40 mmol/kg
0.09, 0.17, 0.35, 0.70, 1.40 mmol/kg
0.09, 0.17, 0.35, 0.70, 1.40 mmol/kg
0.09, 0.17, 0.35, 0.70, 1.40 mmol/kg
0.5, 2.5 or 10 nM
0.5, 2.5 or 10 nM
0.5, 2.5 or 10 nM
2 mg/Kg
2 mg/Kg
2 mg/Kg
2 mg/Kg
2 mg/Kg
2 mg/Kg
2 mg/Kg
2 mg/Kg
2 mg/Kg
2 mg/Kg
2 mg/Kg
2.5, 5, and 10 µM
2.5, 5, and 10 µM
2.5, 5, and 10 µM
25 mg/kg
25 mg/kg
40 mg/kg
40 mg/kg
40 mg/kg
40 mg/kg
40 mg/kg
10 or 30 mg kg
10 or 30 mg kg
10 or 30 mg kg
10, 30mg/kg
10, 30mg/kg
10, 30mg/kg
10, 30mg/kg
0.25 mg/L
0.25 mg/L
0.25 mg/L
0.25 mg/L
0.25 mg/L
0.25 mg/L
0.25 mg/L
0.25 mg/L
0.25 mg/L
0.25 mg/L

0.25 mg/L
0.25 mg/L
0.25 mg/L
0.25 mg/L
60 mg/kg
60 mg/kg
60 mg/kg
60 mg/kg
60 mg/kg
60 mg/kg
60 mg/kg
60 mg/kg
60 mg/kg
60 mg/kg
10 mg/kg
10 mg/kg
10 mg/kg
5, 10, and 20 mg/kg
5, 10, and 20 mg/kg
5, 10, and 20 mg/kg
5, 10, and 20 mg/kg
5, 10, and 20 mg/kg
7.5, 15, 30 mg/kg
7.5, 15, 30 mg/kg
30 mg/kg
30 mg/kg
5, 10, 20 mg/kg/d
5, 10, 20 mg/kg/d
5, 10, 20 mg/kg/d
5, 10, 20 mg/kg/d
5, 10, 20 mg/kg/d
0.5 µg/mL
10 or 50 mg/kg
10 or 50 mg/kg
10 or 50 mg/kg
10 or 50 mg/kg
20 or 40 mg/kg
20 or 40 mg/kg
20 or 40 mg/kg
20 or 40 mg/kg
20 or 40 mg/kg
20 or 40 mg/kg
75 mg/kg
75 mg/kg
0.2, 0.02, 0.002 mg/kg
0.2, 0.02, 0.002 mg/kg

0.2, 0.02, 0.002 mg/kg
40mg/kg
40mg/kg
40mg/kg
40mg/kg
20 mg/kg
20 mg/kg
20 mg/kg
10, 20 or 40 mg/kg
10, 20 or 40 mg/kg
1, 3, and 10 mg/kg
1, 3, and 10 mg/kg
1, 3, and 10 mg/kg
1, 3, and 10 mg/kg
1, 3, and 10 mg/kg
5 and 10 mg/kg
5 and 10 mg/kg
5 and 10 mg/kg
5 and 10 mg/kg
12.5, 25, and 50 mg/kg
12.5, 25, and 50 mg/kg
12.5, 25, and 50 mg/kg
12.5, 25, and 50 mg/kg
10, 20, 40 mg/kg
10, 20, 40 mg/kg
10, 20, 40 mg/kg
-
200 mg/kg
200 mg/kg
200 mg/kg
201 mg/kg
201 mg/kg
3 mg/kg
3 mg/kg
3 mg/kg
3 mg/kg
3 mg/kg
0.25-1.0 µM
0.25-1.0 µM
0.25-1.0 µM
100, 200, 300, 400 mg/kg
5, 15, 25, and 35 µmol/L
5, 15, 25, and 35 µmol/L
5, 15, 25, and 35 µmol/L
1.25–20 µM
1.25–20 µM

100 mg/kg
50, 100 mg/kg
50, 100 mg/kg
50, 100 mg/kg
50, 100 mg/kg
30 mg/kg
30 mg/kg
30 mg/kg
30 mg/kg
30 mg/kg
50 mg/kg
50 mg/kg
50 mg/kg
100 mg/kg
100 mg/kg
100 mg/kg
100 mg/kg
100 mg/kg
40 and 60 mg/kg
40 and 60 mg/kg
40 and 60 mg/kg
40 and 60 mg/kg
40 and 60 mg/kg
40 and 60 mg/kg
10 μmol/L
10 μmol/L
10 μmol/L
2.5, 5, 10, and 20 mg/kg/day
2.5, 5, 10, and 20 mg/kg/day
2.5, 5, 10, and 20 mg/kg/day
2.5, 5, 10, and 20 mg/kg/day
2.5, 5, 10, and 20 mg/kg/day
2.5, 5, 10, and 20 mg/kg/day
2.5, 5, 10, and 20 mg/kg/day
2.5, 5, 10, and 20 mg/kg/day
2.5, 5, 10, and 20 mg/kg/day
2.5, 5, 10, and 20 mg/kg/day
2.5, 5, 10, and 20 mg/kg/day
2.5, 5, 10, and 20 mg/kg/day
0, 6.25, 12.5, 25, 50, or 100 μM
0, 6.25, 12.5, 25, 50, or 100 μM
0, 6.25, 12.5, 25, 50, or 100 μM
0, 6.25, 12.5, 25, 50, or 100 μM
100 mg/kg/day
100 mg/kg/day
100 mg/kg/day

100 mg/kg/day
6, 12 μg/mL
6, 12 μg/mL
6, 12 μg/mL
6, 12 μg/mL
6, 12 μg/mL
25, 75, 125 mg/kg
200mg/l
5, 10 or 20 mg/kg per day
5, 10 or 20 mg/kg per day
5, 10 or 20 mg/kg per day
10 mg/kg
10 mg/kg
10 mg/kg
10 mg/kg
20 mg/kg
20 mg/kg
20 mg/kg
20 mg/kg
20 mg/kg
20 mg/kg
20 mg/kg
20 mg/kg
20 mg/kg
20 mg/kg
20 mg/kg
20 mg/kg
20 mg/kg
20 mg/kg
10, 20, 40 mg/kg
10, 20, 40 mg/kg
10, 20, 40 mg/kg
20, 40, 80 mg/kg
20, 40, 80 mg/kg
20, 40, 80 mg/kg
20, 40, 80 mg/kg
10 or 20 mg/kg
10 or 20 mg/kg
10 and 20mg/kg
12.5, 25, and 50 mg/kg
12.5, 25, and 50 mg/kg
12.5, 25, and 50 mg/kg
12.5, 25, and 50 mg/kg
12.5, 25, and 50 mg/kg
12.5, 25, and 50 mg/kg
12.5, 25, and 50 mg/kg

12.5, 25, and 50 mg/kg
12.5, 25, and 50 mg/kg
10 ml/kg
10 ml/kg
10 ml/kg
10 ml/kg
10 ml/kg
10 ml/kg
50, 100, and 200 mg/kg
50, 100, and 200 mg/kg
50, 100, and 200 mg/kg
50, 100, and 200 mg/kg
10 or 100 μg/kg
10 or 100 μg/kg
20-100 μM
10 μM
120 mg/kg
120 mg/kg
120 mg/kg
120 mg/kg
120 mg/kg
2. 5, 5, 10 mg/kg
2. 5, 5, 10 mg/kg
2. 5, 5, 10 mg/kg
2. 5, 5, 10 mg/kg
25 mg/L
1 mg/kg, 2 mg/kg, and 4 mg/kg
1 mg/kg, 2 mg/kg, and 4 mg/kg
1 mg/kg, 2 mg/kg, and 4 mg/kg
1 mg/kg, 2 mg/kg, and 4 mg/kg
1 mg/kg, 2 mg/kg, and 4 mg/kg
1 mg/kg, 2 mg/kg, and 4 mg/kg
1 mg/kg, 2 mg/kg, and 4 mg/kg
10 µmol/L
10 µmol/L
10 µmol/L
1.25 mg/kg
1.25 mg/kg
1.25 mg/kg
1.25 mg/kg
7.8, 15.6, 31.2 mg/kg
1 μM, 3 μM, 10 μM, 50 μM, 100 μM
1 μM, 3 μM, 10 μM, 50 μM, 100 μM
0.003 to 0.3 μM
0.003 to 0.3 μM
0.003 to 0.3 μM

0.003 to 0.3 µM
0.2 g/kg
0.2 g/kg
0.2 g/kg
0.2 g/kg
0.2 g/kg
0.2 g/kg
0.2 g/kg
0.2 g/kg
0.2 g/kg
0.2 g/kg
0.2 g/kg
0.2 g/kg
0.2 g/kg
0.2 g/kg
0.2 g/kg
20 and 30 mg/kg
20 and 30 mg/kg
20 and 30 mg/kg
20 and 30 mg/kg
10–40 mM
10–40 mM
10–40 mM
15mg/kg
15mg/kg
15mg/kg
15mg/kg
10 mg/kg, 20 mg/kg
10 mg/kg, 20 mg/kg
10 mg/kg, 20 mg/kg
10 mg/kg, 20 mg/kg
10 mg/kg, 20 mg/kg
10 mg/kg
10 mg/kg
10 mg/kg
10 mg/kg
10 mg/kg
10 mg/kg
10 mg/kg
80 mg/kg or 160 mg/kg
80 mg/kg or 160 mg/kg
80 mg/kg or 160 mg/kg
80 mg/kg or 160 mg/kg
80 mg/kg or 160 mg/kg
80 mg/kg or 160 mg/kg
80 mg/kg or 160 mg/kg

80 mg/kg or 160 mg/kg
80 mg/kg or 160 mg/kg
80 mg/kg or 160 mg/kg
50 mg/kg
50 mg/kg
50 mg/kg
50 mg/kg
50 mg/kg
50 mg/kg
50 mg/kg
50 mg/kg
50 mg/kg
50 mg/kg
10 mg/kg
10 mg/kg
1 mg/kg
3, 10, and 30 mg/kg
10mg/kg
10mg/kg
10mg/kg
10mg/kg
10-30 mg/kg
10-30 mg/kg
10-30 mg/kg
10-30 mg/kg
10-30 mg/kg
10-30 mg/kg
10-30 mg/kg
10-30 mg/kg
10-30 mg/kg
10-30 mg/kg

Activity
Inhibited the expression of Caspase-3
Inhibited the expression of Caspase-3
Promoted the expression of BDNF
Promoted the expression of BDNF
Attenuating the phosphorylation level of CaMKII and prevented OGD-induced apoptosis of hippocampal or SH-
Increase of GABA(A)R α1, GABA(A)R γ2 and KCC2 mRNA and protein levels
Increase of GABA(A)R α1, GABA(A)R γ2 and KCC2 mRNA and protein levels
Increase of GABA(A)R α1, GABA(A)R γ2 and KCC2 mRNA and protein levels
Increase of GABA(A)R α1, GABA(A)R γ2 and KCC2 mRNA and protein levels
Increase of GABA(A)R α1, GABA(A)R γ2 and KCC2 mRNA and protein levels
Increase of GABA(A)R α1, GABA(A)R γ2 and KCC2 mRNA and protein levels
Decrease of NKCC1 at mRNA and protein levels
Decrease of NKCC1 at mRNA and protein levels
Increase the release of PI3K,Akt via MAPK/ERK
Increase the release of PI3K,Akt via MAPK/ERK
Increased levels of BDNF
Enhancing BCL-2 and MCL-1 expression by triggering the CArG box
Enhancing BCL-2 and MCL-1 expression by triggering the CArG box
Enhancing BCL-2 and MCL-1 expression by triggering the CArG box
Enhancing BCL-2 and MCL-1 expression by triggering the CArG box
Suppressed NF-κB signaling by reducing IκBα phosphorylation and nuclear translocation of NF-κB/p65, which d
Suppressed NF-κB signaling by reducing IκBα phosphorylation and nuclear translocation of NF-κB/p65, which d
Suppressed NF-κB signaling by reducing IκBα phosphorylation and nuclear translocation of NF-κB/p65, which d
Increased the Bcl-2 expression
Reduced Bax and caspase-3 expression
Reduced Bax and caspase-3 expression
Increase expression of the PI3K, Akt, mTOR proteins
Increase expression of the PI3K, Akt, mTOR proteins
Increase expression of the PI3K, Akt, mTOR proteins
Enhanced expression of MAP-2, BDNF, NT-3 and IGF-1
Enhanced expression of MAP-2, BDNF, NT-3 and IGF-1
Enhanced expression of MAP-2, BDNF, NT-3 and IGF-1
Enhanced expression of MAP-2, BDNF, NT-3 and IGF-1
Suppressed expression of proinflammatory mediators (iNOS, TNF-α, and IL-1β) in in activated microglia/brain
Suppressed expression of proinflammatory mediators (iNOS, TNF-α, and IL-1β) in in activated microglia/brain
Suppressed expression of proinflammatory mediators (iNOS, TNF-α, and IL-1β) in in activated microglia/brain
Decreased expression of proinflammatory markers in AM/BM through suppressing the p-JNK and p-p38 MAPK
Decreased expression of proinflammatory markers in AM/BM through suppressing the p-JNK and p-p38 MAPK
Inhibited LPS-induced production of proinflammatory mediators such as NO, TNFα, IL-1β and ROS
Inhibited LPS-induced production of proinflammatory mediators such as NO, TNFα, IL-1β and ROS
Inhibited LPS-induced production of proinflammatory mediators such as NO, TNFα, IL-1β and ROS
Inhibited LPS-induced production of proinflammatory mediators such as NO, TNFα, IL-1β and ROS
Inhibited LPS-induced production of proinflammatory mediators such as NO, TNFα, IL-1β and ROS
Inhibited LPS-induced production of proinflammatory mediators such as NO, TNFα, IL-1β and ROS
Inhibited LPS-induced production of proinflammatory mediators such as NO, TNFα, IL-1β and ROS
Inhibited LPS-induced production of proinflammatory mediators such as NO, TNFα, IL-1β and ROS

Decrease the contents of glutamic acid (Glu), aspartic acid (Asp), glycine (Gly), γ-aminobutyric acid (GABA) and
Decrease the contents of glutamic acid (Glu), aspartic acid (Asp), glycine (Gly), γ-aminobutyric acid (GABA) and
Decrease the contents of glutamic acid (Glu), aspartic acid (Asp), glycine (Gly), γ-aminobutyric acid (GABA) and
Decrease the contents of glutamic acid (Glu), aspartic acid (Asp), glycine (Gly), γ-aminobutyric acid (GABA) and
Decrease the contents of glutamic acid (Glu), aspartic acid (Asp), glycine (Gly), γ-aminobutyric acid (GABA) and
Reduced LDH and Caspase 3 level
Reduced LDH and Caspase 3 level
Increase eNOS phosphorylation and inhibit iNOS expression in response to the activated Akt
MCAO-induced brain infarction was decreased by vitexin
Increase protein levels of antigen identified by monoclonal antibody (Ki-67) and Bcl-2
Increase protein levels of antigen identified by monoclonal antibody (Ki-67) and Bcl-2
Decrease protein levels of Caspase-3 and Bax, antigen identified by monoclonal antibody (Ki-67) and Bcl-2 in M
Decrease protein levels of Caspase-3 and Bax, antigen identified by monoclonal antibody (Ki-67) and Bcl-2 in M
Increased IL-10 production to ameliorate MCAO-induced inflammation
Attenuated the secretion of pro-inflammatory cytokine IL-6 and TNF-a to ameliorate MCAO-induced inflammat
Attenuated the secretion of pro-inflammatory cytokine IL-6 and TNF-a to ameliorate MCAO-induced inflammat
Enhancive expression of Ulk1 and inhibited the reduced expression of mTOR and PPAR-γ
Enhancive expression of Ulk1 and inhibited the reduced expression of mTOR and PPAR-γ
Enhancive expression of Ulk1 and inhibited the reduced expression of mTOR and PPAR-γ
Upregulated mRNA expression of Caspase-3 in the HBMVEC + KD group compared with the HBMVEC + NC grou
Attenuated expression of Bcl-2, Caveolin-1-KD in the HBMVEC and HBMVEC + NC groups
Attenuated expression of Bcl-2, Caveolin-1-KD in the HBMVEC and HBMVEC + NC groups
Reduced volume of cerebral infarction while prompting vascular endothelial cell proliferation by upregulating
Reduced volume of cerebral infarction while prompting vascular endothelial cell proliferation by upregulating
The acetylated H3 and acetylated H4 expressions and Syn-I expressions in the hippocampus was upregulated
The acetylated H3 and acetylated H4 expressions and Syn-I expressions in the hippocampus was upregulated
The acetylated H3 and acetylated H4 expressions and Syn-I expressions in the hippocampus was upregulated
The HDAC content was decreased
The BDNF level was markedly increased
Inhibited IL-1β and TGF-β1 protein expression
Inhibited IL-1β and TGF-β1 protein expression
Improved the neurological dysfunction and decreased infarct volume in MCAO rats.
Reduce the protein levels of IL-1β and TGF-β1
Reduce the protein levels of IL-1β and TGF-β1
Up-regulated PPARα and PPARγ protein levels and suppressed IκB-α degradation and NF-κB activation induced
Up-regulated PPARα and PPARγ protein levels and suppressed IκB-α degradation and NF-κB activation induced
Reduce the the expression of IL-1 β, IL-6, TNF-α, XBP1u, XBP1s and caspase-3
Reduce the the expression of IL-1 β, IL-6, TNF-α, XBP1u, XBP1s and caspase-3
Reduce the the expression of IL-1 β, IL-6, TNF-α, XBP1u, XBP1s and caspase-3
Reduce the the expression of IL-1 β, IL-6, TNF-α, XBP1u, XBP1s and caspase-3
Reduce the the expression of IL-1 β, IL-6, TNF-α, XBP1u, XBP1s and caspase-3
Reduce the the expression of IL-1 β, IL-6, TNF-α, XBP1u, XBP1s and caspase-3
Reduce the the expression of IL-1 β, IL-6, TNF-α, XBP1u, XBP1s and caspase-3
Reduce the the expression of IL-1 β, IL-6, TNF-α, XBP1u, XBP1s and caspase-3
Reduce the the expression of IL-1 β, IL-6, TNF-α, XBP1u, XBP1s and caspase-3
Reduce the the expression of IL-1 β, IL-6, TNF-α, XBP1u, XBP1s and caspase-3

Reduce the the expression of IL-1 β, IL-6, TNF-α, XBP1u, XBP1s and caspase-3
Reduce the the expression of IL-1 β, IL-6, TNF-α, XBP1u, XBP1s and caspase-3
Reduce the overexpression of p-IRE1α related with ER stress and apoptosis
Reduce the overexpression of p-IRE1α related with ER stress and apoptosis
Icariin enhanced the effect of therapeutic hypothermia on infarct and neurological deficits in MCAO rats
Downregulated the expressions of TNF-α, IL-6, C-Caspase 3 and Bax, and the activation of PPARs/Nrf2/NF-κB ar
Downregulated the expressions of TNF-α, IL-6, C-Caspase 3 and Bax, and the activation of PPARs/Nrf2/NF-κB ar
Downregulated the expressions of TNF-α, IL-6, C-Caspase 3 and Bax, and the activation of PPARs/Nrf2/NF-κB ar
Downregulated the expressions of TNF-α, IL-6, C-Caspase 3 and Bax, and the activation of PPARs/Nrf2/NF-κB ar
Downregulated the expressions of TNF-α, IL-6, C-Caspase 3 and Bax, and the activation of PPARs/Nrf2/NF-κB ar
Downregulated the expressions of TNF-α, IL-6, C-Caspase 3 and Bax, and the activation of PPARs/Nrf2/NF-κB ar
Downregulated the expressions of TNF-α, IL-6, C-Caspase 3 and Bax, and the activation of PPARs/Nrf2/NF-κB ar
Downregulated the expressions of TNF-α, IL-6, C-Caspase 3 and Bax, and the activation of PPARs/Nrf2/NF-κB ar
Elevated the expression of Bcl-2
quercetin administration attenuated the permanent middle cerebral artery occlusion (pMCAO)-induced increa
Quercetin treatment attenuated the increase of caspase-3
Quercetin treatment attenuated the increase of caspase-3
Lower degree of infarct volume, and brain water content than the vehicle-treated rats
Decreased the ROS and MDA production, and increased the activity of SOD and CAT in brains of MCAO/R rats
Decreased the ROS and MDA production, and increased the activity of SOD and CAT in brains of MCAO/R rats
Downregulation of cleaved caspase-3 protein
Upregulation of Bcl-2 protein level
Ameliorated neurologic deficit and infarct volume after cerebral ischemia reperfusion
Inhibit the expression of 4-Hydroxy-2-nonenal (4-HNE).
Increased the cerebral expression of BDNF/TrkB,
Increased the cerebral expression of BDNF/TrkB,
Decreased infarct volume and brain edema, and ameliorated neurological scores
Upregulated the expression of p62, NBR1 and Bcl-2
Upregulated the expression of p62, NBR1 and Bcl-2
Upregulated the expression of p62, NBR1 and Bcl-2
Downregulated the level of TNF-α.
treatment of XN improved MCAO-induced brain injury by reducing infarct size, improving neurological deficits
Ameliorated glutamate concentration elevation, and CA3 neuron death
Decrease of mitochondrial fusion protein Mfn-2 and antiapoptotic protein Bcl-2 expression
Decrease of mitochondrial fusion protein Mfn-2 and antiapoptotic protein Bcl-2 expression
Inhibition of Apaf-1 expression and caspase-3 activation
Attenuated the neurological deficit score, brain water content and infarct area
Decreased serum TNF-α, IL-1β and IL-6 concentrations
Decreased serum TNF-α, IL-1β and IL-6 concentrations
Decreased serum TNF-α, IL-1β and IL-6 concentrations
Decrease levels of ACSL4 and TFR1 in the cortex that attenuates ferroptosis
Decrease levels of ACSL4 and TFR1 in the cortex that attenuates ferroptosis
Stimulated expression PGC-1α and TFAM that are responsible for mitochondrial biogenesis mitochondrial and
Stimulated expression PGC-1α and TFAM that are responsible for mitochondrial biogenesis mitochondrial and
Upregulated Akt activation by and suppressed cleaved caspase-3 expression.
Upregulation of PI3K, p55γ mRNA in vitro and in vivo

Upregulation of PI3K, p55γ mRNA in vitro and in vivo

Infarct volume decreased, decreased brain edema and contributed to the restoration of motor function

Downregulation of pro-inflammatory cytokines (TNFα and IL-6)

Downregulation of pro-inflammatory cytokines (TNFα and IL-6)

Upregulation anti-inflammatory cytokines (IL-1β)

Minimal detection of CD68-positive activated macrophages/microglia

Upregulated Akt and ERK phosphorylation, and attenuated neutrophil migration, endothelium adhesion, spont

Upregulated Akt and ERK phosphorylation, and attenuated neutrophil migration, endothelium adhesion, spont

Reducing volume of infarction, neuronal loss and water content

The nNOS expression in cortex and dentate gyrus was reduced

Increased levels of NO, p-Akt/Akt, and p-eNOS/eNOS

Increased levels of NO, p-Akt/Akt, and p-eNOS/eNOS

Increased levels of NO, p-Akt/Akt, and p-eNOS/eNOS

Increased levels of NO, p-Akt/Akt, and p-eNOS/eNOS

Increased levels of NO, p-Akt/Akt, and p-eNOS/eNOS

Increased Bcl-2 expression

Increased Bcl-2 expression

Reduced Bax expression in the penumbra

Reduced Bax expression in the penumbra

Enhanced the expression of Bcl-2

Attenuated the content of Bax, cleaved caspase-3, and PARP

Attenuated the content of Bax, cleaved caspase-3, and PARP

Attenuated the content of Bax, cleaved caspase-3, and PARP

Brain water content increase, cerebral blood flow decrease, cerebral infarction and neurological deficits decrea

Inhibits the expression of MMP-2/9 and Src/MLCK/MLC signaling pathway to attenuated BBB injury.

Inhibits the expression of MMP-2/9 and Src/MLCK/MLC signaling pathway to attenuated BBB injury.

Increased mitochondrial respiration, prevented mitochondrial dysfunction by the increased the expression of [illegible]

Decreased protein levels of TLR4, p-p38, and IL1

Decreased protein levels of TLR4, p-p38, and IL1

Decreased protein levels of TLR4, p-p38, and IL1

Upregulating p-Akt and p-mTOR expression

Upregulating p-Akt and p-mTOR expression

Attenuated neurological deficits, reduced cerebral infarction area and lowered brain water content

Reduced the epression of IL-1β and IL-8 and enhanced the expression of p-JAK2 and p-STAT3

Reduced the epression of IL-1β and IL-8 and enhanced the expression of p-JAK2 and p-STAT3

Reduced the epression of IL-1β and IL-8 and enhanced the expression of p-JAK2 and p-STAT3

Reduced the epression of IL-1β and IL-8 and enhanced the expression of p-JAK2 and p-STAT3

Upregulation of flotillin-1 protein level wich decrease cell death, LDH release and caspase-3 activation

Upregulation of flotillin-1 protein level wich decrease cell death, LDH release and caspase-3 activation

Upregulation of flotillin-1 protein level wich decrease cell death, LDH release and caspase-3 activation

Reduced infarct volume, improved neurological function, alleviated the morphological damage of neurons, and

Increased Bcl-2 protein levels

Decreased Bax and caspase-3 expressions in mouse N2a cells

Decreased Bax and caspase-3 expressions in mouse N2a cells

Decreased the expression of LC3 II and HIF-1α

Decreased the expression of LC3 II and HIF-1α

Exhibited substantial reductions in neurological score and infarct area

Upregulated expression of the mitochondrial biogenesis regulators NRF-1 and TFAM

Upregulated expression of the mitochondrial biogenesis regulators NRF-1 and TFAM

Upregulated expression of the mitochondrial biogenesis regulators NRF-1 and TFAM

Upregulated expression of the mitochondrial biogenesis regulators NRF-1 and TFAM

Reduced astroglial and microglial activation associated with a decrease in cd11b immunoreactivity

Attenuated NF-κB levels and JNK activation; Pretreatment for 7 days before I/R decreased iNOS and COX-2 exp

Attenuated NF-κB levels and JNK activation; Pretreatment for 7 days before I/R decreased iNOS and COX-2 exp

Attenuated NF-κB levels and JNK activation; Pretreatment for 7 days before I/R decreased iNOS and COX-2 exp

Attenuated NF-κB levels and JNK activation; Pretreatment for 7 days before I/R decreased iNOS and COX-2 exp

Reduced the cerebral ischemia reperfusion damage, brain edema, and Evans blue dye extravasation

Downregulated MMP-9 expression

Upregulated TIMP-1 expression

Reduced cerebral infarct volume, decreased brain water content, and improved neurological scores

Upregulates Sirt1 expression and enhence autophagy activity

Reduce expression of protein levels of activated NLRP3 inflammasome (caspase-1, IL-1β, and IL-18)

Reduce expression of protein levels of activated NLRP3 inflammasome (caspase-1, IL-1β, and IL-18)

Reduce expression of protein levels of activated NLRP3 inflammasome (caspase-1, IL-1β, and IL-18)

Decreased expression of inflammatory cytokines including interleukin (IL)-1β, interleukin (IL)-6 and tumor necr

Decreased expression of inflammatory cytokines including interleukin (IL)-1β, interleukin (IL)-6 and tumor necr

Decreased expression of inflammatory cytokines including interleukin (IL)-1β, interleukin (IL)-6 and tumor necr

Reduced phosphorylation of p38, MAPK and Iba1 expression

Reduced phosphorylation of p38, MAPK and Iba1 expression

Reduced phosphorylation of p38, MAPK and Iba1 expression

Increase ilevels of Bax

Decreased levels of Bcl-2

Attenuated the upregulation of NR2B expression, a subunit of N-methyl-D-aspartate receptors and inhibited Ca

Reduced cerebral infarction and improved neurological function after cerebral ischemia

Reduced the expression of M1 microglia/macrophage markers (IL-1β, IL-6, TNF-α, IFN-γ)

Reduced the expression of M1 microglia/macrophage markers (IL-1β, IL-6, TNF-α, IFN-γ)

Reduced the expression of M1 microglia/macrophage markers (IL-1β, IL-6, TNF-α, IFN-γ)

Reduced the expression of M1 microglia/macrophage markers (IL-1β, IL-6, TNF-α, IFN-γ)

Increased the expression of M2 microglia/macrophage markers after stroke (IL-10, TGFβ)

Increased the expression of M2 microglia/macrophage markers after stroke (IL-10, TGFβ)

Reduced the secretion of the pro-inflammatory cytokines, IL-1β, IL-2, IL-6, IL-8, and TNFα in M1 microglia

Reduced the secretion of the pro-inflammatory cytokines, IL-1β, IL-2, IL-6, IL-8, and TNFα in M1 microglia

Reduced the secretion of the pro-inflammatory cytokines, IL-1β, IL-2, IL-6, IL-8, and TNFα in M1 microglia

Reduced the secretion of the pro-inflammatory cytokines, IL-1β, IL-2, IL-6, IL-8, and TNFα in M1 microglia

Reduced the secretion of the pro-inflammatory cytokines, IL-1β, IL-2, IL-6, IL-8, and TNFα in M1 microglia

Increased viability, inhibited lactate dehydrogenase (LDH) release, and reduced apoptosis in OGD/R-induced B

Reduce levels of TNF-α, IL-6 and IL-8

Reduce levels of TNF-α, IL-6 and IL-8

Reduce levels of TNF-α, IL-6 and IL-8

Reduce NFκB p50 subunit, IL-6, and TNFα expression

Reduce NFκB p50 subunit, IL-6, and TNFα expression

Reduce NFκB p50 subunit, IL-6, and TNFα expression

Increase NeuN expression
Decreased levels of NLRP3, ASC, cleaved- caspase-1 and IL-1β, and decreased the expression of P62
Decreased levels of NLRP3, ASC, cleaved- caspase-1 and IL-1β, and decreased the expression of P62
Decreased levels of NLRP3, ASC, cleaved- caspase-1 and IL-1β, and decreased the expression of P62
Decreased levels of NLRP3, ASC, cleaved- caspase-1 and IL-1β, and decreased the expression of P62
Decreased levels of NLRP3, ASC, cleaved- caspase-1 and IL-1β, and decreased the expression of P62
protected the tMCAO animals against vascular remodelling and controled hemorhragic lesion
Medium and high doses of geniposide attenuated oxidative stress insult and improved iLTP
Increased the activity superoxide dismutase (SOD) and decresed malondialdehyde (MDA) content and plasma l
Increased the activity superoxide dismutase (SOD) and decresed malondialdehyde (MDA) content and plasma l
Increased the activity superoxide dismutase (SOD) and decresed malondialdehyde (MDA) content and plasma l
Down-regulate the expressions of TLR4, NFkappaB and TNFalpha
Down-regulate the expressions of TLR4, NFkappaB and TNFalpha
Down-regulate the expressions of TLR4, NFkappaB and TNFalpha
Inhibit caspase-3 expression and reduce PARP degradation after cerebral ischemic
Decrease levels of pMEK1/2, pERK1/2, and COX2 downregulated expression
Decrease levels of pMEK1/2, pERK1/2, and COX2 downregulated expression
Decrease levels of pMEK1/2, pERK1/2, and COX2 downregulated expression
Decrease levels of pMEK1/2, pERK1/2, and COX2 downregulated expression
Decrease levels of pMEK1/2, pERK1/2, and COX2 downregulated expression
Decrease levels of pMEK1/2, pERK1/2, and COX2 downregulated expression
Decrease levels of pMEK1/2, pERK1/2, and COX2 downregulated expression
Decrease levels of pMEK1/2, pERK1/2, and COX2 downregulated expression
Decrease levels of pMEK1/2, pERK1/2, and COX2 downregulated expression
Decrease levels of pMEK1/2, pERK1/2, and COX2 downregulated expression
Decrease the cerebral infarction volume
Reduce ROS contents, protect the structure of mitochondria, and down-regulate the expression of CytC and Ca
Reduce ROS contents, protect the structure of mitochondria, and down-regulate the expression of CytC and Ca
Reduce ROS contents, protect the structure of mitochondria, and down-regulate the expression of CytC and Ca
Increased the expression of p-ERK1/2 and p-JNK
Increased the expression of p-ERK1/2 and p-JNK
Increased the expression of p-ERK1/2 and p-JNK
Reduced IL-1 beta epression
Increase SOD activity and decrease MDA content in serum and spinal cord tissue
Increase SOD activity and decrease MDA content in serum and spinal cord tissue
Increase survivin protein expression and decrease neuronal apoptosis
Improved neurological outcome and reduced infarct volume
Decreasing the percentage of CD11b/CD18-positive neutrophils and down-regulating the expression of interce
Reduced the expression of toll-like receptor-4 (TLR4) and its downstream adaptor proteins (MyD88, TRIF and T
Inhibited the mRNA upregulation of Fas, FasL, Caspase-8, and Bax/Bcl-2
Inhibited the mRNA upregulation of Fas, FasL, Caspase-8, and Bax/Bcl-2
Inhibited the mRNA upregulation of Fas, FasL, Caspase-8, and Bax/Bcl-2
Inhibited the mRNA upregulation of Fas, FasL, Caspase-8, and Bax/Bcl-2
Inhibited the mRNA upregulation of Fas, FasL, Caspase-8, and Bax/Bcl-2
Increase protein level of apoptosis cytokines Caspase-8, Bid, cleaved Caspase-3 and Cyto C
Increase protein level of apoptosis cytokines Caspase-8, Bid, cleaved Caspase-3 and Cyto C

Increase protein level of apoptosis cytokines Caspase-8, Bid, cleaved Caspase-3 and Cyto C

Increase protein level of apoptosis cytokines Caspase-8, Bid, cleaved Caspase-3 and Cyto C

Reduced the cerebral infarct volume and the neurological deficit score

Reduced the protein levels of NLRP3, Caspase-1, IL-1β, GSDMD, and GSDMD-N, inhibiting NLRP3 inflammasom

Reduced the protein levels of NLRP3, Caspase-1, IL-1β, GSDMD, and GSDMD-N, inhibiting NLRP3 inflammasom

Reduced the protein levels of NLRP3, Caspase-1, IL-1β, GSDMD, and GSDMD-N, inhibiting NLRP3 inflammasom

Reduced the protein levels of NLRP3, Caspase-1, IL-1β, GSDMD, and GSDMD-N, inhibiting NLRP3 inflammasom

Reduced the protein levels of NLRP3, Caspase-1, IL-1β, GSDMD, and GSDMD-N, inhibiting NLRP3 inflammasom

Reduce the severity of neurological deficits, infarct volumes, cerebral edema, and neuronal death

Reduce the level of cleaved caspase-3 and Increase the Bcl-2/Bax ratio

Reduce the level of cleaved caspase-3 and Increase the Bcl-2/Bax ratio

Reduce the level of cleaved caspase-3 and Increase the Bcl-2/Bax ratio

Reduces NOX2 and iNOS expression by impairing PI3K/AKT-dependent NF- κB and HIF-1 α activation

Reduces NOX2 and iNOS expression by impairing PI3K/AKT-dependent NF- κB and HIF-1 α activation

Induced CEC apoptosis and caspase-3 activation

Increased Nrf2-HO-1 expression through p38 MAPK regulation

Decreased the expression of GFAP

Increased the expression of NeuN

Decreased the levels of pro-inflammatory cytokines (IL-1β, IL-6 and TNF-α)

Decreased the levels of pro-inflammatory cytokines (IL-1β, IL-6 and TNF-α)

Decreased the levels of pro-inflammatory cytokines (IL-1β, IL-6 and TNF-α)

Reduced infarct volume, ameliorated the neurological deficit and the permeability of BBB

Decresed the content of IL-6, IL-1beta in serum and TNF-alpha

Decresed the content of IL-6, IL-1beta in serum and TNF-alpha

Decresed the content of IL-6, IL-1beta in serum and TNF-alpha

Upregulated the protein levels of HO-1, Nqo1, SOD, p-Akt, p-Nrf2, SOD, and Nrf2

The infarct volume ratios in MCAO rats were dramatically decreased by GB in a dose-dependent

Upregulated the protein levels of HO-1, Nqo1, SOD, p-Akt, p-Nrf2, SOD, and Nrf2

Upregulated the protein levels of HO-1, Nqo1, SOD, p-Akt, p-Nrf2, SOD, and Nrf3

Upregulated the protein levels of HO-1, Nqo1, SOD, p-Akt, p-Nrf2, SOD, and Nrf4

Upregulated the protein levels of HO-1, Nqo1, SOD, p-Akt, p-Nrf2, SOD, and Nrf5

Upregulated the protein levels of HO-1, Nqo1, SOD, p-Akt, p-Nrf2, SOD, and Nrf6

Upregulated the protein levels of HO-1, Nqo1, SOD, p-Akt, p-Nrf2, SOD, and Nrf7

Decreased rat neurological deficit scores, reduced brain infarct volume

Reduced the ratio of Bax/Bcl-2 in the I/R group

Reduced the ratio of Bax/Bcl-2 in the I/R group

Reduced infarct size and brain edema, improved neurological deficit score and inhibit platelet aggregation and

Decreasing lactate dehydrogenase (LDH) activity, and inhibiting IL-1β and TNF-α releasing associated with the

Decreasing lactate dehydrogenase (LDH) activity, and inhibiting IL-1β and TNF-α releasing associated with the

Decreasing lactate dehydrogenase (LDH) activity, and inhibiting IL-1β and TNF-α releasing associated with the

Reduced the brain infarct and edema, attenuated the neuro-behavioral dysfunction, and improved cell morph

Decreased expression of bcl-2, and bax via activating PI3K/Akt signaling pathway

Decreased expression of bcl-2, and bax via activating PI3K/Akt signaling pathway

Inhibits overproduction of ROS and iNOS/NO and decreased TNF-alpha levels

Inhibits overproduction of ROS and iNOS/NO and decreased TNF-alpha levels

Inhibits overproduction of ROS and iNOS/NO and decreased TNF-alpha levels

Inhibits overproduction of ROS and iNOS/NO and decreased TNF-alpha levels
Increased serum VEGF levels and decreased serum TNF-α levels
Increased serum VEGF levels and decreased serum TNF-α levels
Ameliorated neurological deficits
Reducing the Bax/Bcl-2 ratio
Reducing the Bax/Bcl-2 ratio
Reducing the Bax/Bcl-2 ratio
Reducing the Bax/Bcl-2 ratio
Reduce the gene and protein levels of Apaf-1, Bad and Caspase-3 and increase the expression of Bcl-2
Reduce the gene and protein levels of Apaf-1, Bad and Caspase-3 and increase the expression of Bcl-2
Reduce the gene and protein levels of Apaf-1, Bad and Caspase-3 and increase the expression of Bcl-2
Increase the expression of Bcl-2
Reduce the gene and protein levels of Apaf-1, Bad and Caspase-3 and increase the expression of Bcl-2
Reduce the gene and protein levels of Apaf-1, Bad and Caspase-3 and increase the expression of Bcl-2
Reduce the gene and protein levels of Apaf-1, Bad and Caspase-3 and increase the expression of Bcl-2
Increase the expression of Bcl-2
Improved neurological deficits, infarct volume, and brain edema
Reduced the cleaved Caspase-3 level but enhanced the phosphorylation of Akt, BAD and mTOR
Enhanced the expression of BDNF and CREB significantly
Enhanced the expression of BDNF and CREB significantly
Exposure of glutamate-treated cells to emodin induced an increase in the level of Bcl-2 expression
Increase in the level of Bcl-2 expression
Reduce expression of Bax and active caspase-3 proteins
Reduced infarct volume and cell death
Reduced reactive oxygen species (ROS) production and glutamate release
Increased glutamate transporter-1 (GLT-l) expression and reduce glutamate release
Increased Bcl-2 expression
Prevent postischemic neuronal degeneration and brain slice injury
Decrease the extracellular level of glutamate and aspartate significantly
Decrease the extracellular level of glutamate and aspartate significantly
Decrease the level of malondialdehyde (MDA) and Increase the activity of superoxide dismutase (SOD)
Inhibit expression of matrix metalloproteinase-3(MMP-3)
Prevented the reduction on adenosine A1 receptor level caused by ischemia
Ameliorated long-term neurological deficits and reduced neuronal tissue loss
Ihibited microglia/macrophage pro-inflammatory polarization by downregulated of CD16/Iba1
Ihibited microglia/macrophage pro-inflammatory polarization by downregulated of CD16/Iba1
Inhibits the expression of inflammatory mediators (TNF-α, IL-1β, and CCL3) and suppressed the activities of M[illegible]
Inhibits the expression of inflammatory mediators (TNF-α, IL-1β, and CCL3) and suppressed the activities of M[illegible]
Inhibits the expression of inflammatory mediators (TNF-α, IL-1β, and CCL3) and suppressed the activities of M[illegible]
Reduced the neurological deficits and infarct volume
Decreased the levels of inflammation-associated cytokines IL-1β, IL-6, myeloperoxidase (MPO), and TNF-α
Decreased the levels of inflammation-associated cytokines IL-1β, IL-6, myeloperoxidase (MPO), and TNF-α
Decreased the levels of inflammation-associated cytokines IL-1β, IL-6, myeloperoxidase (MPO), and TNF-α
Decreased the levels of inflammation-associated cytokines IL-1β, IL-6, myeloperoxidase (MPO), and TNF-α
Decreased the levels of oxidative stress-related proteins (MDA and SOD)
Decreased the levels of oxidative stress-related proteins (MDA and SOD)

Decreased the levels of apoptosis-related proteins (p-53, Bax, and B-cell lymphoma (Bcl)-2)
Decreased the levels of apoptosis-related proteins (p-53, Bax, and B-cell lymphoma (Bcl)-2)
Decreased the levels of apoptosis-related proteins (p-53, Bax, and B-cell lymphoma (Bcl)-2)
Reduce oxidative stress indicators (dihydroethidium, 8-hydroxyguanine and 4-hydroxy-2-nonenal) and antioxid
Reduce oxidative stress indicators (dihydroethidium, 8-hydroxyguanine and 4-hydroxy-2-nonenal) and antioxid
Reduce oxidative stress indicators (dihydroethidium, 8-hydroxyguanine and 4-hydroxy-2-nonenal) and antioxid
Reduce oxidative stress indicators (dihydroethidium, 8-hydroxyguanine and 4-hydroxy-2-nonenal) and antioxid
Reduce oxidative stress indicators (dihydroethidium, 8-hydroxyguanine and 4-hydroxy-2-nonenal) and antioxid
Inhibited activations of astrocytes and microglia (GFAP) in the ischemic CA1 area
Reduced the increased 4-hydroxy-2-noneal and superoxide anion radical production and increased expressions
Reduced the increased 4-hydroxy-2-noneal and superoxide anion radical production and increased expressions
Reduced the increased 4-hydroxy-2-noneal and superoxide anion radical production and increased expressions
Reduced the increased 4-hydroxy-2-noneal and superoxide anion radical production and increased expressions
Attenuated ischemia-induced memory deficits
Increased BDNF levels and attenuated hippocampal CA1 neurodegeneration and
Reduced brain infarct volume) and lessened the extent of histological damage
Decrease in hippocampal neurodegeneration
Prevented the cognitive and emotional impairments
Decreases hippocampal neuroinflammation by downregulated Iba-1 (microglia) and GFAP (astrocytes)
Decreases hippocampal neuroinflammation by downregulated Iba-1 (microglia) and GFAP (astrocytes)
Inrease in the hippocampal brain derived neurotrophic factor (BDNF) protein levels
Reduced the infarction area, neurological deficit score
Reduced protein expression of iNOS and COX-2, and attenuated inhibition of p53 protein
Reduced protein expression of iNOS and COX-2, and attenuated inhibition of p53 protein
Reduced protein expression of iNOS and COX-2, and attenuated inhibition of p53 protein
Reduced protein expression of iNOS and COX-2, and attenuated inhibition of p53 protein
Reduced protein expression of iNOS and COX-2, and attenuated inhibition of p53 protein
Reduced protein expression of iNOS and COX-2, and attenuated inhibition of p53 protein
Reduced mRNA expression of iNOS, COX-2, and TNFα.
Reduced mRNA expression of iNOS, COX-2, and TNFα.
Reduced mRNA expression of iNOS, COX-2, and TNFα.

Target	Protein level
Caspase-3	Decrease protein leves
Caspase-3	-
BDNF	Increase Preotein levels
BDNF	-
CaMKII	Decrease protein leves
GABA(A)R α1	Increase Preotein levels
GABA(A)R γ2	Increase Preotein levels
KCC2	Increase Preotein levels
GABA(A)R α1	-
GABA(A)R γ2	-
KCC2	-
NKCC1	Decrease protein leves
NKCC1	-
PI3K	Increase Preotein levels
Akt	Increase Preotein levels
BDNF	Increase Preotein levels
BCL-2	Increase Preotein levels
MCL-1	Increase Preotein levels
MCL-1	-
MCL-1	-
IL-6	Decrease protein leves
IL-18	Decrease protein leves
TNF-α	Decrease protein leves
BCL-2	Increase Preotein levels
Bax	Decrease protein leves
Caspase-3	Decrease protein leves
PI3K	Increase Preotein levels
Akt	Increase Preotein levels
mTOR	Increase Preotein levels
MAP-2	Increase Preotein levels
BDNF	Increase Preotein levels
NT-3	Increase Preotein levels
IGF-1	Increase Preotein levels
iNOS	Decrease protein leves
TNF-α	Decrease protein leves
IL-1β	Decrease protein leves
p-JNK	Decrease protein leves
p-p38	Decrease protein leves
NO	Decrease protein leves
TNF-α	Decrease protein leves
IL-1β	Decrease protein leves
ROS	Decrease protein leves
NO	-
TNF-α	-
IL-1β	-
ROS	-

Glu	Decrease protein leves
Asp	Decrease protein leves
Gly	Decrease protein leves
GABA	Decrease protein leves
Tau	Decrease protein leves
LDH	Decrease protein leves
Caspase-3	Decrease protein leves
iNOS	Decrease protein leves
-	-
Ki-67	Increase Preotein levels
BCL-2	Increase Preotein levels
Caspase-3	Increase Preotein levels
Bax	Increase Preotein levels
IL-10	Increase Preotein levels
IL-6	Decrease protein leves
TNF-α	Decrease protein leves
Ulk1	Increase Preotein levels
mTOR	Increase Preotein levels
PPAR-γ	Increase Preotein levels
Caspase-3	-
BCL-2	-
Caveolin-1-KD	-
VEGFR2	Increase Preotein levels
CD34	Increase Preotein levels
H3	-
H4	-
Syn-I	-
HDAC	Decrease protein leves
BDNF	Increase Preotein levels
IL-1β	Decrease protein leves
TGF-β1	Decrease protein leves
-	-
IL-1β	Decrease protein leves
TGF-β1	Decrease protein leves
PPARα	Increase Preotein levels
PPARγ	Increase Preotein levels
IL-1β	Decrease protein leves
IL-6	Decrease protein leves
TNF-α	Decrease protein leves
XBP1u	Decrease protein leves
Caspase-3	Decrease protein leves
XBP1s	Decrease protein leves
IL-1β	-
IL-6	-
TNF-α	-
XBP1u	-

Caspase-3	-
XBP1s	-
p-IRE1α	Decrease protein leves
p-IRE1α	-
-	-
TNF-α	Decrease protein leves
IL-6	Decrease protein leves
Caspase-3	Decrease protein leves
Bax	Decrease protein leves
TNF-α	-
IL-6	-
Caspase-3	-
Bax	-
BCL-2	Increase Preotein levels
-	-
Caspase-3	Decrease protein leves
Caspase-3	-
-	-
ROS	Decrease protein leves
MDA	Decrease protein leves
Caspase-3	Decrease protein leves
BCL-2	Increase Preotein levels
-	-
4-HNE	Decrease protein leves
BDNF	Increase Preotein levels
TrkB	Increase Preotein levels
-	-
p62	-
NBR1	-
BCL-2	-
TNF-α	Decrease protein leves
-	-
-	-
Mfn-2	Decrease protein leves
BCL-2	Decrease protein leves
Apaf-1	Decrease protein leves
-	-
TNF-α	Decrease protein leves
IL-1β	Decrease protein leves
IL-6	Decrease protein leves
ACSL4	Decrease protein leves
TFR1	Decrease protein leves
PGC-1α	-
TFAM	-
Caspase-3	Decrease protein leves
PI3K	-

p55γ	-
-	-
TNF-α	Decrease protein leves
IL-6	Decrease protein leves
IL-1β	Increase protein leves
CD68	Decrease protein leves
Akt	Increase protein leves
ERK	Increase protein leves
-	-
nNOS	Decrease protein leves
NO	Increase protein leves
p-Akt	Increase protein leves
Akt	Increase protein leves
p-eNOS	Increase protein leves
eNOS	Increase protein leves
BCL-2	Increase protein leves
BCL-2	-
Bax	Decrease protein leves
Bax	-
BCL-2	Increase protein leves
Bax	Decrease protein leves
Caspase-3	Decrease protein leves
PARP	Decrease protein leves
-	-
MMP-2	Decrease protein leves
MMP-9	Decrease protein leves
Nrf2	Increase protein leves
TLR4	Decrease protein leves
p-p38	Decrease protein leves
IL-1	Decrease protein leves
p-Akt	Increase protein leves
p-mTOR	Increase protein leves
-	-
IL-1β	Decrease protein leves
IL-8	Decrease protein leves
p-JAK2	Increase protein leves
p-STAT3	Increase protein leves
Flotillin-1	Increase protein leves
LDH	Increase protein leves
Caspase-3	Increase protein leves
-	-
BCL-2	Increase protein leves
Caspase-3	Decrease protein leves
Caspase-3	Decrease protein leves
LC3 II	Decrease protein leves
HIF-1α	Decrease protein leves

-	-
NRF2	Increase protein leves
TFAM	Increase protein leves
NRF2	-
TFAM	-
CD11b	-
NF-κB	Decrease protein leves
JNK	Decrease protein leves
iNOS	Decrease protein leves
COX-2	Decrease protein leves
-	-
MMP-9	Decrease protein leves
TIMP-1	Increase protein leves
-	-
Sirt1	Increase protein leves
Caspase-3	Decrease protein leves
IL-1β	Decrease protein leves
IL-1β	Decrease protein leves
IL-1β	-
IL-6	-
TNF-α	-
p38	Decrease protein leves
MAPK	Decrease protein leves
Iba1	Decrease protein leves
Bax	Increase protein leves
BCL-2	Decrease protein leves
NR2B	Decrease protein leves
-	-
IL-1β	-
IL-6	-
TNF-α	-
IFN-γ	-
IL-10	-
TGFβ	-
IL-1β	Decrease protein leves
IL-6	Decrease protein leves
IL-2	Decrease protein leves
IL-8	Decrease protein leves
TNF-α	Decrease protein leves
LDH	Decrease protein leves
TNF-α	Decrease protein leves
IL-6	Decrease protein leves
IL-8	Decrease protein leves
NF-κB	Decrease protein leves
IL-6	Decrease protein leves
TNF-α	Decrease protein leves

NeuN	Increase protein leves
NLRP3	Decrease protein leves
ASC	Decrease protein leves
Caspase-3	Decrease protein leves
IL-1β	Decrease protein leves
p62	Decrease protein leves
-	-
-	-
SOD	Increase protein leves
MDA	Decrease protein leves
ET-1	Decrease protein leves
TNF-α	Decrease protein leves
NF-κB	Decrease protein leves
TLR4	Decrease protein leves
Caspase-3	Decrease protein leves
p-MEK1	Decrease protein leves
p-MEK2	Decrease protein leves
p-ERK1	Decrease protein leves
p-ERK2	Decrease protein leves
COX-2	Decrease protein leves
p-MEK1	-
p-MEK2	-
p-ERK1	-
p-ERK2	-
COX-2	-
-	-
Caspase-3	Decrease protein leves
Caspase-3	Decrease protein leves
Caspase-3	Decrease protein leves
p-ERK1	Increase protein leves
p-ERK2	Increase protein leves
p-JNK	Increase protein leves
IL-1β	Decrease protein leves
MDA	Decrease protein leves
SOD	Increase protein leves
Survivin	Increase protein leves
-	-
ICAM-1	Decrease protein leves
TLR4	Decrease protein leves
Fas	-
FasL	-
Caspase-8	-
Bax	-
BCL-2	-
Caspase-8	Increase protein leves
Bid	Increase protein leves

Caspase-3	Increase protein leves
Cyto C	Increase protein leves
-	-
NLRP3	Decrease protein leves
Caspase-1	Decrease protein leves
IL-1β	Decrease protein leves
GSDMD	Decrease protein leves
GSDMD-N	Decrease protein leves
-	-
Caspase-3	Decrease protein leves
BCL-2	Decrease protein leves
Bax	Decrease protein leves
NOX2	Decrease protein leves
iNOS	Decrease protein leves
Caspase-3	-
NRF2	Increase protein leves
GFAP	Decrease protein leves
NeuN	Increase protein leves
IL-1β	Decrease protein leves
IL-6	Decrease protein leves
TNF-α	Decrease protein leves
-	-
IL-6	Decrease protein leves
IL-1β	Decrease protein leves
TNF-α	Decrease protein leves
SOD	Increase protein leves
-	-
HO-1	Increase protein leves
Nqo1	Increase protein leves
SOD	Increase protein leves
p-Akt	Increase protein leves
p-Nrf2	Increase protein leves
Nrf2	Increase protein leves
-	-
Bax	Decrease protein leves
BCL-2	Decrease protein leves
-	-
TNF-α	Decrease protein leves
IL-1β	Decrease protein leves
LDH	Decrease protein leves
-	-
Bax	Decrease protein leves
BCL-2	Decrease protein leves
ROS	Decrease protein leves
NO	Decrease protein leves
iNOS	Decrease protein leves

TNF-α	Decrease protein leves
VEGF	Increase protein leves
TNF-α	Decrease protein leves
-	-
Bax	Decrease protein leves
BCL-2	Decrease protein leves
Bax	-
BCL-2	-
Apaf-1	Decrease protein leves
Bad	Decrease protein leves
Caspase-3	Decrease protein leves
BCL-2	Increase protein leves
Apaf-1	-
Bad	-
Caspase-3	-
BCL-2	-
-	-
Caspase-3	Decrease protein leves
BDNF	Increase protein leves
CREB	Increase protein leves
BCL-2	Decrease protein leves
BCL-2	Increase protein leves
Bax	Decrease protein leves
-	-
ROS	-
GLT-1	Increase protein leves
BCL-2	Increase protein leves
-	-
Glu	Decrease protein leves
Asp	Decrease protein leves
MDA	Decrease protein leves
MMP-3	Decrease protein leves
-	-
-	-
CD16	-
Iba1	-
TNF-α	-
IL-1β	-
CCL3	-
-	-
IL-1β	Decrease protein leves
IL-6	Decrease protein leves
MPO	Decrease protein leves
TNF-α	Decrease protein leves
MDA	Decrease protein leves
SOD	Decrease protein leves

p53	Decrease protein leves
Bax	Decrease protein leves
BCL-2	Decrease protein leves
Dihydroethidium	Decrease protein leves
8-hydroxyguanine	Decrease protein leves
4-hydroxy-2-nonenal	Decrease protein leves
SOD1	Decrease protein leves
SOD2	Decrease protein leves
GFAP	Decrease protein leves
4-hydroxy-2-noneal	Decrease protein leves
Superoxide	Decrease protein leves
SOD1	Increase protein leves
SOD2	Increase protein leves
-	-
BDNF	Increase protein leves
-	-
-	-
-	-
Iba1	Decrease protein leves
GFAP	Decrease protein leves
BDNF	Increase protein leves
-	-
iNOS	Decrease protein leves
COX-2	Decrease protein leves
p53	Increase protein leves
iNOS	-
COX-2	-
p53	-
iNOS	Decrease protein leves
COX-2	Decrease protein leves
TNF-α	Decrease protein leves

Method	RNA expression
Western blot	-
-	Downregulated RNA expression
Western blot	-
-	Upregulated RNA expression
Western blotting	-
Western blot	-
Western blot	-
Western blot	-
-	Upregulated RNA expression
-	Upregulated RNA expression
-	Upregulated RNA expression
Western blot	-
-	Downregulated RNA expression
Western blot	-
Western blot	-
ELISA	-
Western blot	-
Western blot	-
-	Upregulated RNA expression
-	Upregulated RNA expression
ELISA	-
ELISA	-
ELISA	-
Western blot	-
Western blot	-
Western blot	-
Western blotting	-
Western blotting	-
Western blotting	-
Western blotting	-
Western blotting	-
Western blotting	-
Western blotting	-
Western blot	-
Western blot	-
Western blot	-
Western blot / Immunofuorescence	-
Western blot / Immunofuorescence	-
Western blotting	-
Western blotting	-
Western blotting	-
Western blotting	-
-	Downregulated RNA expression
-	Downregulated RNA expression
-	Downregulated RNA expression
-	Downregulated RNA expression

UPLC–MS/MS MRM chromatograms	-
UPLC–MS/MS MRM chromatograms	-
UPLC–MS/MS MRM chromatograms	-
UPLC–MS/MS MRM chromatograms	-
UPLC–MS/MS MRM chromatograms	-
Western blot	-
Western blot	-
ELISA	-
-	-
Western blot / Immunochemistry	-
Western blot / Immunochemistry	-
Western blot / Immunochemistry	-
Western blot / Immunochemistry	-
ELISA	-
ELISA	-
ELISA	-
Western blot / Immunofuorescence	-
Western blot / Immunofuorescence	-
Western blot / Immunofuorescence	-
-	Upregulated RNA expression
-	Downregulated RNA expression
-	Downregulated RNA expression
Immunofluorescence	-
Immunofluorescence	-
-	Upregulated RNA expression
-	Upregulated RNA expression
-	Upregulated RNA expression
ELISA	-
Western blot	-
Western blot	-
Western blot	-
-	-
Western blot	-
Western blot	-
Western blot	-
Western blot	-
ELISA	-
ELISA	-
ELISA	-
ELISA	-
ELISA	-
ELISA	-
-	Downregulated RNA expression
-	Downregulated RNA expression
-	Downregulated RNA expression
-	Downregulated RNA expression

-	Downregulated RNA expression
-	Downregulated RNA expression
Western blot	-
-	Downregulated RNA expression
-	-
Western blot	-
Western blot	-
Western blot	-
Western blot	-
-	Downregulated RNA expression
-	Downregulated RNA expression
-	Downregulated RNA expression
-	Downregulated RNA expression
Western blot	Upregulated RNA expression
-	-
Western blot	-
-	Downregulated RNA expression
-	-
Western blot	-
Western blot	-
Western blot	-
Western blot	-
-	-
Immunochemistry	-
Western Blotting / ELISA	-
Western Blotting / ELISA	-
-	-
-	Upregulated RNA expression
-	Upregulated RNA expression
-	Upregulated RNA expression
Western blotting / Inmunohistochemistry	-
-	-
-	-
Western Blot	-
Western Blot	-
Western Blot	-
-	-
ELISA	-
ELISA	-
ELISA	-
Western blotting	-
Western blotting	-
-	Upregulated RNA expression
-	Upregulated RNA expression
Western Blot	-
-	Upregulated RNA expression

-	Upregulated RNA expression
-	-
Immunohistochemistry	-
Immunohistochemistry	-
Immunohistochemistry	-
Immunohistochemistry	-
Immunohistochemistry	-
Immunohistochemistry	-
-	-
Immunohistochemistry	-
Western blot	-
Western blot	-
Western blot	-
Western blot	-
Western blot	-
Western Blot / immunochemistry	-
-	Upregulated RNA expression
Western Blot / immunochemistry	-
-	Downregulated RNA expression
Western Blot / immunochemistry	-
Western Blot / immunochemistry	-
Western Blot / immunochemistry	-
Western Blot / immunochemistry	-
-	-
Western blot / confocal microscopy	-
Western blot / confocal microscopy	-
Western blotting	-
Western blot	-
Western blot	-
Western blot	-
Western blot	-
Western blot	-
-	-
Western blot	-
Western blot	-
Western blot	-
Western blot	-
Western blot	-
Western blot	-
Western blot	-
-	-
Western blotting	-
Western blotting	-
Western blotting	-
Western blot	-
Western blot	-

-	-
Western blotting / immunochemistry	-
Western blotting / immunochemistry	-
-	Upregulated RNA expression
-	Upregulated RNA expression
-	-
Western blot	-
Western blot	-
Western blot	-
Western blot	-
-	-
Western blot	-
Western blot	-
-	-
Western blot	-
Western blot	-
Western blot	-
Western blot	-
-	Downregulated RNA expression
-	Downregulated RNA expression
-	Downregulated RNA expression
Western blot	-
Western blot	-
Western blot	-
Western blot	-
Western blot	-
Western blot	-
-	-
-	Downregulated RNA expression
-	Downregulated RNA expression
-	Downregulated RNA expression
-	Downregulated RNA expression
-	Upregulated RNA expression
-	Upregulated RNA expression
ELISA	-
ELISA	-
ELISA	-
ELISA	-
ELISA	-
LDH cytotoxicity detection kit	-
ELISA	-
ELISA	-
ELISA	-
Western blot	-
Western blot	-
Western blot	-

Western blot	-
Western blotting / ELISA	-
Western blotting / ELISA	-
Western blotting / ELISA	-
Western blotting / ELISA	-
Western blotting / ELISA	-
-	-
-	-
Radioimmunoassay	-
Radioimmunoassay	-
Radioimmunoassay	-
ELISA	-
ELISA	-
ELISA	-
ELISA	-
Western blot	-
Western blot	-
Western blot	-
Western blot	-
Western blot	-
-	Downregulated RNA expression
-	Downregulated RNA expression
-	Downregulated RNA expression
-	Downregulated RNA expression
-	Downregulated RNA expression
-	-
Western blot	-
Western blot	-
Western blot	-
Western blot	-
Western blot	-
Western blot	-
Western blot	-
Western blot	-
Western blot	-
Western blot	-
-	-
Western blot	-
Western blot	-
-	Downregulated RNA expression
-	Downregulated RNA expression
-	Downregulated RNA expression
-	Downregulated RNA expression
-	Downregulated RNA expression
Western blot / Immunohistochemistry	-
Western blot / Immunohistochemistry	-

Western blot / Immunohistochemistry	-
Western blot / Immunohistochemistry	-
-	-
Western blot	-
Western blot	-
Western blot	-
Western blot	-
Western blot	-
-	-
Western blot	-
Western blot	-
Western blot	-
Western blot	-
Western blot	-
-	-
Western blotting	-
Western blot	-
Western blot	-
ELISA	-
ELISA	-
ELISA	-
-	-
ELISA	-
ELISA	-
ELISA	-
Western blot	-
-	-
Western blot	-
Western blot	-
Western blot	-
Western blot	-
Western blot	-
Western blot	-
-	-
Western blot	-
Western blot	-
-	-
Western blot / ELISA	-
Western blot / ELISA	-
Western blot / ELISA	-
-	-
Western blotting	-
Western blotting	-
Western blot	-
Western blot	-
Western blot	-

Western blot	-
ELISA	-
ELISA	-
-	-
Immunohistochemistry	-
Immunohistochemistry	-
-	Downregulated RNA expression
-	Downregulated RNA expression
Western blot	-
Western blot	-
Western blot	-
Western blot	-
-	Downregulated RNA expression
-	Downregulated RNA expression
-	Downregulated RNA expression
-	Upregulated RNA expression
-	-
Western blot	-
Western blot	-
Western blot	-
Western blot	-
Western blot	-
Western blot	-
-	-
-	-
Western blotting	-
Western blotting	-
-	-
HPLC	-
HPLC	-
Western blot	-
Western blot	-
-	-
-	-
-	Downregulated RNA expression
-	Downregulated RNA expression
-	Downregulated RNA expression
-	Downregulated RNA expression
-	Downregulated RNA expression
-	-
ELISA	-
ELISA	-
ELISA	-
ELISA	-
ELISA	-
ELISA	-

ELISA	-
ELISA	-
ELISA	-
Western blot	-
Western blot	-
Western blot	-
Western blot	-
Western blot	-
Immunohistochemistry	-
Western blot	-
Western blot	-
Western blot	-
Western blot	-
-	-
Western blot / Immunohistochemistry	-
-	-
-	-
-	-
Western blot / Immunohistochemistry	-
Western blot / Immunohistochemistry	-
Western blot / Immunohistochemistry	-
-	-
Western blot	-
Western blot	-
Western blot	-
-	Downregulated RNA expression
-	Downregulated RNA expression
-	Downregulated RNA expression
Western blot	-
Western blot	-
Western blot	-

Method	Year	PMID
-	2011	21600966
RT-PCR	2011	21600966
-	2011	21600966
RT-PCR	2011	21600966
-	2016	27016057
-	2013	23041106
-	2013	23041106
-	2013	23041106
RT-PCR	2013	23041106
RT-PCR	2013	23041106
RT-PCR	2013	23041106
-	2013	23041106
RT-PCR	2013	23041106
-	2021	34234667
-	2021	34234667
-	2021	34234667
-	2015	26485504
-	2015	26485504
RT-PCR	2015	26485504
RT-PCR	2015	26485504
-	2019	31228802
-	2019	31228802
-	2019	31228802
-	2019	31228802
-	2019	31228802
-	2019	31228802
-	2019	31228802
-	2019	31228802
-	2019	31228802
-	2016	27105682
-	2016	27105682
-	2016	27105682
-	2016	27105682
-	2020	31792810
-	2020	31792810
-	2020	31792810
-	2020	31792810
-	2020	31792810
-	2011	21524691
-	2011	21524691
-	2011	21524691
-	2011	21524691
RT-PCR	2011	21524691
RT-PCR	2011	21524691
RT-PCR	2011	21524691
RT-PCR	2011	21524691

-	2014	24423938
-	2014	24423938
-	2014	24423938
-	2014	24423938
-	2014	24423938
-	2019	30876978
-	2019	30876978
-	2019	30876978
-	2018	29710456
-	2018	29710456
-	2018	29710456
-	2018	29710456
-	2018	29710456
-	2018	29710456
-	2018	29710456
-	2018	29710456
-	2018	29710456
-	2018	29710456
-	2018	29710456
RT-PCR	2018	30622670
RT-PCR	2018	30622670
RT-PCR	2018	30622670
-	2018	30622670
-	2018	30622670
RT-PCR	2017	28821706
RT-PCR	2017	28821706
RT-PCR	2017	28821706
-	2017	28821706
-	2017	28821706
-	2016	26939761
-	2016	26939761
-	2016	26939761
-	2015	26679678
-	2015	26679678
-	2015	26679678
-	2015	26679678
-	2020	32470450
-	2020	32470450
-	2020	32470450
-	2020	32470450
-	2020	32470450
-	2020	32470450
qRT-PCR	2020	32470450
qRT-PCR	2020	32470450
qRT-PCR	2020	32470450
qRT-PCR	2020	32470450

qRT-PCR	2020	32470450
qRT-PCR	2020	32470450
-	2020	32470450
qRT-PCR	2020	32470450
-	2021	33978900
-	2021	33978900
-	2021	33978900
-	2021	33978900
-	2021	33978900
q-PCR	2021	33978900
q-PCR	2021	33978900
q-PCR	2021	33978900
q-PCR	2021	33978900
q-PCR	2021	33978900
-	2020	31982469
-	2020	31982469
RT-PCR	2020	31982469
-	2018	29454613
-	2018	29454613
-	2018	29454613
-	2018	29454613
-	2018	29454613
-	2012	23123262
-	2012	23123262
-	2020	31927682
-	2020	31927682
-	2018	29402799
RT-PCR	2018	29402799
RT-PCR	2018	29402799
RT-PCR	2018	29402799
-	2018	29402799
-	2020	32108303
-	2020	31816340
-	2020	31816340
-	2020	31816340
-	2020	31816340
-	2021	33576458
-	2021	33576458
-	2021	33576458
-	2021	33576458
-	2021	33576458
-	2021	33576458
q-PCR	2016	26687185
q-PCR	2016	26687185
-	2011	22119079
RT-PCR / sequenciation	2011	22119079

RT-PCR / sequenciation	2011	22119079
-	2018	28495517
-	2018	28495517
-	2018	28495517
-	2018	28495517
-	2015	26043690
-	2015	26043690
-	2015	26043690
-	2010	20501058
-	2010	20501058
-	2019	31250662
-	2019	31250662
-	2019	31250662
-	2019	31250662
-	2019	31250662
-	2010	20387227
RT-PCR / sequenciation	2010	20387227
-	2010	20387227
RT-PCR / sequenciation	2010	20387227
-	2018	29974381
-	2018	29974381
-	2018	29974381
-	2018	29974381
-	2015	26059793
-	2015	26059793
-	2015	26059793
-	2019	30414381
-	2018	29243061
-	2018	29243061
-	2018	29243061
-	2018	29243061
-	2018	29243061
-	2015	26628981
-	2015	26628981
-	2015	26628981
-	2015	26628981
-	2015	26628981
-	2018	29353037
-	2018	29353037
-	2018	29353037
-	2018	29484272
-	2018	29484272
-	2018	29484272
-	2018	29484272
-	2019	31040648
-	2019	31040648

-	2018	29777940
-	2014	24777807
-	2014	24777807
RT-PCR	2014	24777807
RT-PCR	2014	24777807
-	2012	22709670
-	2012	22709670
-	2012	22709670
-	2012	22709670
-	2012	22709670
-	2014	25330860
-	2014	25330860
-	2014	25330860
-	2017	28683365
-	2017	28683365
-	2017	28683365
-	2017	28683365
-	2017	28683365
RT-PCR	2014	24631487
RT-PCR	2014	24631487
RT-PCR	2014	24631487
-	2014	24631487
-	2014	24631487
-	2014	24631487
-	2014	25221579
-	2014	25221579
-	2014	25221579
-	2018	29426336
RT-PCR	2018	29426336
RT-PCR	2018	29426336
RT-PCR	2018	29426336
RT-PCR	2018	29426336
RT-PCR	2018	29426336
RT-PCR	2018	29426336
-	2018	29426336
-	2018	29426336
-	2018	29426336
-	2018	29426336
-	2018	29426336
-	2021	34988203
-	2021	34988203
-	2021	34988203
-	2021	34988203
-	2019	31230155
-	2019	31230155
-	2019	31230155

-	2019	31230155
-	2020	32361652
-	2020	32361652
-	2020	32361652
-	2020	32361652
-	2020	32361652
-	2017	28957809
-	2021	34602413
-	2014	24720795
-	2014	24720795
-	2014	24720795
-	2010	20618938
-	2010	20618938
-	2010	20618938
-	2010	21151457
-	2015	26240040
-	2015	26240040
-	2015	26240040
-	2015	26240040
-	2015	26240040
RT-PCR	2015	26240040
RT-PCR	2015	26240040
RT-PCR	2015	26240040
RT-PCR	2015	26240040
RT-PCR	2015	26240040
-	2017	28054226
-	2017	28054226
-	2017	28054226
-	2017	28054226
-	2013	23646480
-	2013	23646480
-	2013	23646480
-	2013	24517072
-	2019	31412899
-	2019	31412899
-	2019	31412899
-	2012	22342823
-	2012	22342823
-	2017	28011393
RT-PCR	2019	31639488
RT-PCR	2019	31639488
RT-PCR	2019	31639488
RT-PCR	2019	31639488
RT-PCR	2019	31639488
-	2019	31639488
-	2019	31639488

-	2019	31639488
-	2019	31639488
-	2021	35003524
-	2021	35003524
-	2021	35003524
-	2021	35003524
-	2021	35003524
-	2021	35003524
-	2014	24189392
-	2014	24189392
-	2014	24189392
-	2014	24189392
-	2011	21512969
-	2011	21512969
-	2013	23930775
-	2016	26746802
-	2021	34368862
-	2021	34368862
-	2021	34368862
-	2021	34368862
-	2021	34368862
-	2010	20845788
-	2010	20845788
-	2010	20845788
-	2010	20845788
-	2019	30815818
-	2019	30815818
-	2019	30815818
-	2019	30815818
-	2019	30815818
-	2019	30815818
-	2019	30815818
-	2019	30815818
-	2018	29542683
-	2018	29542683
-	2018	29542683
-	2019	31678414
-	2019	31678414
-	2019	31678414
-	2019	31678414
-	2021	34273708
-	2021	34273708
-	2021	34273708
-	2011	21168474
-	2011	21168474
-	2011	21168474

-	2011	21168474
-	2018	29728011
-	2018	29728011
-	2018	29728011
-	2018	29728011
-	2018	29728011
RT-PCR	2018	29728011
RT-PCR	2018	29728011
-	2021	33793797
-	2021	33793797
-	2021	33793797
-	2021	33793797
RT-PCR	2021	33793797
RT-PCR	2021	33793797
RT-PCR	2021	33793797
RT-PCR	2021	33793797
-	2022	35095491
-	2022	35095491
-	2022	35095491
-	2022	35095491
-	2016	27151150
-	2016	27151150
-	2016	27151150
-	2020	32326191
-	2020	32326191
-	2020	32326191
-	2020	32326191
-	2011	21554870
-	2011	21554870
-	2011	21554870
-	2011	21554870
-	2011	21554870
-	2019	30978320
-	2021	34130727
RT-PCR	2021	34130727
RT-PCR	2021	34130727
RT-PCR	2021	34130727
RT-PCR	2021	34130727
RT-PCR	2021	34130727
-	2017	27871155
-	2017	27871155
-	2017	27871155
-	2017	27871155
-	2017	27871155
-	2017	27871155
-	2017	27871155

-	2017	27871155
-	2017	27871155
-	2017	27871155
-	2019	30696078
-	2019	30696078
-	2019	30696078
-	2019	30696078
-	2019	30696078
-	2019	30551426
-	2019	30551426
-	2019	30551426
-	2019	30551426
-	2019	30551426
-	2021	34302281
-	2021	34302281
-	2012	22659086
-	2014	24532152
-	2016	27889412
-	2016	27889412
-	2016	27889412
-	2016	27889412
-	2016	27087648
-	2016	27087648
-	2016	27087648
-	2016	27087648
RT-PCR	2016	27087648
RT-PCR	2016	27087648
RT-PCR	2016	27087648
-	2016	27087648
-	2016	27087648
-	2016	27087648

Reference

Cao Y, Mao X, Sun C, Zheng P, Gao J, Wang X, Min D, Sun H, Xie N, Cai J. Baicalin attenuates global cerebral isch
Cao Y, Mao X, Sun C, Zheng P, Gao J, Wang X, Min D, Sun H, Xie N, Cai J. Baicalin attenuates global cerebral isch
Cao Y, Mao X, Sun C, Zheng P, Gao J, Wang X, Min D, Sun H, Xie N, Cai J. Baicalin attenuates global cerebral isch
Cao Y, Mao X, Sun C, Zheng P, Gao J, Wang X, Min D, Sun H, Xie N, Cai J. Baicalin attenuates global cerebral isch
Wang P, Cao Y, Yu J, Liu R, Bai B, Qi H, Zhang Q, Guo W, Zhu H, Qu L. Baicalin alleviates ischemia-induced memo
Dai J, Chen L, Qiu YM, Li SQ, Xiong WH, Yin YH, Jia F, Jiang JY. Activations of GABAergic signaling, HSP70 and MA
Dai J, Chen L, Qiu YM, Li SQ, Xiong WH, Yin YH, Jia F, Jiang JY. Activations of GABAergic signaling, HSP70 and MA
Dai J, Chen L, Qiu YM, Li SQ, Xiong WH, Yin YH, Jia F, Jiang JY. Activations of GABAergic signaling, HSP70 and MA
Dai J, Chen L, Qiu YM, Li SQ, Xiong WH, Yin YH, Jia F, Jiang JY. Activations of GABAergic signaling, HSP70 and MA
Dai J, Chen L, Qiu YM, Li SQ, Xiong WH, Yin YH, Jia F, Jiang JY. Activations of GABAergic signaling, HSP70 and MA
Dai J, Chen L, Qiu YM, Li SQ, Xiong WH, Yin YH, Jia F, Jiang JY. Activations of GABAergic signaling, HSP70 and MA
Dai J, Chen L, Qiu YM, Li SQ, Xiong WH, Yin YH, Jia F, Jiang JY. Activations of GABAergic signaling, HSP70 and MA
Dai J, Chen L, Qiu YM, Li SQ, Xiong WH, Yin YH, Jia F, Jiang JY. Activations of GABAergic signaling, HSP70 and MA
Li C, Sui C, Wang W, Yan J, Deng N, Du X, Cheng F, Ma X, Wang X, Wang Q. Baicalin Attenuates Oxygen-Glucose
Li C, Sui C, Wang W, Yan J, Deng N, Du X, Cheng F, Ma X, Wang X, Wang Q. Baicalin Attenuates Oxygen-Glucose
Li C, Sui C, Wang W, Yan J, Deng N, Du X, Cheng F, Ma X, Wang X, Wang Q. Baicalin Attenuates Oxygen-Glucose
Zheng WX, Cao XL, Wang F, Wang J, Ying TZ, Xiao W, Zhang Y, Xing H, Dong W, Xu SQ, Min ZL, Wu FJ, Hu XM. Ba
Zheng WX, Cao XL, Wang F, Wang J, Ying TZ, Xiao W, Zhang Y, Xing H, Dong W, Xu SQ, Min ZL, Wu FJ, Hu XM. Ba
Zheng WX, Cao XL, Wang F, Wang J, Ying TZ, Xiao W, Zhang Y, Xing H, Dong W, Xu SQ, Min ZL, Wu FJ, Hu XM. Ba
Zheng WX, Cao XL, Wang F, Wang J, Ying TZ, Xiao W, Zhang Y, Xing H, Dong W, Xu SQ, Min ZL, Wu FJ, Hu XM. Ba
Yang S, Wang H, Yang Y, Wang R, Wang Y, Wu C, Du G. Baicalein administered in the subacute phase ameliorat
Yang S, Wang H, Yang Y, Wang R, Wang Y, Wu C, Du G. Baicalein administered in the subacute phase ameliorat
Yang S, Wang H, Yang Y, Wang R, Wang Y, Wu C, Du G. Baicalein administered in the subacute phase ameliorat
Yang S, Wang H, Yang Y, Wang R, Wang Y, Wu C, Du G. Baicalein administered in the subacute phase ameliorat
Yang S, Wang H, Yang Y, Wang R, Wang Y, Wu C, Du G. Baicalein administered in the subacute phase ameliorat
Yang S, Wang H, Yang Y, Wang R, Wang Y, Wu C, Du G. Baicalein administered in the subacute phase ameliorat
Yang S, Wang H, Yang Y, Wang R, Wang Y, Wu C, Du G. Baicalein administered in the subacute phase ameliorat
Yang S, Wang H, Yang Y, Wang R, Wang Y, Wu C, Du G. Baicalein administered in the subacute phase ameliorat
Yang S, Wang H, Yang Y, Wang R, Wang Y, Wu C, Du G. Baicalein administered in the subacute phase ameliorat
Fang M, Yuan Y, Lu J, Li HE, Zhao M, Ling EA, Wu CY. Scutellarin promotes microglia-mediated astrogliosis coup
Fang M, Yuan Y, Lu J, Li HE, Zhao M, Ling EA, Wu CY. Scutellarin promotes microglia-mediated astrogliosis coup
Fang M, Yuan Y, Lu J, Li HE, Zhao M, Ling EA, Wu CY. Scutellarin promotes microglia-mediated astrogliosis coup
Fang M, Yuan Y, Lu J, Li HE, Zhao M, Ling EA, Wu CY. Scutellarin promotes microglia-mediated astrogliosis coup
Chen HL, Jia WJ, Li HE, Han H, Li F, Zhang XL, Li JJ, Yuan Y, Wu CY. Scutellarin Exerts Anti-Inflammatory Effects ir
Chen HL, Jia WJ, Li HE, Han H, Li F, Zhang XL, Li JJ, Yuan Y, Wu CY. Scutellarin Exerts Anti-Inflammatory Effects ir
Chen HL, Jia WJ, Li HE, Han H, Li F, Zhang XL, Li JJ, Yuan Y, Wu CY. Scutellarin Exerts Anti-Inflammatory Effects ir
Chen HL, Jia WJ, Li HE, Han H, Li F, Zhang XL, Li JJ, Yuan Y, Wu CY. Scutellarin Exerts Anti-Inflammatory Effects ir
Chen HL, Jia WJ, Li HE, Han H, Li F, Zhang XL, Li JJ, Yuan Y, Wu CY. Scutellarin Exerts Anti-Inflammatory Effects ir
Wang S, Wang H, Guo H, Kang L, Gao X, Hu L. Neuroprotection of Scutellarin is mediated by inhibition of micro
Wang S, Wang H, Guo H, Kang L, Gao X, Hu L. Neuroprotection of Scutellarin is mediated by inhibition of micro
Wang S, Wang H, Guo H, Kang L, Gao X, Hu L. Neuroprotection of Scutellarin is mediated by inhibition of micro
Wang S, Wang H, Guo H, Kang L, Gao X, Hu L. Neuroprotection of Scutellarin is mediated by inhibition of micro
Wang S, Wang H, Guo H, Kang L, Gao X, Hu L. Neuroprotection of Scutellarin is mediated by inhibition of micro
Wang S, Wang H, Guo H, Kang L, Gao X, Hu L. Neuroprotection of Scutellarin is mediated by inhibition of micro
Wang S, Wang H, Guo H, Kang L, Gao X, Hu L. Neuroprotection of Scutellarin is mediated by inhibition of micro
Wang S, Wang H, Guo H, Kang L, Gao X, Hu L. Neuroprotection of Scutellarin is mediated by inhibition of micro

Tang H, Tang Y, Li N, Shi Q, Guo J, Shang E, Duan JA. Neuroprotective effects of scutellarin and scutellarein on r
Tang H, Tang Y, Li N, Shi Q, Guo J, Shang E, Duan JA. Neuroprotective effects of scutellarin and scutellarein on r
Tang H, Tang Y, Li N, Shi Q, Guo J, Shang E, Duan JA. Neuroprotective effects of scutellarin and scutellarein on r
Tang H, Tang Y, Li N, Shi Q, Guo J, Shang E, Duan JA. Neuroprotective effects of scutellarin and scutellarein on r
Tang H, Tang Y, Li N, Shi Q, Guo J, Shang E, Duan JA. Neuroprotective effects of scutellarin and scutellarein on r
Cui YH, Zhang XQ, Wang ND, Zheng MD, Yan J. Vitexin protects against ischemia/reperfusion-induced brain enc
Cui YH, Zhang XQ, Wang ND, Zheng MD, Yan J. Vitexin protects against ischemia/reperfusion-induced brain enc
Cui YH, Zhang XQ, Wang ND, Zheng MD, Yan J. Vitexin protects against ischemia/reperfusion-induced brain enc
Jiang J, Dai J, Cui H. Vitexin reverses the autophagy dysfunction to attenuate MCAO-induced cerebral ischemic
Jiang J, Dai J, Cui H. Vitexin reverses the autophagy dysfunction to attenuate MCAO-induced cerebral ischemic
Jiang J, Dai J, Cui H. Vitexin reverses the autophagy dysfunction to attenuate MCAO-induced cerebral ischemic
Jiang J, Dai J, Cui H. Vitexin reverses the autophagy dysfunction to attenuate MCAO-induced cerebral ischemic
Jiang J, Dai J, Cui H. Vitexin reverses the autophagy dysfunction to attenuate MCAO-induced cerebral ischemic
Jiang J, Dai J, Cui H. Vitexin reverses the autophagy dysfunction to attenuate MCAO-induced cerebral ischemic
Jiang J, Dai J, Cui H. Vitexin reverses the autophagy dysfunction to attenuate MCAO-induced cerebral ischemic
Jiang J, Dai J, Cui H. Vitexin reverses the autophagy dysfunction to attenuate MCAO-induced cerebral ischemic
Jiang J, Dai J, Cui H. Vitexin reverses the autophagy dysfunction to attenuate MCAO-induced cerebral ischemic
Jiang J, Dai J, Cui H. Vitexin reverses the autophagy dysfunction to attenuate MCAO-induced cerebral ischemic
Jiang J, Dai J, Cui H. Vitexin reverses the autophagy dysfunction to attenuate MCAO-induced cerebral ischemic
Pang Q, Zhao Y, Chen X, Zhao K, Zhai Q, Tu F. Apigenin Protects the Brain against Ischemia/Reperfusion Injury v
Pang Q, Zhao Y, Chen X, Zhao K, Zhai Q, Tu F. Apigenin Protects the Brain against Ischemia/Reperfusion Injury v
Pang Q, Zhao Y, Chen X, Zhao K, Zhai Q, Tu F. Apigenin Protects the Brain against Ischemia/Reperfusion Injury v
Pang Q, Zhao Y, Chen X, Zhao K, Zhai Q, Tu F. Apigenin Protects the Brain against Ischemia/Reperfusion Injury v
Pang Q, Zhao Y, Chen X, Zhao K, Zhai Q, Tu F. Apigenin Protects the Brain against Ischemia/Reperfusion Injury v
Tu F, Pang Q, Huang T, Zhao Y, Liu M, Chen X. Apigenin Ameliorates Post-Stroke Cognitive Deficits in Rats Throu
Tu F, Pang Q, Huang T, Zhao Y, Liu M, Chen X. Apigenin Ameliorates Post-Stroke Cognitive Deficits in Rats Throu
Tu F, Pang Q, Huang T, Zhao Y, Liu M, Chen X. Apigenin Ameliorates Post-Stroke Cognitive Deficits in Rats Throu
Tu F, Pang Q, Huang T, Zhao Y, Liu M, Chen X. Apigenin Ameliorates Post-Stroke Cognitive Deficits in Rats Throu
Tu F, Pang Q, Huang T, Zhao Y, Liu M, Chen X. Apigenin Ameliorates Post-Stroke Cognitive Deficits in Rats Throu
Deng Y, Xiong D, Yin C, Liu B, Shi J, Gong Q. Icariside II protects against cerebral ischemia-reperfusion injury in r
Deng Y, Xiong D, Yin C, Liu B, Shi J, Gong Q. Icariside II protects against cerebral ischemia-reperfusion injury in r
Deng Y, Xiong D, Yin C, Liu B, Shi J, Gong Q. Icariside II protects against cerebral ischemia-reperfusion injury in r
Xiong D, Deng Y, Huang B, Yin C, Liu B, Shi J, Gong Q. Icariin attenuates cerebral ischemia-reperfusion injury thr
Xiong D, Deng Y, Huang B, Yin C, Liu B, Shi J, Gong Q. Icariin attenuates cerebral ischemia-reperfusion injury thr
Xiong D, Deng Y, Huang B, Yin C, Liu B, Shi J, Gong Q. Icariin attenuates cerebral ischemia-reperfusion injury thr
Xiong D, Deng Y, Huang B, Yin C, Liu B, Shi J, Gong Q. Icariin attenuates cerebral ischemia-reperfusion injury thr
Mo ZT, Liao YL, Zheng J, Li WN. Icariin protects neurons from endoplasmic reticulum stress-induced apoptosis
Mo ZT, Liao YL, Zheng J, Li WN. Icariin protects neurons from endoplasmic reticulum stress-induced apoptosis
Mo ZT, Liao YL, Zheng J, Li WN. Icariin protects neurons from endoplasmic reticulum stress-induced apoptosis
Mo ZT, Liao YL, Zheng J, Li WN. Icariin protects neurons from endoplasmic reticulum stress-induced apoptosis
Mo ZT, Liao YL, Zheng J, Li WN. Icariin protects neurons from endoplasmic reticulum stress-induced apoptosis
Mo ZT, Liao YL, Zheng J, Li WN. Icariin protects neurons from endoplasmic reticulum stress-induced apoptosis
Mo ZT, Liao YL, Zheng J, Li WN. Icariin protects neurons from endoplasmic reticulum stress-induced apoptosis
Mo ZT, Liao YL, Zheng J, Li WN. Icariin protects neurons from endoplasmic reticulum stress-induced apoptosis
Mo ZT, Liao YL, Zheng J, Li WN. Icariin protects neurons from endoplasmic reticulum stress-induced apoptosis
Mo ZT, Liao YL, Zheng J, Li WN. Icariin protects neurons from endoplasmic reticulum stress-induced apoptosis

Mo ZT, Liao YL, Zheng J, Li WN. Icariin protects neurons from endoplasmic reticulum stress-induced apoptosis
Mo ZT, Liao YL, Zheng J, Li WN. Icariin protects neurons from endoplasmic reticulum stress-induced apoptosis
Mo ZT, Liao YL, Zheng J, Li WN. Icariin protects neurons from endoplasmic reticulum stress-induced apoptosis
Mo ZT, Liao YL, Zheng J, Li WN. Icariin protects neurons from endoplasmic reticulum stress-induced apoptosis
Dai M, Chen B, Wang X, Gao C, Yu H. Icariin enhance mild hypothermia-induced neuroprotection via inhibiting
Dai M, Chen B, Wang X, Gao C, Yu H. Icariin enhance mild hypothermia-induced neuroprotection via inhibiting
Dai M, Chen B, Wang X, Gao C, Yu H. Icariin enhance mild hypothermia-induced neuroprotection via inhibiting
Dai M, Chen B, Wang X, Gao C, Yu H. Icariin enhance mild hypothermia-induced neuroprotection via inhibiting
Dai M, Chen B, Wang X, Gao C, Yu H. Icariin enhance mild hypothermia-induced neuroprotection via inhibiting
Dai M, Chen B, Wang X, Gao C, Yu H. Icariin enhance mild hypothermia-induced neuroprotection via inhibiting
Dai M, Chen B, Wang X, Gao C, Yu H. Icariin enhance mild hypothermia-induced neuroprotection via inhibiting
Dai M, Chen B, Wang X, Gao C, Yu H. Icariin enhance mild hypothermia-induced neuroprotection via inhibiting
Dai M, Chen B, Wang X, Gao C, Yu H. Icariin enhance mild hypothermia-induced neuroprotection via inhibiting
Dai M, Chen B, Wang X, Gao C, Yu H. Icariin enhance mild hypothermia-induced neuroprotection via inhibiting
Park DJ, Jeon SJ, Kang JB, Koh PO. Quercetin Reduces Ischemic Brain Injury by Preventing Ischemia-induced Dec
Park DJ, Jeon SJ, Kang JB, Koh PO. Quercetin Reduces Ischemic Brain Injury by Preventing Ischemia-induced Dec
Park DJ, Jeon SJ, Kang JB, Koh PO. Quercetin Reduces Ischemic Brain Injury by Preventing Ischemia-induced Dec
Dai Y, Zhang H, Zhang J, Yan M. Isoquercetin attenuates oxidative stress and neuronal apoptosis after ischemia,
Dai Y, Zhang H, Zhang J, Yan M. Isoquercetin attenuates oxidative stress and neuronal apoptosis after ischemia,
Dai Y, Zhang H, Zhang J, Yan M. Isoquercetin attenuates oxidative stress and neuronal apoptosis after ischemia,
Dai Y, Zhang H, Zhang J, Yan M. Isoquercetin attenuates oxidative stress and neuronal apoptosis after ischemia,
Dai Y, Zhang H, Zhang J, Yan M. Isoquercetin attenuates oxidative stress and neuronal apoptosis after ischemia,
Guo C, Tong L, Xi M, Yang H, Dong H, Wen A. Neuroprotective effect of calycosin on cerebral ischemia and repe
Guo C, Tong L, Xi M, Yang H, Dong H, Wen A. Neuroprotective effect of calycosin on cerebral ischemia and repe
Hsu CC, Kuo TW, Liu WP, Chang CP, Lin HJ. Calycosin Preserves BDNF/TrkB Signaling and Reduces Post-Stroke N
Hsu CC, Kuo TW, Liu WP, Chang CP, Lin HJ. Calycosin Preserves BDNF/TrkB Signaling and Reduces Post-Stroke N
Wang Y, Ren Q, Zhang X, Lu H, Chen J. Neuroprotective Mechanisms of Calycosin Against Focal Cerebral Ischem
Wang Y, Ren Q, Zhang X, Lu H, Chen J. Neuroprotective Mechanisms of Calycosin Against Focal Cerebral Ischem
Wang Y, Ren Q, Zhang X, Lu H, Chen J. Neuroprotective Mechanisms of Calycosin Against Focal Cerebral Ischem
Wang Y, Ren Q, Zhang X, Lu H, Chen J. Neuroprotective Mechanisms of Calycosin Against Focal Cerebral Ischem
Wang Y, Ren Q, Zhang X, Lu H, Chen J. Neuroprotective Mechanisms of Calycosin Against Focal Cerebral Ischem
Jiao Y, Cao Y, Lu X, Wang J, Saitgareeva A, Kong X, Song C, Li J, Tian K, Zhang S, Bai M, Li S, Zhang H, Wang L. Xa
Wang CC, Ho YH, Hung CF, Kuo JR, Wang SJ. Xanthohumol, an active constituent from hope, affords protection
Wang CC, Ho YH, Hung CF, Kuo JR, Wang SJ. Xanthohumol, an active constituent from hope, affords protection
Wang CC, Ho YH, Hung CF, Kuo JR, Wang SJ. Xanthohumol, an active constituent from hope, affords protection
Wang CC, Ho YH, Hung CF, Kuo JR, Wang SJ. Xanthohumol, an active constituent from hope, affords protection
Guo H, Zhu L, Tang P, Chen D, Li Y, Li J, Bao C. Carthamin yellow improves cerebral ischemia-reperfusion injury
Guo H, Zhu L, Tang P, Chen D, Li Y, Li J, Bao C. Carthamin yellow improves cerebral ischemia-reperfusion injury
Guo H, Zhu L, Tang P, Chen D, Li Y, Li J, Bao C. Carthamin yellow improves cerebral ischemia-reperfusion injury
Guo H, Zhu L, Tang P, Chen D, Li Y, Li J, Bao C. Carthamin yellow improves cerebral ischemia-reperfusion injury
Guo H, Zhu L, Tang P, Chen D, Li Y, Li J, Bao C. Carthamin yellow improves cerebral ischemia-reperfusion injury
Guo H, Zhu L, Tang P, Chen D, Li Y, Li J, Bao C. Carthamin yellow improves cerebral ischemia-reperfusion injury
Liu P, Zou D, Chen K, Zhou Q, Gao Y, Huang Y, Zhu J, Zhang Q, Mi M. Dihydromyricetin Improves Hypobaric Hyp
Liu P, Zou D, Chen K, Zhou Q, Gao Y, Huang Y, Zhu J, Zhang Q, Mi M. Dihydromyricetin Improves Hypobaric Hyp
Hu J, Chai Y, Wang Y, Kheir MM, Li H, Yuan Z, Wan H, Xing D, Lei F, Du L. PI3K p55γ promoter activity enhancem
Hu J, Chai Y, Wang Y, Kheir MM, Li H, Yuan Z, Wan H, Xing D, Lei F, Du L. PI3K p55γ promoter activity enhancem

Hu J, Chai Y, Wang Y, Kheir MM, Li H, Yuan Z, Wan H, Xing D, Lei F, Du L. PI3K p55γ promoter activity enhancem
Maleki SN, Aboutaleb N, Souri F. Berberine confers neuroprotection in coping with focal cerebral ischemia by t
Maleki SN, Aboutaleb N, Souri F. Berberine confers neuroprotection in coping with focal cerebral ischemia by t
Maleki SN, Aboutaleb N, Souri F. Berberine confers neuroprotection in coping with focal cerebral ischemia by t
Maleki SN, Aboutaleb N, Souri F. Berberine confers neuroprotection in coping with focal cerebral ischemia by t
Chang CY, Kao TK, Chen WY, Ou YC, Li JR, Liao SL, Raung SL, Chen CJ. Tetramethylpyrazine inhibits neutrophil ac
Chang CY, Kao TK, Chen WY, Ou YC, Li JR, Liao SL, Raung SL, Chen CJ. Tetramethylpyrazine inhibits neutrophil ac
Chang CY, Kao TK, Chen WY, Ou YC, Li JR, Liao SL, Raung SL, Chen CJ. Tetramethylpyrazine inhibits neutrophil ac
Xiao X, Liu Y, Qi C, Qiu F, Chen X, Zhang J, Yang P. Neuroprotection and enhanced neurogenesis by tetramethyl
Xiao X, Liu Y, Qi C, Qiu F, Chen X, Zhang J, Yang P. Neuroprotection and enhanced neurogenesis by tetramethyl
Ding Y, Du J, Cui F, Chen L, Li K. The protective effect of ligustrazine on rats with cerebral ischemia-reperfusion
Ding Y, Du J, Cui F, Chen L, Li K. The protective effect of ligustrazine on rats with cerebral ischemia-reperfusion
Ding Y, Du J, Cui F, Chen L, Li K. The protective effect of ligustrazine on rats with cerebral ischemia-reperfusion
Ding Y, Du J, Cui F, Chen L, Li K. The protective effect of ligustrazine on rats with cerebral ischemia-reperfusion
Ding Y, Du J, Cui F, Chen L, Li K. The protective effect of ligustrazine on rats with cerebral ischemia-reperfusion
Yang XY, Liu QN, Zhang L, Jiang SQ, Gong PL. Neuroprotective effect of dauricine after transient middle cerebra
Yang XY, Liu QN, Zhang L, Jiang SQ, Gong PL. Neuroprotective effect of dauricine after transient middle cerebra
Yang XY, Liu QN, Zhang L, Jiang SQ, Gong PL. Neuroprotective effect of dauricine after transient middle cerebra
Yang XY, Liu QN, Zhang L, Jiang SQ, Gong PL. Neuroprotective effect of dauricine after transient middle cerebra
Sun R, Song Y, Li S, Ma Z, Deng X, Fu Q, Qu R, Ma S. Levo-tetrahydropalmatine Attenuates Neuron Apoptosis In
Sun R, Song Y, Li S, Ma Z, Deng X, Fu Q, Qu R, Ma S. Levo-tetrahydropalmatine Attenuates Neuron Apoptosis In
Sun R, Song Y, Li S, Ma Z, Deng X, Fu Q, Qu R, Ma S. Levo-tetrahydropalmatine Attenuates Neuron Apoptosis In
Sun R, Song Y, Li S, Ma Z, Deng X, Fu Q, Qu R, Ma S. Levo-tetrahydropalmatine Attenuates Neuron Apoptosis In
Mao XW, Pan CS, Huang P, Liu YY, Wang CS, Yan L, Hu BH, Chang X, He K, Mu HN, Li Q, Sun K, Fan JY, Han JY. Le
Mao XW, Pan CS, Huang P, Liu YY, Wang CS, Yan L, Hu BH, Chang X, He K, Mu HN, Li Q, Sun K, Fan JY, Han JY. Le
Mao XW, Pan CS, Huang P, Liu YY, Wang CS, Yan L, Hu BH, Chang X, He K, Mu HN, Li Q, Sun K, Fan JY, Han JY. Le
Wu C, Chen J, Yang R, Duan F, Li S, Chen X. Mitochondrial protective effect of neferine through the modulation
Huang L, Chen C, Zhang X, Li X, Chen Z, Yang C, Liang X, Zhu G, Xu Z. Neuroprotective Effect of Curcumin Agains
Huang L, Chen C, Zhang X, Li X, Chen Z, Yang C, Liang X, Zhu G, Xu Z. Neuroprotective Effect of Curcumin Agains
Huang L, Chen C, Zhang X, Li X, Chen Z, Yang C, Liang X, Zhu G, Xu Z. Neuroprotective Effect of Curcumin Agains
Huang L, Chen C, Zhang X, Li X, Chen Z, Yang C, Liang X, Zhu G, Xu Z. Neuroprotective Effect of Curcumin Agains
Huang L, Chen C, Zhang X, Li X, Chen Z, Yang C, Liang X, Zhu G, Xu Z. Neuroprotective Effect of Curcumin Agains
Li L, Li H, Li M. Curcumin protects against cerebral ischemia-reperfusion injury by activating JAK2/STAT3 signali
Li L, Li H, Li M. Curcumin protects against cerebral ischemia-reperfusion injury by activating JAK2/STAT3 signali
Li L, Li H, Li M. Curcumin protects against cerebral ischemia-reperfusion injury by activating JAK2/STAT3 signali
Li L, Li H, Li M. Curcumin protects against cerebral ischemia-reperfusion injury by activating JAK2/STAT3 signali
Li L, Li H, Li M. Curcumin protects against cerebral ischemia-reperfusion injury by activating JAK2/STAT3 signali
Lu Z, Liu Y, Shi Y, Shi X, Wang X, Xu C, Zhao H, Dong Q. Curcumin protects cortical neurons against oxygen and g
Lu Z, Liu Y, Shi Y, Shi X, Wang X, Xu C, Zhao H, Dong Q. Curcumin protects cortical neurons against oxygen and g
Lu Z, Liu Y, Shi Y, Shi X, Wang X, Xu C, Zhao H, Dong Q. Curcumin protects cortical neurons against oxygen and g
Xie CJ, Gu AP, Cai J, Wu Y, Chen RC. Curcumin protects neural cells against ischemic injury in N2a cells and mou
Xie CJ, Gu AP, Cai J, Wu Y, Chen RC. Curcumin protects neural cells against ischemic injury in N2a cells and mou
Xie CJ, Gu AP, Cai J, Wu Y, Chen RC. Curcumin protects neural cells against ischemic injury in N2a cells and mou
Xie CJ, Gu AP, Cai J, Wu Y, Chen RC. Curcumin protects neural cells against ischemic injury in N2a cells and mou
Hou Y, Wang J, Feng J. The neuroprotective effects of curcumin are associated with the regulation of the recipr
Hou Y, Wang J, Feng J. The neuroprotective effects of curcumin are associated with the regulation of the recipr

Zhang Y, Fang M, Sun Y, Zhang T, Shi N, Li J, Jin L, Liu K, Fu J. Curcumin attenuates cerebral ischemia injury in Sp
Liu L, Zhang W, Wang L, Li Y, Tan B, Lu X, Deng Y, Zhang Y, Guo X, Mu J, Yu G. Curcumin prevents cerebral ische
Liu L, Zhang W, Wang L, Li Y, Tan B, Lu X, Deng Y, Zhang Y, Guo X, Mu J, Yu G. Curcumin prevents cerebral ische
Liu L, Zhang W, Wang L, Li Y, Tan B, Lu X, Deng Y, Zhang Y, Guo X, Mu J, Yu G. Curcumin prevents cerebral ische
Liu L, Zhang W, Wang L, Li Y, Tan B, Lu X, Deng Y, Zhang Y, Guo X, Mu J, Yu G. Curcumin prevents cerebral ische
Simão F, Matté A, Pagnussat AS, Netto CA, Salbego CG. Resveratrol preconditioning modulates inflammatory re
Simão F, Matté A, Pagnussat AS, Netto CA, Salbego CG. Resveratrol preconditioning modulates inflammatory re
Simão F, Matté A, Pagnussat AS, Netto CA, Salbego CG. Resveratrol preconditioning modulates inflammatory re
Simão F, Matté A, Pagnussat AS, Netto CA, Salbego CG. Resveratrol preconditioning modulates inflammatory re
Simão F, Matté A, Pagnussat AS, Netto CA, Salbego CG. Resveratrol preconditioning modulates inflammatory re
Wei H, Wang S, Zhen L, Yang Q, Wu Z, Lei X, Lv J, Xiong L, Xue R. Resveratrol attenuates the blood-brain barrier
Wei H, Wang S, Zhen L, Yang Q, Wu Z, Lei X, Lv J, Xiong L, Xue R. Resveratrol attenuates the blood-brain barrier
Wei H, Wang S, Zhen L, Yang Q, Wu Z, Lei X, Lv J, Xiong L, Xue R. Resveratrol attenuates the blood-brain barrier
He Q, Li Z, Wang Y, Hou Y, Li L, Zhao J. Resveratrol alleviates cerebral ischemia/reperfusion injury in rats by inhi
He Q, Li Z, Wang Y, Hou Y, Li L, Zhao J. Resveratrol alleviates cerebral ischemia/reperfusion injury in rats by inhi
He Q, Li Z, Wang Y, Hou Y, Li L, Zhao J. Resveratrol alleviates cerebral ischemia/reperfusion injury in rats by inhi
He Q, Li Z, Wang Y, Hou Y, Li L, Zhao J. Resveratrol alleviates cerebral ischemia/reperfusion injury in rats by inhi
He Q, Li Z, Wang Y, Hou Y, Li L, Zhao J. Resveratrol alleviates cerebral ischemia/reperfusion injury in rats by inhi
Chen S, Yin ZJ, Jiang C, Ma ZQ, Fu Q, Qu R, Ma SP. Asiaticoside attenuates memory impairment induced by tran
Chen S, Yin ZJ, Jiang C, Ma ZQ, Fu Q, Qu R, Ma SP. Asiaticoside attenuates memory impairment induced by tran
Chen S, Yin ZJ, Jiang C, Ma ZQ, Fu Q, Qu R, Ma SP. Asiaticoside attenuates memory impairment induced by tran
Chen S, Yin ZJ, Jiang C, Ma ZQ, Fu Q, Qu R, Ma SP. Asiaticoside attenuates memory impairment induced by tran
Chen S, Yin ZJ, Jiang C, Ma ZQ, Fu Q, Qu R, Ma SP. Asiaticoside attenuates memory impairment induced by tran
Chen S, Yin ZJ, Jiang C, Ma ZQ, Fu Q, Qu R, Ma SP. Asiaticoside attenuates memory impairment induced by tran
Qi FY, Yang L, Tian Z, Zhao MG, Liu SB, An JZ. Neuroprotective effects of Asiaticoside. Neural Regen Res. 2014 Ju
Qi FY, Yang L, Tian Z, Zhao MG, Liu SB, An JZ. Neuroprotective effects of Asiaticoside. Neural Regen Res. 2014 Ju
Qi FY, Yang L, Tian Z, Zhao MG, Liu SB, An JZ. Neuroprotective effects of Asiaticoside. Neural Regen Res. 2014 Ju
Liu X, Wen S, Yan F, Liu K, Liu L, Wang L, Zhao S, Ji X. Salidroside provides neuroprotection by modulating micro
Liu X, Wen S, Yan F, Liu K, Liu L, Wang L, Zhao S, Ji X. Salidroside provides neuroprotection by modulating micro
Liu X, Wen S, Yan F, Liu K, Liu L, Wang L, Zhao S, Ji X. Salidroside provides neuroprotection by modulating micro
Liu X, Wen S, Yan F, Liu K, Liu L, Wang L, Zhao S, Ji X. Salidroside provides neuroprotection by modulating micro
Liu X, Wen S, Yan F, Liu K, Liu L, Wang L, Zhao S, Ji X. Salidroside provides neuroprotection by modulating micro
Liu X, Wen S, Yan F, Liu K, Liu L, Wang L, Zhao S, Ji X. Salidroside provides neuroprotection by modulating micro
Liu X, Wen S, Yan F, Liu K, Liu L, Wang L, Zhao S, Ji X. Salidroside provides neuroprotection by modulating micro
Liu X, Wen S, Yan F, Liu K, Liu L, Wang L, Zhao S, Ji X. Salidroside provides neuroprotection by modulating micro
Liu X, Wen S, Yan F, Liu K, Liu L, Wang L, Zhao S, Ji X. Salidroside provides neuroprotection by modulating micro
Liu X, Wen S, Yan F, Liu K, Liu L, Wang L, Zhao S, Ji X. Salidroside provides neuroprotection by modulating micro
Liu X, Wen S, Yan F, Liu K, Liu L, Wang L, Zhao S, Ji X. Salidroside provides neuroprotection by modulating micro
Liu X, Wen S, Yan F, Liu K, Liu L, Wang L, Zhao S, Ji X. Salidroside provides neuroprotection by modulating micro
Liu J, Ma W, Zang CH, Wang GD, Zhang SJ, Wu HJ, Zhu KW, Xiang XL, Li CY, Liu KP, Guo JH, Li LY. Salidroside inhi
Liu J, Ma W, Zang CH, Wang GD, Zhang SJ, Wu HJ, Zhu KW, Xiang XL, Li CY, Liu KP, Guo JH, Li LY. Salidroside inhi
Liu J, Ma W, Zang CH, Wang GD, Zhang SJ, Wu HJ, Zhu KW, Xiang XL, Li CY, Liu KP, Guo JH, Li LY. Salidroside inhi
Liu J, Ma W, Zang CH, Wang GD, Zhang SJ, Wu HJ, Zhu KW, Xiang XL, Li CY, Liu KP, Guo JH, Li LY. Salidroside inhi
Zhang X, Lai W, Ying X, Xu L, Chu K, Brown J, Chen L, Hong G. Salidroside Reduces Inflammation and Brain Injur
Zhang X, Lai W, Ying X, Xu L, Chu K, Brown J, Chen L, Hong G. Salidroside Reduces Inflammation and Brain Injur
Zhang X, Lai W, Ying X, Xu L, Chu K, Brown J, Chen L, Hong G. Salidroside Reduces Inflammation and Brain Injur

Zhang X, Lai W, Ying X, Xu L, Chu K, Brown J, Chen L, Hong G. Salidroside Reduces Inflammation and Brain Injury
Fu C, Zhang X, Lu Y, Wang F, Xu Z, Liu S, Zheng H, Liu X. Geniposide inhibits NLRP3 inflammasome activation via
Fu C, Zhang X, Lu Y, Wang F, Xu Z, Liu S, Zheng H, Liu X. Geniposide inhibits NLRP3 inflammasome activation via
Fu C, Zhang X, Lu Y, Wang F, Xu Z, Liu S, Zheng H, Liu X. Geniposide inhibits NLRP3 inflammasome activation via
Fu C, Zhang X, Lu Y, Wang F, Xu Z, Liu S, Zheng H, Liu X. Geniposide inhibits NLRP3 inflammasome activation via
Fu C, Zhang X, Lu Y, Wang F, Xu Z, Liu S, Zheng H, Liu X. Geniposide inhibits NLRP3 inflammasome activation via
Huang B, Chen P, Huang L, Li S, Zhu R, Sheng T, Yu W, Chen Z, Wang T. Geniposide Attenuates Post-Ischaemic N
Yao H, Liu W, Liao H, Sheng T, Chen P, Zhou H, Pan Y, Xie J, Zhang Q, Zou Z, Chen Z. Geniposide attenuates post
Liu YR, Li PW, Suo JJ, Sun Y, Zhang BA, Lu H, Zhu HC, Zhang GB. Catalpol provides protective effects against cere
Liu YR, Li PW, Suo JJ, Sun Y, Zhang BA, Lu H, Zhu HC, Zhang GB. Catalpol provides protective effects against cere
Liu YR, Li PW, Suo JJ, Sun Y, Zhang BA, Lu H, Zhu HC, Zhang GB. Catalpol provides protective effects against cere
Guo Y, Xu X, Li Q, Li Z, Du F. Anti-inflammation effects of picroside 2 in cerebral ischemic injury rats. Behav Brai
Guo Y, Xu X, Li Q, Li Z, Du F. Anti-inflammation effects of picroside 2 in cerebral ischemic injury rats. Behav Brai
Guo Y, Xu X, Li Q, Li Z, Du F. Anti-inflammation effects of picroside 2 in cerebral ischemic injury rats. Behav Brai
Li Q, Li Z, Xu XY, Guo YL, Du F. Neuroprotective properties of picroside II in a rat model of focal cerebral ischem
Wang T, Zhai L, Zhang H, Zhao L, Guo Y. Picroside II Inhibits the MEK-ERK1/2-COX2 Signal Pathway to Prevent C
Wang T, Zhai L, Zhang H, Zhao L, Guo Y. Picroside II Inhibits the MEK-ERK1/2-COX2 Signal Pathway to Prevent C
Wang T, Zhai L, Zhang H, Zhao L, Guo Y. Picroside II Inhibits the MEK-ERK1/2-COX2 Signal Pathway to Prevent C
Wang T, Zhai L, Zhang H, Zhao L, Guo Y. Picroside II Inhibits the MEK-ERK1/2-COX2 Signal Pathway to Prevent C
Wang T, Zhai L, Zhang H, Zhao L, Guo Y. Picroside II Inhibits the MEK-ERK1/2-COX2 Signal Pathway to Prevent C
Wang T, Zhai L, Zhang H, Zhao L, Guo Y. Picroside II Inhibits the MEK-ERK1/2-COX2 Signal Pathway to Prevent C
Wang T, Zhai L, Zhang H, Zhao L, Guo Y. Picroside II Inhibits the MEK-ERK1/2-COX2 Signal Pathway to Prevent C
Wang T, Zhai L, Zhang H, Zhao L, Guo Y. Picroside II Inhibits the MEK-ERK1/2-COX2 Signal Pathway to Prevent C
Wang T, Zhai L, Zhang H, Zhao L, Guo Y. Picroside II Inhibits the MEK-ERK1/2-COX2 Signal Pathway to Prevent C
Wang T, Zhai L, Zhang H, Zhao L, Guo Y. Picroside II Inhibits the MEK-ERK1/2-COX2 Signal Pathway to Prevent C
Zhang H, Zhai L, Wang T, Li S, Guo Y. Picroside II Exerts a Neuroprotective Effect by Inhibiting the Mitochondria
Zhang H, Zhai L, Wang T, Li S, Guo Y. Picroside II Exerts a Neuroprotective Effect by Inhibiting the Mitochondria
Zhang H, Zhai L, Wang T, Li S, Guo Y. Picroside II Exerts a Neuroprotective Effect by Inhibiting the Mitochondria
Zhang H, Zhai L, Wang T, Li S, Guo Y. Picroside II Exerts a Neuroprotective Effect by Inhibiting the Mitochondria
Wang QY, Liu F, Wu FJ, Li JL. [Effects of ginsenoside Rg1 on the expressions of p-eRK1/2 and p-JNK in local cerel
Wang QY, Liu F, Wu FJ, Li JL. [Effects of ginsenoside Rg1 on the expressions of p-eRK1/2 and p-JNK in local cerel
Wang QY, Liu F, Wu FJ, Li JL. [Effects of ginsenoside Rg1 on the expressions of p-eRK1/2 and p-JNK in local cerel
Liu JW, Ren YL, Liu XL, Xia HL, Zhang HL, Jin SH, Dai QX, Wang JL. [Effect of ginsenoside Rb1 on cerebral infarctic
Ye JT, Li FT, Huang SL, Xue JL, Aihaiti Y, Wu H, Liu RX, Cheng B. Effects of ginsenoside Rb1 on spinal cord ischem
Ye JT, Li FT, Huang SL, Xue JL, Aihaiti Y, Wu H, Liu RX, Cheng B. Effects of ginsenoside Rb1 on spinal cord ischem
Ye JT, Li FT, Huang SL, Xue JL, Aihaiti Y, Wu H, Liu RX, Cheng B. Effects of ginsenoside Rb1 on spinal cord ischem
Li M, Qu YZ, Zhao ZW, Wu SX, Liu YY, Wei XY, Gao L, Gao GD. Astragaloside IV protects against focal cerebral isc
Li M, Qu YZ, Zhao ZW, Wu SX, Liu YY, Wei XY, Gao L, Gao GD. Astragaloside IV protects against focal cerebral isc
Li M, Li H, Fang F, Deng X, Ma S. Astragaloside IV attenuates cognitive impairments induced by transient cerebr
Yin F, Zhou H, Fang Y, Li C, He Y, Yu L, Wan H, Yang J. Astragaloside IV alleviates ischemia reperfusion-induced a
Yin F, Zhou H, Fang Y, Li C, He Y, Yu L, Wan H, Yang J. Astragaloside IV alleviates ischemia reperfusion-induced a
Yin F, Zhou H, Fang Y, Li C, He Y, Yu L, Wan H, Yang J. Astragaloside IV alleviates ischemia reperfusion-induced a
Yin F, Zhou H, Fang Y, Li C, He Y, Yu L, Wan H, Yang J. Astragaloside IV alleviates ischemia reperfusion-induced a
Yin F, Zhou H, Fang Y, Li C, He Y, Yu L, Wan H, Yang J. Astragaloside IV alleviates ischemia reperfusion-induced a
Yin F, Zhou H, Fang Y, Li C, He Y, Yu L, Wan H, Yang J. Astragaloside IV alleviates ischemia reperfusion-induced a
Yin F, Zhou H, Fang Y, Li C, He Y, Yu L, Wan H, Yang J. Astragaloside IV alleviates ischemia reperfusion-induced a

Yin F, Zhou H, Fang Y, Li C, He Y, Yu L, Wan H, Yang J. Astragaloside IV alleviates ischemia reperfusion-induced a
Yin F, Zhou H, Fang Y, Li C, He Y, Yu L, Wan H, Yang J. Astragaloside IV alleviates ischemia reperfusion-induced a
Xiao L, Dai Z, Tang W, Liu C, Tang B. Astragaloside IV Alleviates Cerebral Ischemia-Reperfusion Injury through N
Xiao L, Dai Z, Tang W, Liu C, Tang B. Astragaloside IV Alleviates Cerebral Ischemia-Reperfusion Injury through N
Xiao L, Dai Z, Tang W, Liu C, Tang B. Astragaloside IV Alleviates Cerebral Ischemia-Reperfusion Injury through N
Xiao L, Dai Z, Tang W, Liu C, Tang B. Astragaloside IV Alleviates Cerebral Ischemia-Reperfusion Injury through N
Xiao L, Dai Z, Tang W, Liu C, Tang B. Astragaloside IV Alleviates Cerebral Ischemia-Reperfusion Injury through N
Xiao L, Dai Z, Tang W, Liu C, Tang B. Astragaloside IV Alleviates Cerebral Ischemia-Reperfusion Injury through N
Jia D, Deng Y, Gao J, Liu X, Chu J, Shu Y. Neuroprotective effect of Panax notoginseng plysaccharides against foc
Jia D, Deng Y, Gao J, Liu X, Chu J, Shu Y. Neuroprotective effect of Panax notoginseng plysaccharides against foc
Jia D, Deng Y, Gao J, Liu X, Chu J, Shu Y. Neuroprotective effect of Panax notoginseng plysaccharides against foc
Jia D, Deng Y, Gao J, Liu X, Chu J, Shu Y. Neuroprotective effect of Panax notoginseng plysaccharides against foc
Chern CM, Liou KT, Wang YH, Liao JF, Yen JC, Shen YC. Andrographolide inhibits PI3K/AKT-dependent NOX2 anc
Chern CM, Liou KT, Wang YH, Liao JF, Yen JC, Shen YC. Andrographolide inhibits PI3K/AKT-dependent NOX2 anc
Yen TL, Hsu WH, Huang SK, Lu WJ, Chang CC, Lien LM, Hsiao G, Sheu JR, Lin KH. A novel bioactivity of andrograp
Yen TL, Chen RJ, Jayakumar T, Lu WJ, Hsieh CY, Hsu MJ, Yang CH, Chang CC, Lin YK, Lin KH, Sheu JR. Andrograph
Li Y, Xiang LL, Miao JX, Miao MS, Wang C. Protective effects of andrographolide against cerebral ischemia-repe
Li Y, Xiang LL, Miao JX, Miao MS, Wang C. Protective effects of andrographolide against cerebral ischemia-repe
Li Y, Xiang LL, Miao JX, Miao MS, Wang C. Protective effects of andrographolide against cerebral ischemia-repe
Li Y, Xiang LL, Miao JX, Miao MS, Wang C. Protective effects of andrographolide against cerebral ischemia-repe
Li Y, Xiang LL, Miao JX, Miao MS, Wang C. Protective effects of andrographolide against cerebral ischemia-repe
Liu YG, Li FJ, Wang J, Wang XD. [Effects of Ginkgolide B on inflammation induced by cerebral ischemia-reperfus
Liu YG, Li FJ, Wang J, Wang XD. [Effects of Ginkgolide B on inflammation induced by cerebral ischemia-reperfus
Liu YG, Li FJ, Wang J, Wang XD. [Effects of Ginkgolide B on inflammation induced by cerebral ischemia-reperfus
Liu YG, Li FJ, Wang J, Wang XD. [Effects of Ginkgolide B on inflammation induced by cerebral ischemia-reperfus
Liu Q, Jin Z, Xu Z, Yang H, Li L, Li G, Li F, Gu S, Zong S, Zhou J, Cao L, Wang Z, Xiao W. Antioxidant effects of ginkg
Liu Q, Jin Z, Xu Z, Yang H, Li L, Li G, Li F, Gu S, Zong S, Zhou J, Cao L, Wang Z, Xiao W. Antioxidant effects of ginkg
Liu Q, Jin Z, Xu Z, Yang H, Li L, Li G, Li F, Gu S, Zong S, Zhou J, Cao L, Wang Z, Xiao W. Antioxidant effects of ginkg
Liu Q, Jin Z, Xu Z, Yang H, Li L, Li G, Li F, Gu S, Zong S, Zhou J, Cao L, Wang Z, Xiao W. Antioxidant effects of ginkg
Liu Q, Jin Z, Xu Z, Yang H, Li L, Li G, Li F, Gu S, Zong S, Zhou J, Cao L, Wang Z, Xiao W. Antioxidant effects of ginkg
Liu Q, Jin Z, Xu Z, Yang H, Li L, Li G, Li F, Gu S, Zong S, Zhou J, Cao L, Wang Z, Xiao W. Antioxidant effects of ginkg
Liu Q, Jin Z, Xu Z, Yang H, Li L, Li G, Li F, Gu S, Zong S, Zhou J, Cao L, Wang Z, Xiao W. Antioxidant effects of ginkg
Liu Q, Jin Z, Xu Z, Yang H, Li L, Li G, Li F, Gu S, Zong S, Zhou J, Cao L, Wang Z, Xiao W. Antioxidant effects of ginkg
Zhang W, Song JK, Yan R, Li L, Xiao ZY, Zhou WX, Wang ZZ, Xiao W, Du GH. Diterpene ginkgolides protect agains
Zhang W, Song JK, Yan R, Li L, Xiao ZY, Zhou WX, Wang ZZ, Xiao W, Du GH. Diterpene ginkgolides protect agains
Zhang W, Song JK, Yan R, Li L, Xiao ZY, Zhou WX, Wang ZZ, Xiao W, Du GH. Diterpene ginkgolides protect agains
Li X, Huang L, Liu G, Fan W, Li B, Liu R, Wang Z, Fan Q, Xiao W, Li Y, Fang W. Ginkgo diterpene lactones inhibit ce
Li X, Huang L, Liu G, Fan W, Li B, Liu R, Wang Z, Fan Q, Xiao W, Li Y, Fang W. Ginkgo diterpene lactones inhibit ce
Li X, Huang L, Liu G, Fan W, Li B, Liu R, Wang Z, Fan Q, Xiao W, Li Y, Fang W. Ginkgo diterpene lactones inhibit ce
Li X, Huang L, Liu G, Fan W, Li B, Liu R, Wang Z, Fan Q, Xiao W, Li Y, Fang W. Ginkgo diterpene lactones inhibit ce
Khadankhuu B, Fei Y, Li X, Fang W, Li Y. 10-O-(N N-Dimethylaminoethyl)-Ginkgolide B Methane-Sulfonate (XQ-1
Khadankhuu B, Fei Y, Li X, Fang W, Li Y. 10-O-(N N-Dimethylaminoethyl)-Ginkgolide B Methane-Sulfonate (XQ-1
Khadankhuu B, Fei Y, Li X, Fang W, Li Y. 10-O-(N N-Dimethylaminoethyl)-Ginkgolide B Methane-Sulfonate (XQ-1
Liu R, Zhang L, Lan X, Li L, Zhang TT, Sun JH, Du GH. Protection by borneol on cortical neurons against oxygen-gl
Liu R, Zhang L, Lan X, Li L, Zhang TT, Sun JH, Du GH. Protection by borneol on cortical neurons against oxygen-gl
Liu R, Zhang L, Lan X, Li L, Zhang TT, Sun JH, Du GH. Protection by borneol on cortical neurons against oxygen-gl

Liu R, Zhang L, Lan X, Li L, Zhang TT, Sun JH, Du GH. Protection by borneol on cortical neurons against oxygen-gl
Dong T, Chen N, Ma X, Wang J, Wen J, Xie Q, Ma R. The protective roles of L-borneolum, D-borneolum and synt
Dong T, Chen N, Ma X, Wang J, Wen J, Xie Q, Ma R. The protective roles of L-borneolum, D-borneolum and synt
Dong T, Chen N, Ma X, Wang J, Wen J, Xie Q, Ma R. The protective roles of L-borneolum, D-borneolum and synt
Dong T, Chen N, Ma X, Wang J, Wen J, Xie Q, Ma R. The protective roles of L-borneolum, D-borneolum and synt
Dong T, Chen N, Ma X, Wang J, Wen J, Xie Q, Ma R. The protective roles of L-borneolum, D-borneolum and synt
Dong T, Chen N, Ma X, Wang J, Wen J, Xie Q, Ma R. The protective roles of L-borneolum, D-borneolum and synt
Dong T, Chen N, Ma X, Wang J, Wen J, Xie Q, Ma R. The protective roles of L-borneolum, D-borneolum and synt
Zhang W, Wen J, Jiang Y, Hu Q, Wang J, Wei S, Li H, Ma X. l-Borneol ameliorates cerebral ischaemia by downreg
Zhang W, Wen J, Jiang Y, Hu Q, Wang J, Wei S, Li H, Ma X. l-Borneol ameliorates cerebral ischaemia by downreg
Zhang W, Wen J, Jiang Y, Hu Q, Wang J, Wei S, Li H, Ma X. l-Borneol ameliorates cerebral ischaemia by downreg
Zhang W, Wen J, Jiang Y, Hu Q, Wang J, Wei S, Li H, Ma X. l-Borneol ameliorates cerebral ischaemia by downreg
Zhang W, Wen J, Jiang Y, Hu Q, Wang J, Wei S, Li H, Ma X. l-Borneol ameliorates cerebral ischaemia by downreg
Zhang W, Wen J, Jiang Y, Hu Q, Wang J, Wei S, Li H, Ma X. l-Borneol ameliorates cerebral ischaemia by downreg
Zhang W, Wen J, Jiang Y, Hu Q, Wang J, Wei S, Li H, Ma X. l-Borneol ameliorates cerebral ischaemia by downreg
Zhang W, Wen J, Jiang Y, Hu Q, Wang J, Wei S, Li H, Ma X. l-Borneol ameliorates cerebral ischaemia by downreg
Zheng M, Zhou M, Chen M, Lu Y, Shi D, Wang J, Liu C. Neuroprotective Effect of Daidzein Extracted From Puera
Zheng M, Zhou M, Chen M, Lu Y, Shi D, Wang J, Liu C. Neuroprotective Effect of Daidzein Extracted From Puera
Zheng M, Zhou M, Chen M, Lu Y, Shi D, Wang J, Liu C. Neuroprotective Effect of Daidzein Extracted From Puera
Zheng M, Zhou M, Chen M, Lu Y, Shi D, Wang J, Liu C. Neuroprotective Effect of Daidzein Extracted From Puera
Ahn SM, Kim HN, Kim YR, Choi YW, Kim CM, Shin HK, Choi BT. Emodin from Polygonum multiflorum ameliorate
Ahn SM, Kim HN, Kim YR, Choi YW, Kim CM, Shin HK, Choi BT. Emodin from Polygonum multiflorum ameliorate
Ahn SM, Kim HN, Kim YR, Choi YW, Kim CM, Shin HK, Choi BT. Emodin from Polygonum multiflorum ameliorate
Leung SW, Lai JH, Wu JC, Tsai YR, Chen YH, Kang SJ, Chiang YH, Chang CF, Chen KY. Neuroprotective Effects of E
Leung SW, Lai JH, Wu JC, Tsai YR, Chen YH, Kang SJ, Chiang YH, Chang CF, Chen KY. Neuroprotective Effects of E
Leung SW, Lai JH, Wu JC, Tsai YR, Chen YH, Kang SJ, Chiang YH, Chang CF, Chen KY. Neuroprotective Effects of E
Leung SW, Lai JH, Wu JC, Tsai YR, Chen YH, Kang SJ, Chiang YH, Chang CF, Chen KY. Neuroprotective Effects of E
Cheng Z, He W, Zhou X, Lv Q, Xu X, Yang S, Zhao C, Guo L. Cordycepin protects against cerebral ischemia/reperf
Cheng Z, He W, Zhou X, Lv Q, Xu X, Yang S, Zhao C, Guo L. Cordycepin protects against cerebral ischemia/reperf
Cheng Z, He W, Zhou X, Lv Q, Xu X, Yang S, Zhao C, Guo L. Cordycepin protects against cerebral ischemia/reperf
Cheng Z, He W, Zhou X, Lv Q, Xu X, Yang S, Zhao C, Guo L. Cordycepin protects against cerebral ischemia/reperf
Cheng Z, He W, Zhou X, Lv Q, Xu X, Yang S, Zhao C, Guo L. Cordycepin protects against cerebral ischemia/reperf
Dong ZS, Cao ZP, Shang YJ, Liu QY, Wu BY, Liu WX, Li CH. Neuroprotection of cordycepin in NMDA-induced excit
Wei P, Wang K, Luo C, Huang Y, Misilimu D, Wen H, Jin P, Li C, Gong Y, Gao Y. Cordycepin confers long-term neu
Wei P, Wang K, Luo C, Huang Y, Misilimu D, Wen H, Jin P, Li C, Gong Y, Gao Y. Cordycepin confers long-term neu
Wei P, Wang K, Luo C, Huang Y, Misilimu D, Wen H, Jin P, Li C, Gong Y, Gao Y. Cordycepin confers long-term neu
Wei P, Wang K, Luo C, Huang Y, Misilimu D, Wen H, Jin P, Li C, Gong Y, Gao Y. Cordycepin confers long-term neu
Wei P, Wang K, Luo C, Huang Y, Misilimu D, Wen H, Jin P, Li C, Gong Y, Gao Y. Cordycepin confers long-term neu
Wei P, Wang K, Luo C, Huang Y, Misilimu D, Wen H, Jin P, Li C, Gong Y, Gao Y. Cordycepin confers long-term neu
Che N, Ma Y, Xin Y. Protective Role of Fucoidan in Cerebral Ischemia-Reperfusion Injury through Inhibition of M
Che N, Ma Y, Xin Y. Protective Role of Fucoidan in Cerebral Ischemia-Reperfusion Injury through Inhibition of M
Che N, Ma Y, Xin Y. Protective Role of Fucoidan in Cerebral Ischemia-Reperfusion Injury through Inhibition of M
Che N, Ma Y, Xin Y. Protective Role of Fucoidan in Cerebral Ischemia-Reperfusion Injury through Inhibition of M
Che N, Ma Y, Xin Y. Protective Role of Fucoidan in Cerebral Ischemia-Reperfusion Injury through Inhibition of M
Che N, Ma Y, Xin Y. Protective Role of Fucoidan in Cerebral Ischemia-Reperfusion Injury through Inhibition of M
Che N, Ma Y, Xin Y. Protective Role of Fucoidan in Cerebral Ischemia-Reperfusion Injury through Inhibition of M

Che N, Ma Y, Xin Y. Protective Role of Fucoidan in Cerebral Ischemia-Reperfusion Injury through Inhibition of M
Che N, Ma Y, Xin Y. Protective Role of Fucoidan in Cerebral Ischemia-Reperfusion Injury through Inhibition of M
Che N, Ma Y, Xin Y. Protective Role of Fucoidan in Cerebral Ischemia-Reperfusion Injury through Inhibition of M
Ahn JH, Shin MC, Kim DW, Kim H, Song M, Lee TK, Lee JC, Kim H, Cho JH, Kim YM, Kim JD, Choi SY, Won MH, Pa
Ahn JH, Shin MC, Kim DW, Kim H, Song M, Lee TK, Lee JC, Kim H, Cho JH, Kim YM, Kim JD, Choi SY, Won MH, Pa
Ahn JH, Shin MC, Kim DW, Kim H, Song M, Lee TK, Lee JC, Kim H, Cho JH, Kim YM, Kim JD, Choi SY, Won MH, Pa
Ahn JH, Shin MC, Kim DW, Kim H, Song M, Lee TK, Lee JC, Kim H, Cho JH, Kim YM, Kim JD, Choi SY, Won MH, Pa
Ahn JH, Shin MC, Kim DW, Kim H, Song M, Lee TK, Lee JC, Kim H, Cho JH, Kim YM, Kim JD, Choi SY, Won MH, Pa
Kim H, Ahn JH, Song M, Kim DW, Lee TK, Lee JC, Kim YM, Kim JD, Cho JH, Hwang IK, Yan BC, Won MH, Park JH. F
Kim H, Ahn JH, Song M, Kim DW, Lee TK, Lee JC, Kim YM, Kim JD, Cho JH, Hwang IK, Yan BC, Won MH, Park JH. F
Kim H, Ahn JH, Song M, Kim DW, Lee TK, Lee JC, Kim YM, Kim JD, Cho JH, Hwang IK, Yan BC, Won MH, Park JH. F
Kim H, Ahn JH, Song M, Kim DW, Lee TK, Lee JC, Kim YM, Kim JD, Cho JH, Hwang IK, Yan BC, Won MH, Park JH. F
Kim H, Ahn JH, Song M, Kim DW, Lee TK, Lee JC, Kim YM, Kim JD, Cho JH, Hwang IK, Yan BC, Won MH, Park JH. F
Meyer E, Bonato JM, Mori MA, Mattos BA, Guimarães FS, Milani H, de Campos AC, de Oliveira RMW. Cannabid
Meyer E, Bonato JM, Mori MA, Mattos BA, Guimarães FS, Milani H, de Campos AC, de Oliveira RMW. Cannabid
Pazos MR, Cinquina V, Gómez A, Layunta R, Santos M, Fernández-Ruiz J, Martínez-Orgado J. Cannabidiol admin
Schiavon AP, Soares LM, Bonato JM, Milani H, Guimarães FS, Weffort de Oliveira RM. Protective effects of canr
Mori MA, Meyer E, Soares LM, Milani H, Guimarães FS, de Oliveira RMW. Cannabidiol reduces neuroinflammat
Mori MA, Meyer E, Soares LM, Milani H, Guimarães FS, de Oliveira RMW. Cannabidiol reduces neuroinflammat
Mori MA, Meyer E, Soares LM, Milani H, Guimarães FS, de Oliveira RMW. Cannabidiol reduces neuroinflammat
Mori MA, Meyer E, Soares LM, Milani H, Guimarães FS, de Oliveira RMW. Cannabidiol reduces neuroinflammat
Chen YF, Wang YW, Huang WS, Lee MM, Wood WG, Leung YM, Tsai HY. Trans-Cinnamaldehyde, An Essential O
Chen YF, Wang YW, Huang WS, Lee MM, Wood WG, Leung YM, Tsai HY. Trans-Cinnamaldehyde, An Essential O
Chen YF, Wang YW, Huang WS, Lee MM, Wood WG, Leung YM, Tsai HY. Trans-Cinnamaldehyde, An Essential O
Chen YF, Wang YW, Huang WS, Lee MM, Wood WG, Leung YM, Tsai HY. Trans-Cinnamaldehyde, An Essential O
Chen YF, Wang YW, Huang WS, Lee MM, Wood WG, Leung YM, Tsai HY. Trans-Cinnamaldehyde, An Essential O
Chen YF, Wang YW, Huang WS, Lee MM, Wood WG, Leung YM, Tsai HY. Trans-Cinnamaldehyde, An Essential O
Chen YF, Wang YW, Huang WS, Lee MM, Wood WG, Leung YM, Tsai HY. Trans-Cinnamaldehyde, An Essential O
Chen YF, Wang YW, Huang WS, Lee MM, Wood WG, Leung YM, Tsai HY. Trans-Cinnamaldehyde, An Essential O
Chen YF, Wang YW, Huang WS, Lee MM, Wood WG, Leung YM, Tsai HY. Trans-Cinnamaldehyde, An Essential O
Chen YF, Wang YW, Huang WS, Lee MM, Wood WG, Leung YM, Tsai HY. Trans-Cinnamaldehyde, An Essential O

emia/reperfusion injury in gerbils via anti-oxidative and anti-apoptotic pathways. Brain Res Bull. 2011 Jul 1!
emia/reperfusion injury in gerbils via anti-oxidative and anti-apoptotic pathways. Brain Res Bull. 2011 Jul 1!
emia/reperfusion injury in gerbils via anti-oxidative and anti-apoptotic pathways. Brain Res Bull. 2011 Jul 1!
emia/reperfusion injury in gerbils via anti-oxidative and anti-apoptotic pathways. Brain Res Bull. 2011 Jul 1!
ory impairment by inhibiting the phosphorylation of CaMKII in hippocampus. Brain Res. 2016 Jul 1;1642:95-
APK cascades are involved in baicalin's neuroprotection against gerbil global ischemia/reperfusion injury. Br
APK cascades are involved in baicalin's neuroprotection against gerbil global ischemia/reperfusion injury. Br
APK cascades are involved in baicalin's neuroprotection against gerbil global ischemia/reperfusion injury. Br
APK cascades are involved in baicalin's neuroprotection against gerbil global ischemia/reperfusion injury. Br
APK cascades are involved in baicalin's neuroprotection against gerbil global ischemia/reperfusion injury. Br
APK cascades are involved in baicalin's neuroprotection against gerbil global ischemia/reperfusion injury. Br
APK cascades are involved in baicalin's neuroprotection against gerbil global ischemia/reperfusion injury. Br
APK cascades are involved in baicalin's neuroprotection against gerbil global ischemia/reperfusion injury. Br
Deprivation/Reoxygenation-Induced Injury by Modulating the BDNF-TrkB/PI3K/Akt and MAPK/Erk1/2 Signa
Deprivation/Reoxygenation-Induced Injury by Modulating the BDNF-TrkB/PI3K/Akt and MAPK/Erk1/2 Signa
Deprivation/Reoxygenation-Induced Injury by Modulating the BDNF-TrkB/PI3K/Akt and MAPK/Erk1/2 Signa
aicalin inhibiting cerebral ischemia/hypoxia-induced neuronal apoptosis via MRTF-A-mediated transactivity.
aicalin inhibiting cerebral ischemia/hypoxia-induced neuronal apoptosis via MRTF-A-mediated transactivity.
aicalin inhibiting cerebral ischemia/hypoxia-induced neuronal apoptosis via MRTF-A-mediated transactivity.
aicalin inhibiting cerebral ischemia/hypoxia-induced neuronal apoptosis via MRTF-A-mediated transactivity.
es ischemia-reperfusion-induced brain injury by reducing neuroinflammation and neuronal damage. Biome
es ischemia-reperfusion-induced brain injury by reducing neuroinflammation and neuronal damage. Biome
es ischemia-reperfusion-induced brain injury by reducing neuroinflammation and neuronal damage. Biome
es ischemia-reperfusion-induced brain injury by reducing neuroinflammation and neuronal damage. Biome
es ischemia-reperfusion-induced brain injury by reducing neuroinflammation and neuronal damage. Biome
es ischemia-reperfusion-induced brain injury by reducing neuroinflammation and neuronal damage. Biome
es ischemia-reperfusion-induced brain injury by reducing neuroinflammation and neuronal damage. Biome
es ischemia-reperfusion-induced brain injury by reducing neuroinflammation and neuronal damage. Biome
es ischemia-reperfusion-induced brain injury by reducing neuroinflammation and neuronal damage. Biome
led with improved behavioral function in cerebral ischemia. Neurochem Int. 2016 Jul;97:154-71. doi: 10.10
led with improved behavioral function in cerebral ischemia. Neurochem Int. 2016 Jul;97:154-71. doi: 10.10
led with improved behavioral function in cerebral ischemia. Neurochem Int. 2016 Jul;97:154-71. doi: 10.10
led with improved behavioral function in cerebral ischemia. Neurochem Int. 2016 Jul;97:154-71. doi: 10.10
Activated Microglia/Brain Macrophage in Cerebral Ischemia and in Activated BV-2 Microglia Through Regu
Activated Microglia/Brain Macrophage in Cerebral Ischemia and in Activated BV-2 Microglia Through Regu
Activated Microglia/Brain Macrophage in Cerebral Ischemia and in Activated BV-2 Microglia Through Regu
Activated Microglia/Brain Macrophage in Cerebral Ischemia and in Activated BV-2 Microglia Through Regu
Activated Microglia/Brain Macrophage in Cerebral Ischemia and in Activated BV-2 Microglia Through Regu
glial inflammatory activation. Neuroscience. 2011 Jun 30;185:150-60. doi: 10.1016/j.neuroscience.2011.04.
glial inflammatory activation. Neuroscience. 2011 Jun 30;185:150-60. doi: 10.1016/j.neuroscience.2011.04.
glial inflammatory activation. Neuroscience. 2011 Jun 30;185:150-60. doi: 10.1016/j.neuroscience.2011.04.
glial inflammatory activation. Neuroscience. 2011 Jun 30;185:150-60. doi: 10.1016/j.neuroscience.2011.04.
glial inflammatory activation. Neuroscience. 2011 Jun 30;185:150-60. doi: 10.1016/j.neuroscience.2011.04.
glial inflammatory activation. Neuroscience. 2011 Jun 30;185:150-60. doi: 10.1016/j.neuroscience.2011.04.
glial inflammatory activation. Neuroscience. 2011 Jun 30;185:150-60. doi: 10.1016/j.neuroscience.2011.04.
glial inflammatory activation. Neuroscience. 2011 Jun 30;185:150-60. doi: 10.1016/j.neuroscience.2011.04.

epeatedly cerebral ischemia-reperfusion in rats. Pharmacol Biochem Behav. 2014 Mar;118:51-9. doi: 10.10
epeatedly cerebral ischemia-reperfusion in rats. Pharmacol Biochem Behav. 2014 Mar;118:51-9. doi: 10.10
epeatedly cerebral ischemia-reperfusion in rats. Pharmacol Biochem Behav. 2014 Mar;118:51-9. doi: 10.10
epeatedly cerebral ischemia-reperfusion in rats. Pharmacol Biochem Behav. 2014 Mar;118:51-9. doi: 10.10
epeatedly cerebral ischemia-reperfusion in rats. Pharmacol Biochem Behav. 2014 Mar;118:51-9. doi: 10.10
lothelial permeability. Eur J Pharmacol. 2019 Jun 15;853:210-219. doi: 10.1016/j.ejphar.2019.03.015. Epub
lothelial permeability. Eur J Pharmacol. 2019 Jun 15;853:210-219. doi: 10.1016/j.ejphar.2019.03.015. Epub
lothelial permeability. Eur J Pharmacol. 2019 Jun 15;853:210-219. doi: 10.1016/j.ejphar.2019.03.015. Epub
stroke via mTOR/Ulk1 pathway. Biomed Pharmacother. 2018 Mar;99:583-590. doi: 10.1016/j.biopha.2018.
stroke via mTOR/Ulk1 pathway. Biomed Pharmacother. 2018 Mar;99:583-590. doi: 10.1016/j.biopha.2018.
stroke via mTOR/Ulk1 pathway. Biomed Pharmacother. 2018 Mar;99:583-590. doi: 10.1016/j.biopha.2018.
stroke via mTOR/Ulk1 pathway. Biomed Pharmacother. 2018 Mar;99:583-590. doi: 10.1016/j.biopha.2018.
stroke via mTOR/Ulk1 pathway. Biomed Pharmacother. 2018 Mar;99:583-590. doi: 10.1016/j.biopha.2018.
stroke via mTOR/Ulk1 pathway. Biomed Pharmacother. 2018 Mar;99:583-590. doi: 10.1016/j.biopha.2018.
stroke via mTOR/Ulk1 pathway. Biomed Pharmacother. 2018 Mar;99:583-590. doi: 10.1016/j.biopha.2018.
stroke via mTOR/Ulk1 pathway. Biomed Pharmacother. 2018 Mar;99:583-590. doi: 10.1016/j.biopha.2018.
stroke via mTOR/Ulk1 pathway. Biomed Pharmacother. 2018 Mar;99:583-590. doi: 10.1016/j.biopha.2018.
stroke via mTOR/Ulk1 pathway. Biomed Pharmacother. 2018 Mar;99:583-590. doi: 10.1016/j.biopha.2018.
stroke via mTOR/Ulk1 pathway. Biomed Pharmacother. 2018 Mar;99:583-590. doi: 10.1016/j.biopha.2018.
ia Caveolin-1/VEGF In Vitro and In Vivo. Oxid Med Cell Longev. 2018 Dec 3;2018:7017204. doi: 10.1155/20
ia Caveolin-1/VEGF In Vitro and In Vivo. Oxid Med Cell Longev. 2018 Dec 3;2018:7017204. doi: 10.1155/20
ia Caveolin-1/VEGF In Vitro and In Vivo. Oxid Med Cell Longev. 2018 Dec 3;2018:7017204. doi: 10.1155/20
ia Caveolin-1/VEGF In Vitro and In Vivo. Oxid Med Cell Longev. 2018 Dec 3;2018:7017204. doi: 10.1155/20
ia Caveolin-1/VEGF In Vitro and In Vivo. Oxid Med Cell Longev. 2018 Dec 3;2018:7017204. doi: 10.1155/20
ugh Histone Acetylation-Mediated Neurochemical Alterations. Med Sci Monit. 2017 Aug 19;23:4004-4013.
ugh Histone Acetylation-Mediated Neurochemical Alterations. Med Sci Monit. 2017 Aug 19;23:4004-4013.
ugh Histone Acetylation-Mediated Neurochemical Alterations. Med Sci Monit. 2017 Aug 19;23:4004-4013.
ugh Histone Acetylation-Mediated Neurochemical Alterations. Med Sci Monit. 2017 Aug 19;23:4004-4013.
ugh Histone Acetylation-Mediated Neurochemical Alterations. Med Sci Monit. 2017 Aug 19;23:4004-4013.
ats via nuclear factor-κB inhibition and peroxisome proliferator-activated receptor up-regulation. Neuroche
ats via nuclear factor-κB inhibition and peroxisome proliferator-activated receptor up-regulation. Neuroche
ats via nuclear factor-κB inhibition and peroxisome proliferator-activated receptor up-regulation. Neuroche
ough inhibition of inflammatory response mediated by NF-κB, PPARα and PPARγ in rats. Int Immunopharm
ough inhibition of inflammatory response mediated by NF-κB, PPARα and PPARγ in rats. Int Immunopharm
ough inhibition of inflammatory response mediated by NF-κB, PPARα and PPARγ in rats. Int Immunopharm
ough inhibition of inflammatory response mediated by NF-κB, PPARα and PPARγ in rats. Int Immunopharm
after OGD/R injury via suppressing IRE1α-XBP1 signaling pathway. Life Sci. 2020 Aug 15;255:117847. doi: 1
after OGD/R injury via suppressing IRE1α-XBP1 signaling pathway. Life Sci. 2020 Aug 15;255:117847. doi: 1
after OGD/R injury via suppressing IRE1α-XBP1 signaling pathway. Life Sci. 2020 Aug 15;255:117847. doi: 1
after OGD/R injury via suppressing IRE1α-XBP1 signaling pathway. Life Sci. 2020 Aug 15;255:117847. doi: 1
after OGD/R injury via suppressing IRE1α-XBP1 signaling pathway. Life Sci. 2020 Aug 15;255:117847. doi: 1
after OGD/R injury via suppressing IRE1α-XBP1 signaling pathway. Life Sci. 2020 Aug 15;255:117847. doi: 1
after OGD/R injury via suppressing IRE1α-XBP1 signaling pathway. Life Sci. 2020 Aug 15;255:117847. doi: 1
after OGD/R injury via suppressing IRE1α-XBP1 signaling pathway. Life Sci. 2020 Aug 15;255:117847. doi: 1
after OGD/R injury via suppressing IRE1α-XBP1 signaling pathway. Life Sci. 2020 Aug 15;255:117847. doi: 1
after OGD/R injury via suppressing IRE1α-XBP1 signaling pathway. Life Sci. 2020 Aug 15;255:117847. doi: 1

after OGD/R injury via suppressing IRE1α-XBP1 signaling pathway. Life Sci. 2020 Aug 15;255:117847. doi: 1

after OGD/R injury via suppressing IRE1α-XBP1 signaling pathway. Life Sci. 2020 Aug 15;255:117847. doi: 1

after OGD/R injury via suppressing IRE1α-XBP1 signaling pathway. Life Sci. 2020 Aug 15;255:117847. doi: 1

after OGD/R injury via suppressing IRE1α-XBP1 signaling pathway. Life Sci. 2020 Aug 15;255:117847. doi: 1

the activation of NF-κB in experimental ischemic stroke. Metab Brain Dis. 2021 Oct;36(7):1779-1790. doi: 1

the activation of NF-κB in experimental ischemic stroke. Metab Brain Dis. 2021 Oct;36(7):1779-1790. doi: 1

the activation of NF-κB in experimental ischemic stroke. Metab Brain Dis. 2021 Oct;36(7):1779-1790. doi: 1

the activation of NF-κB in experimental ischemic stroke. Metab Brain Dis. 2021 Oct;36(7):1779-1790. doi: 1

the activation of NF-κB in experimental ischemic stroke. Metab Brain Dis. 2021 Oct;36(7):1779-1790. doi: 1

the activation of NF-κB in experimental ischemic stroke. Metab Brain Dis. 2021 Oct;36(7):1779-1790. doi: 1

the activation of NF-κB in experimental ischemic stroke. Metab Brain Dis. 2021 Oct;36(7):1779-1790. doi: 1

the activation of NF-κB in experimental ischemic stroke. Metab Brain Dis. 2021 Oct;36(7):1779-1790. doi: 1

the activation of NF-κB in experimental ischemic stroke. Metab Brain Dis. 2021 Oct;36(7):1779-1790. doi: 1

the activation of NF-κB in experimental ischemic stroke. Metab Brain Dis. 2021 Oct;36(7):1779-1790. doi: 1

creases in the Neuronal Calcium Sensor Protein Hippocalcin. Neuroscience. 2020 Mar 15;430:47-62. doi: 10

creases in the Neuronal Calcium Sensor Protein Hippocalcin. Neuroscience. 2020 Mar 15;430:47-62. doi: 10

creases in the Neuronal Calcium Sensor Protein Hippocalcin. Neuroscience. 2020 Mar 15;430:47-62. doi: 10

/reperfusion injury via Nrf2-mediated inhibition of the NOX4/ROS/NF-κB pathway. Chem Biol Interact. 201

/reperfusion injury via Nrf2-mediated inhibition of the NOX4/ROS/NF-κB pathway. Chem Biol Interact. 201

/reperfusion injury via Nrf2-mediated inhibition of the NOX4/ROS/NF-κB pathway. Chem Biol Interact. 201

/reperfusion injury via Nrf2-mediated inhibition of the NOX4/ROS/NF-κB pathway. Chem Biol Interact. 201

/reperfusion injury via Nrf2-mediated inhibition of the NOX4/ROS/NF-κB pathway. Chem Biol Interact. 201

rfusion injury in rats. J Ethnopharmacol. 2012 Dec 18;144(3):768-74. doi: 10.1016/j.jep.2012.09.056. Epub

rfusion injury in rats. J Ethnopharmacol. 2012 Dec 18;144(3):768-74. doi: 10.1016/j.jep.2012.09.056. Epub

leurological Injury after Cerebral Ischemia by Reducing Accumulation of Hypertrophic and TNF-α-Containin

leurological Injury after Cerebral Ischemia by Reducing Accumulation of Hypertrophic and TNF-α-Containin

ia and Reperfusion Injury in Rats. Cell Physiol Biochem. 2018;45(2):537-546. doi: 10.1159/000487031. Epu

ia and Reperfusion Injury in Rats. Cell Physiol Biochem. 2018;45(2):537-546. doi: 10.1159/000487031. Epu

ia and Reperfusion Injury in Rats. Cell Physiol Biochem. 2018;45(2):537-546. doi: 10.1159/000487031. Epu

ia and Reperfusion Injury in Rats. Cell Physiol Biochem. 2018;45(2):537-546. doi: 10.1159/000487031. Epu

ia and Reperfusion Injury in Rats. Cell Physiol Biochem. 2018;45(2):537-546. doi: 10.1159/000487031. Epu

thohumol protects neuron from cerebral ischemia injury in experimental stroke. Mol Biol Rep. 2020 Apr;4

against kainic acid-induced excitotoxicity in rats. Neurochem Int. 2020 Feb;133:104629. doi: 10.1016/j.neu

against kainic acid-induced excitotoxicity in rats. Neurochem Int. 2020 Feb;133:104629. doi: 10.1016/j.neu

against kainic acid-induced excitotoxicity in rats. Neurochem Int. 2020 Feb;133:104629. doi: 10.1016/j.neu

against kainic acid-induced excitotoxicity in rats. Neurochem Int. 2020 Feb;133:104629. doi: 10.1016/j.neu

by attenuating inflammation and ferroptosis in rats. Int J Mol Med. 2021 Apr;47(4):52. doi: 10.3892/ijmm.2

by attenuating inflammation and ferroptosis in rats. Int J Mol Med. 2021 Apr;47(4):52. doi: 10.3892/ijmm.2

by attenuating inflammation and ferroptosis in rats. Int J Mol Med. 2021 Apr;47(4):52. doi: 10.3892/ijmm.2

by attenuating inflammation and ferroptosis in rats. Int J Mol Med. 2021 Apr;47(4):52. doi: 10.3892/ijmm.2

by attenuating inflammation and ferroptosis in rats. Int J Mol Med. 2021 Apr;47(4):52. doi: 10.3892/ijmm.2

by attenuating inflammation and ferroptosis in rats. Int J Mol Med. 2021 Apr;47(4):52. doi: 10.3892/ijmm.2

oxia-Induced Memory Impairment via Modulation of SIRT3 Signaling. Mol Neurobiol. 2016 Dec;53(10):720

oxia-Induced Memory Impairment via Modulation of SIRT3 Signaling. Mol Neurobiol. 2016 Dec;53(10):720

ent is involved in the anti-apoptotic effect of berberine against cerebral ischemia-reperfusion. Eur J Pharm

ent is involved in the anti-apoptotic effect of berberine against cerebral ischemia-reperfusion. Eur J Pharm

ent is involved in the anti-apoptotic effect of berberine against cerebral ischemia-reperfusion. Eur J Pharm
argeting inflammatory cytokines. J Chem Neuroanat. 2018 Jan;87:54-59. doi: 10.1016/j.jchemneu.2017.04.
argeting inflammatory cytokines. J Chem Neuroanat. 2018 Jan;87:54-59. doi: 10.1016/j.jchemneu.2017.04.
argeting inflammatory cytokines. J Chem Neuroanat. 2018 Jan;87:54-59. doi: 10.1016/j.jchemneu.2017.04.
argeting inflammatory cytokines. J Chem Neuroanat. 2018 Jan;87:54-59. doi: 10.1016/j.jchemneu.2017.04.
tivation following permanent cerebral ischemia in rats. Biochem Biophys Res Commun. 2015 Jul 31;463(3):
tivation following permanent cerebral ischemia in rats. Biochem Biophys Res Commun. 2015 Jul 31;463(3):
tivation following permanent cerebral ischemia in rats. Biochem Biophys Res Commun. 2015 Jul 31;463(3):
pyrazine in adult rat brain after focal ischemia. Neurol Res. 2010 Jun;32(5):547-55. doi: 10.1179/17431320
pyrazine in adult rat brain after focal ischemia. Neurol Res. 2010 Jun;32(5):547-55. doi: 10.1179/17431320
injury via activating PI3K/Akt pathway. Hum Exp Toxicol. 2019 Oct;38(10):1168-1177. doi: 10.1177/096032
injury via activating PI3K/Akt pathway. Hum Exp Toxicol. 2019 Oct;38(10):1168-1177. doi: 10.1177/096032
injury via activating PI3K/Akt pathway. Hum Exp Toxicol. 2019 Oct;38(10):1168-1177. doi: 10.1177/096032
injury via activating PI3K/Akt pathway. Hum Exp Toxicol. 2019 Oct;38(10):1168-1177. doi: 10.1177/096032
injury via activating PI3K/Akt pathway. Hum Exp Toxicol. 2019 Oct;38(10):1168-1177. doi: 10.1177/096032
l artery occlusion in rats: involvement of Bcl-2 family proteins. Am J Chin Med. 2010;38(2):307-18. doi: 10.1
l artery occlusion in rats: involvement of Bcl-2 family proteins. Am J Chin Med. 2010;38(2):307-18. doi: 10.1
l artery occlusion in rats: involvement of Bcl-2 family proteins. Am J Chin Med. 2010;38(2):307-18. doi: 10.1
l artery occlusion in rats: involvement of Bcl-2 family proteins. Am J Chin Med. 2010;38(2):307-18. doi: 10.1
duced by Cerebral Ischemia-Reperfusion Injury: Involvement of c-Abl Activation. J Mol Neurosci. 2018 Jul;6
duced by Cerebral Ischemia-Reperfusion Injury: Involvement of c-Abl Activation. J Mol Neurosci. 2018 Jul;6
duced by Cerebral Ischemia-Reperfusion Injury: Involvement of c-Abl Activation. J Mol Neurosci. 2018 Jul;6
duced by Cerebral Ischemia-Reperfusion Injury: Involvement of c-Abl Activation. J Mol Neurosci. 2018 Jul;6
vo-tetrahydropalmatine attenuates mouse blood-brain barrier injury induced by focal cerebral ischemia an
vo-tetrahydropalmatine attenuates mouse blood-brain barrier injury induced by focal cerebral ischemia an
vo-tetrahydropalmatine attenuates mouse blood-brain barrier injury induced by focal cerebral ischemia an
of nuclear factor erythroid 2-related factor 2 signalling in ischaemic stroke. Br J Pharmacol. 2019 Feb;176(
t Cerebral Ischemia-Reperfusion Via Mediating Autophagy and Inflammation. J Mol Neurosci. 2018 Jan;64(
t Cerebral Ischemia-Reperfusion Via Mediating Autophagy and Inflammation. J Mol Neurosci. 2018 Jan;64(
t Cerebral Ischemia-Reperfusion Via Mediating Autophagy and Inflammation. J Mol Neurosci. 2018 Jan;64(
t Cerebral Ischemia-Reperfusion Via Mediating Autophagy and Inflammation. J Mol Neurosci. 2018 Jan;64(
t Cerebral Ischemia-Reperfusion Via Mediating Autophagy and Inflammation. J Mol Neurosci. 2018 Jan;64(
ng pathway in rats. Int J Clin Exp Med. 2015 Sep 15;8(9):14985-91. PMID: 26628981; PMCID: PMC4658870.
ng pathway in rats. Int J Clin Exp Med. 2015 Sep 15;8(9):14985-91. PMID: 26628981; PMCID: PMC4658870.
ng pathway in rats. Int J Clin Exp Med. 2015 Sep 15;8(9):14985-91. PMID: 26628981; PMCID: PMC4658870.
ng pathway in rats. Int J Clin Exp Med. 2015 Sep 15;8(9):14985-91. PMID: 26628981; PMCID: PMC4658870.
ng pathway in rats. Int J Clin Exp Med. 2015 Sep 15;8(9):14985-91. PMID: 26628981; PMCID: PMC4658870.
lucose deprivation/reoxygenation injury through flotillin-1 and extracellular signal-regulated kinase1/2 pat
lucose deprivation/reoxygenation injury through flotillin-1 and extracellular signal-regulated kinase1/2 pat
lucose deprivation/reoxygenation injury through flotillin-1 and extracellular signal-regulated kinase1/2 pat
se brain with ischemic stroke. Brain Behav. 2018 Jan 22;8(2):e00921. doi: 10.1002/brb3.921. PMID: 294842
se brain with ischemic stroke. Brain Behav. 2018 Jan 22;8(2):e00921. doi: 10.1002/brb3.921. PMID: 294842
se brain with ischemic stroke. Brain Behav. 2018 Jan 22;8(2):e00921. doi: 10.1002/brb3.921. PMID: 294842
se brain with ischemic stroke. Brain Behav. 2018 Jan 22;8(2):e00921. doi: 10.1002/brb3.921. PMID: 294842
ocal function between autophagy and HIF-1α in cerebral ischemia-reperfusion injury. Drug Des Devel Ther.
ocal function between autophagy and HIF-1α in cerebral ischemia-reperfusion injury. Drug Des Devel Ther.

rague-Dawley rats and PC12 cells by suppressing overactivated autophagy. J Photochem Photobiol B. 2018
mia reperfusion injury via increase of mitochondrial biogenesis. Neurochem Res. 2014 Jul;39(7):1322-31. dc
mia reperfusion injury via increase of mitochondrial biogenesis. Neurochem Res. 2014 Jul;39(7):1322-31. dc
mia reperfusion injury via increase of mitochondrial biogenesis. Neurochem Res. 2014 Jul;39(7):1322-31. dc
mia reperfusion injury via increase of mitochondrial biogenesis. Neurochem Res. 2014 Jul;39(7):1322-31. dc
esponse in the rat hippocampus following global cerebral ischemia. Neurochem Int. 2012 Oct;61(5):659-65.
esponse in the rat hippocampus following global cerebral ischemia. Neurochem Int. 2012 Oct;61(5):659-65.
esponse in the rat hippocampus following global cerebral ischemia. Neurochem Int. 2012 Oct;61(5):659-65.
esponse in the rat hippocampus following global cerebral ischemia. Neurochem Int. 2012 Oct;61(5):659-65.
esponse in the rat hippocampus following global cerebral ischemia. Neurochem Int. 2012 Oct;61(5):659-65.
dysfunction by regulation of the MMP-9/TIMP-1 balance after cerebral ischemia reperfusion in rats. J Mol I
dysfunction by regulation of the MMP-9/TIMP-1 balance after cerebral ischemia reperfusion in rats. J Mol I
dysfunction by regulation of the MMP-9/TIMP-1 balance after cerebral ischemia reperfusion in rats. J Mol I
biting NLRP3 inflammasome activation through Sirt1-dependent autophagy induction. Int Immunopharmac
biting NLRP3 inflammasome activation through Sirt1-dependent autophagy induction. Int Immunopharmac
biting NLRP3 inflammasome activation through Sirt1-dependent autophagy induction. Int Immunopharmac
biting NLRP3 inflammasome activation through Sirt1-dependent autophagy induction. Int Immunopharmac
biting NLRP3 inflammasome activation through Sirt1-dependent autophagy induction. Int Immunopharmac
sient cerebral ischemia-reperfusion in mice through anti-inflammatory mechanism. Pharmacol Biochem Be
sient cerebral ischemia-reperfusion in mice through anti-inflammatory mechanism. Pharmacol Biochem Be
sient cerebral ischemia-reperfusion in mice through anti-inflammatory mechanism. Pharmacol Biochem Be
sient cerebral ischemia-reperfusion in mice through anti-inflammatory mechanism. Pharmacol Biochem Be
sient cerebral ischemia-reperfusion in mice through anti-inflammatory mechanism. Pharmacol Biochem Be
sient cerebral ischemia-reperfusion in mice through anti-inflammatory mechanism. Pharmacol Biochem Be
l 1;9(13):1275-82. doi: 10.4103/1673-5374.137574. PMID: 25221579; PMCID: PMC4160853.
l 1;9(13):1275-82. doi: 10.4103/1673-5374.137574. PMID: 25221579; PMCID: PMC4160853.
l 1;9(13):1275-82. doi: 10.4103/1673-5374.137574. PMID: 25221579; PMCID: PMC4160853.
glial polarization after cerebral ischemia. J Neuroinflammation. 2018 Feb 9;15(1):39. doi: 10.1186/s12974-(
glial polarization after cerebral ischemia. J Neuroinflammation. 2018 Feb 9;15(1):39. doi: 10.1186/s12974-(
glial polarization after cerebral ischemia. J Neuroinflammation. 2018 Feb 9;15(1):39. doi: 10.1186/s12974-(
glial polarization after cerebral ischemia. J Neuroinflammation. 2018 Feb 9;15(1):39. doi: 10.1186/s12974-(
glial polarization after cerebral ischemia. J Neuroinflammation. 2018 Feb 9;15(1):39. doi: 10.1186/s12974-(
glial polarization after cerebral ischemia. J Neuroinflammation. 2018 Feb 9;15(1):39. doi: 10.1186/s12974-(
glial polarization after cerebral ischemia. J Neuroinflammation. 2018 Feb 9;15(1):39. doi: 10.1186/s12974-(
glial polarization after cerebral ischemia. J Neuroinflammation. 2018 Feb 9;15(1):39. doi: 10.1186/s12974-(
glial polarization after cerebral ischemia. J Neuroinflammation. 2018 Feb 9;15(1):39. doi: 10.1186/s12974-(
glial polarization after cerebral ischemia. J Neuroinflammation. 2018 Feb 9;15(1):39. doi: 10.1186/s12974-(
glial polarization after cerebral ischemia. J Neuroinflammation. 2018 Feb 9;15(1):39. doi: 10.1186/s12974-(
glial polarization after cerebral ischemia. J Neuroinflammation. 2018 Feb 9;15(1):39. doi: 10.1186/s12974-(
bits NLRP3 inflammasome activation and apoptosis in microglia induced by cerebral ischemia/reperfusion i
bits NLRP3 inflammasome activation and apoptosis in microglia induced by cerebral ischemia/reperfusion i
bits NLRP3 inflammasome activation and apoptosis in microglia induced by cerebral ischemia/reperfusion i
bits NLRP3 inflammasome activation and apoptosis in microglia induced by cerebral ischemia/reperfusion i
After Permanent Middle Cerebral Artery Occlusion in Rats by Regulating PI3K/PKB/Nrf2/NFκB Signaling Ra
After Permanent Middle Cerebral Artery Occlusion in Rats by Regulating PI3K/PKB/Nrf2/NFκB Signaling Ra
After Permanent Middle Cerebral Artery Occlusion in Rats by Regulating PI3K/PKB/Nrf2/NFκB Signaling Ra

/ After Permanent Middle Cerebral Artery Occlusion in Rats by Regulating PI3K/PKB/Nrf2/NFκB Signaling Ra
autophagy in BV-2 microglial cells exposed to oxygen-glucose deprivation/reoxygenation. Int Immunophar
autophagy in BV-2 microglial cells exposed to oxygen-glucose deprivation/reoxygenation. Int Immunophar
autophagy in BV-2 microglial cells exposed to oxygen-glucose deprivation/reoxygenation. Int Immunophar
autophagy in BV-2 microglial cells exposed to oxygen-glucose deprivation/reoxygenation. Int Immunophar
autophagy in BV-2 microglial cells exposed to oxygen-glucose deprivation/reoxygenation. Int Immunophar
Jeurovascular Damage via GluN2A/AKT/ ERK-Dependent Mechanism. Cell Physiol Biochem. 2017;43(2):705-
ischemic long-term potentiation via GluN2A. Pak J Pharm Sci. 2021 May;34(3):909-914. PMID: 34602413.
ebral ischaemia/reperfusion injury in gerbils. J Pharm Pharmacol. 2014 Sep;66(9):1265-70. doi: 10.1111/jph
ebral ischaemia/reperfusion injury in gerbils. J Pharm Pharmacol. 2014 Sep;66(9):1265-70. doi: 10.1111/jph
ebral ischaemia/reperfusion injury in gerbils. J Pharm Pharmacol. 2014 Sep;66(9):1265-70. doi: 10.1111/jph
n Funct. 2010 Jul 9;6:43. doi: 10.1186/1744-9081-6-43. PMID: 20618938; PMCID: PMC2908551.
n Funct. 2010 Jul 9;6:43. doi: 10.1186/1744-9081-6-43. PMID: 20618938; PMCID: PMC2908551.
n Funct. 2010 Jul 9;6:43. doi: 10.1186/1744-9081-6-43. PMID: 20618938; PMCID: PMC2908551.
ia. Int J Mol Sci. 2010 Nov 16;11(11):4580-90. doi: 10.3390/ijms11114580. PMID: 21151457; PMCID: PMC3(
erebral Ischemic Injury in Rats. J Mol Neurosci. 2015 Nov;57(3):335-51. doi: 10.1007/s12031-015-0623-5. E
erebral Ischemic Injury in Rats. J Mol Neurosci. 2015 Nov;57(3):335-51. doi: 10.1007/s12031-015-0623-5. E
erebral Ischemic Injury in Rats. J Mol Neurosci. 2015 Nov;57(3):335-51. doi: 10.1007/s12031-015-0623-5. E
erebral Ischemic Injury in Rats. J Mol Neurosci. 2015 Nov;57(3):335-51. doi: 10.1007/s12031-015-0623-5. E
erebral Ischemic Injury in Rats. J Mol Neurosci. 2015 Nov;57(3):335-51. doi: 10.1007/s12031-015-0623-5. E
erebral Ischemic Injury in Rats. J Mol Neurosci. 2015 Nov;57(3):335-51. doi: 10.1007/s12031-015-0623-5. E
erebral Ischemic Injury in Rats. J Mol Neurosci. 2015 Nov;57(3):335-51. doi: 10.1007/s12031-015-0623-5. E
erebral Ischemic Injury in Rats. J Mol Neurosci. 2015 Nov;57(3):335-51. doi: 10.1007/s12031-015-0623-5. E
erebral Ischemic Injury in Rats. J Mol Neurosci. 2015 Nov;57(3):335-51. doi: 10.1007/s12031-015-0623-5. E
erebral Ischemic Injury in Rats. J Mol Neurosci. 2015 Nov;57(3):335-51. doi: 10.1007/s12031-015-0623-5. E
Cytochrome C Signal Pathway Following Ischemia Reperfusion Injury in Rats. J Mol Neurosci. 2017 Feb;61(2
Cytochrome C Signal Pathway Following Ischemia Reperfusion Injury in Rats. J Mol Neurosci. 2017 Feb;61(2
Cytochrome C Signal Pathway Following Ischemia Reperfusion Injury in Rats. J Mol Neurosci. 2017 Feb;61(2
Cytochrome C Signal Pathway Following Ischemia Reperfusion Injury in Rats. J Mol Neurosci. 2017 Feb;61(2
bral ischemia/reperfusion injury rats]. Zhongguo Zhong Xi Yi Jie He Za Zhi. 2013 Feb;33(2):229-34. Chinese.
bral ischemia/reperfusion injury rats]. Zhongguo Zhong Xi Yi Jie He Za Zhi. 2013 Feb;33(2):229-34. Chinese.
bral ischemia/reperfusion injury rats]. Zhongguo Zhong Xi Yi Jie He Za Zhi. 2013 Feb;33(2):229-34. Chinese.
on volume and IL-1 beta in the brain tissue and sera of focal cerebral ischemia/reperfusion injury model rats
ia-reperfusion injury in rats. J Orthop Surg Res. 2019 Aug 14;14(1):259. doi: 10.1186/s13018-019-1299-2. P
ia-reperfusion injury in rats. J Orthop Surg Res. 2019 Aug 14;14(1):259. doi: 10.1186/s13018-019-1299-2. P
ia-reperfusion injury in rats. J Orthop Surg Res. 2019 Aug 14;14(1):259. doi: 10.1186/s13018-019-1299-2. P
chemia/reperfusion injury correlating to suppression of neutrophils adhesion-related molecules. Neurocher
chemia/reperfusion injury correlating to suppression of neutrophils adhesion-related molecules. Neurocher
al ischemia and reperfusion in mice via anti-inflammatory mechanisms. Neurosci Lett. 2017 Feb 3;639:114-
ıpoptosis by inhibiting the activation of key factors in death receptor pathway and mitochondrial pathway.
ıpoptosis by inhibiting the activation of key factors in death receptor pathway and mitochondrial pathway.
ıpoptosis by inhibiting the activation of key factors in death receptor pathway and mitochondrial pathway.
ıpoptosis by inhibiting the activation of key factors in death receptor pathway and mitochondrial pathway.
ıpoptosis by inhibiting the activation of key factors in death receptor pathway and mitochondrial pathway.
ıpoptosis by inhibiting the activation of key factors in death receptor pathway and mitochondrial pathway.
ıpoptosis by inhibiting the activation of key factors in death receptor pathway and mitochondrial pathway.

poptosis by inhibiting the activation of key factors in death receptor pathway and mitochondrial pathway.
poptosis by inhibiting the activation of key factors in death receptor pathway and mitochondrial pathway.
LRP3 Inflammasome-Mediated Pyroptosis Inhibition via Activating Nrf2. Oxid Med Cell Longev. 2021 Dec 3
LRP3 Inflammasome-Mediated Pyroptosis Inhibition via Activating Nrf2. Oxid Med Cell Longev. 2021 Dec 3
LRP3 Inflammasome-Mediated Pyroptosis Inhibition via Activating Nrf2. Oxid Med Cell Longev. 2021 Dec 3
LRP3 Inflammasome-Mediated Pyroptosis Inhibition via Activating Nrf2. Oxid Med Cell Longev. 2021 Dec 3
LRP3 Inflammasome-Mediated Pyroptosis Inhibition via Activating Nrf2. Oxid Med Cell Longev. 2021 Dec 3
LRP3 Inflammasome-Mediated Pyroptosis Inhibition via Activating Nrf2. Oxid Med Cell Longev. 2021 Dec 3
al cerebral ischemia reperfusion injury in rats. Int J Biol Macromol. 2014 Feb;63:177-80. doi: 10.1016/j.ijbic
al cerebral ischemia reperfusion injury in rats. Int J Biol Macromol. 2014 Feb;63:177-80. doi: 10.1016/j.ijbic
al cerebral ischemia reperfusion injury in rats. Int J Biol Macromol. 2014 Feb;63:177-80. doi: 10.1016/j.ijbic
al cerebral ischemia reperfusion injury in rats. Int J Biol Macromol. 2014 Feb;63:177-80. doi: 10.1016/j.ijbic
iNOS expression protecting mice against hypoxia/ischemia-induced oxidative brain injury. Planta Med. 20
iNOS expression protecting mice against hypoxia/ischemia-induced oxidative brain injury. Planta Med. 20
holide from Andrographis paniculata on cerebral ischemia/reperfusion-induced brain injury through induc
olide stimulates p38 mitogen-activated protein kinase-nuclear factor erythroid-2-related factor 2-heme ox
rfusion injury in mice. Int J Mol Med. 2021 Oct;48(4):186. doi: 10.3892/ijmm.2021.5019. Epub 2021 Aug 9.
rfusion injury in mice. Int J Mol Med. 2021 Oct;48(4):186. doi: 10.3892/ijmm.2021.5019. Epub 2021 Aug 9.
rfusion injury in mice. Int J Mol Med. 2021 Oct;48(4):186. doi: 10.3892/ijmm.2021.5019. Epub 2021 Aug 9.
rfusion injury in mice. Int J Mol Med. 2021 Oct;48(4):186. doi: 10.3892/ijmm.2021.5019. Epub 2021 Aug 9.
rfusion injury in mice. Int J Mol Med. 2021 Oct;48(4):186. doi: 10.3892/ijmm.2021.5019. Epub 2021 Aug 9.
ion in rats]. Zhong Yao Cai. 2010 Apr;33(4):578-80. Chinese. PMID: 20845788.
ion in rats]. Zhong Yao Cai. 2010 Apr;33(4):578-80. Chinese. PMID: 20845788.
ion in rats]. Zhong Yao Cai. 2010 Apr;33(4):578-80. Chinese. PMID: 20845788.
ion in rats]. Zhong Yao Cai. 2010 Apr;33(4):578-80. Chinese. PMID: 20845788.
olides and bilobalide against cerebral ischemia injury by activating the Akt/Nrf2 pathway in vitro and in viv
olides and bilobalide against cerebral ischemia injury by activating the Akt/Nrf2 pathway in vitro and in viv
olides and bilobalide against cerebral ischemia injury by activating the Akt/Nrf2 pathway in vitro and in viv
olides and bilobalide against cerebral ischemia injury by activating the Akt/Nrf2 pathway in vitro and in viv
olides and bilobalide against cerebral ischemia injury by activating the Akt/Nrf2 pathway in vitro and in viv
olides and bilobalide against cerebral ischemia injury by activating the Akt/Nrf2 pathway in vitro and in viv
olides and bilobalide against cerebral ischemia injury by activating the Akt/Nrf2 pathway in vitro and in viv
olides and bilobalide against cerebral ischemia injury by activating the Akt/Nrf2 pathway in vitro and in viv
t cerebral ischemia/reperfusion damage in rats by activating Nrf2 and CREB through PI3K/Akt signaling. Act
t cerebral ischemia/reperfusion damage in rats by activating Nrf2 and CREB through PI3K/Akt signaling. Act
t cerebral ischemia/reperfusion damage in rats by activating Nrf2 and CREB through PI3K/Akt signaling. Act
erebral ischemia/reperfusion induced inflammatory response in astrocytes via TLR4/NF-κB pathway in rats.
erebral ischemia/reperfusion induced inflammatory response in astrocytes via TLR4/NF-κB pathway in rats.
erebral ischemia/reperfusion induced inflammatory response in astrocytes via TLR4/NF-κB pathway in rats.
erebral ischemia/reperfusion induced inflammatory response in astrocytes via TLR4/NF-κB pathway in rats.
H) Ameliorates Cerebral Ischemia Via Suppressing Neuronal Apoptosis. J Stroke Cerebrovasc Dis. 2021 Sep;
H) Ameliorates Cerebral Ischemia Via Suppressing Neuronal Apoptosis. J Stroke Cerebrovasc Dis. 2021 Sep;
H) Ameliorates Cerebral Ischemia Via Suppressing Neuronal Apoptosis. J Stroke Cerebrovasc Dis. 2021 Sep;
lucose deprivation/reperfusion: involvement of anti-oxidation and anti-inflammation through nuclear trans
lucose deprivation/reperfusion: involvement of anti-oxidation and anti-inflammation through nuclear trans
lucose deprivation/reperfusion: involvement of anti-oxidation and anti-inflammation through nuclear trans

lucose deprivation/reperfusion: involvement of anti-oxidation and anti-inflammation through nuclear trans
thetic borneol in cerebral ischaemia via modulation of the neurovascular unit. Biomed Pharmacother. 2018
thetic borneol in cerebral ischaemia via modulation of the neurovascular unit. Biomed Pharmacother. 2018
thetic borneol in cerebral ischaemia via modulation of the neurovascular unit. Biomed Pharmacother. 2018
thetic borneol in cerebral ischaemia via modulation of the neurovascular unit. Biomed Pharmacother. 2018
thetic borneol in cerebral ischaemia via modulation of the neurovascular unit. Biomed Pharmacother. 2018
thetic borneol in cerebral ischaemia via modulation of the neurovascular unit. Biomed Pharmacother. 2018
thetic borneol in cerebral ischaemia via modulation of the neurovascular unit. Biomed Pharmacother. 2018
gulating the mitochondrial calcium uniporter-induced apoptosis cascade in pMCAO rats. J Pharm Pharmacol
gulating the mitochondrial calcium uniporter-induced apoptosis cascade in pMCAO rats. J Pharm Pharmacol
gulating the mitochondrial calcium uniporter-induced apoptosis cascade in pMCAO rats. J Pharm Pharmacol
gulating the mitochondrial calcium uniporter-induced apoptosis cascade in pMCAO rats. J Pharm Pharmacol
gulating the mitochondrial calcium uniporter-induced apoptosis cascade in pMCAO rats. J Pharm Pharmacol
gulating the mitochondrial calcium uniporter-induced apoptosis cascade in pMCAO rats. J Pharm Pharmacol
gulating the mitochondrial calcium uniporter-induced apoptosis cascade in pMCAO rats. J Pharm Pharmacol
gulating the mitochondrial calcium uniporter-induced apoptosis cascade in pMCAO rats. J Pharm Pharmacol
ria lobate Radix in a Stroke Model Via the Akt/mTOR/BDNF Channel. Front Pharmacol. 2022 Jan 14;12:7724
ria lobate Radix in a Stroke Model Via the Akt/mTOR/BDNF Channel. Front Pharmacol. 2022 Jan 14;12:7724
ria lobate Radix in a Stroke Model Via the Akt/mTOR/BDNF Channel. Front Pharmacol. 2022 Jan 14;12:7724
ria lobate Radix in a Stroke Model Via the Akt/mTOR/BDNF Channel. Front Pharmacol. 2022 Jan 14;12:7724
s oxidative toxicity in HT22 cells and deficits in photothrombotic ischemia. J Ethnopharmacol. 2016 Jul 21;1
s oxidative toxicity in HT22 cells and deficits in photothrombotic ischemia. J Ethnopharmacol. 2016 Jul 21;1
s oxidative toxicity in HT22 cells and deficits in photothrombotic ischemia. J Ethnopharmacol. 2016 Jul 21;1
modin against Ischemia/Reperfusion Injury through Activating ERK-1/2 Signaling Pathway. Int J Mol Sci. 202
modin against Ischemia/Reperfusion Injury through Activating ERK-1/2 Signaling Pathway. Int J Mol Sci. 202
modin against Ischemia/Reperfusion Injury through Activating ERK-1/2 Signaling Pathway. Int J Mol Sci. 202
modin against Ischemia/Reperfusion Injury through Activating ERK-1/2 Signaling Pathway. Int J Mol Sci. 202
fusion injury in vivo and in vitro. Eur J Pharmacol. 2011 Aug 16;664(1-3):20-8. doi: 10.1016/j.ejphar.2011.04
fusion injury in vivo and in vitro. Eur J Pharmacol. 2011 Aug 16;664(1-3):20-8. doi: 10.1016/j.ejphar.2011.04
fusion injury in vivo and in vitro. Eur J Pharmacol. 2011 Aug 16;664(1-3):20-8. doi: 10.1016/j.ejphar.2011.04
fusion injury in vivo and in vitro. Eur J Pharmacol. 2011 Aug 16;664(1-3):20-8. doi: 10.1016/j.ejphar.2011.04
fusion injury in vivo and in vitro. Eur J Pharmacol. 2011 Aug 16;664(1-3):20-8. doi: 10.1016/j.ejphar.2011.04
totoxicity by modulating adenosine A1 receptors. Eur J Pharmacol. 2019 Jun 15;853:325-335. doi: 10.1016/j
uroprotection via inhibiting neutrophil infiltration and neuroinflammation after traumatic brain injury. J Neu
uroprotection via inhibiting neutrophil infiltration and neuroinflammation after traumatic brain injury. J Neu
uroprotection via inhibiting neutrophil infiltration and neuroinflammation after traumatic brain injury. J Neu
uroprotection via inhibiting neutrophil infiltration and neuroinflammation after traumatic brain injury. J Neu
uroprotection via inhibiting neutrophil infiltration and neuroinflammation after traumatic brain injury. J Neu
uroprotection via inhibiting neutrophil infiltration and neuroinflammation after traumatic brain injury. J Neu
APK Signaling Pathway. Biomol Ther (Seoul). 2017 May 1;25(3):272-278. doi: 10.4062/biomolther.2016.098
APK Signaling Pathway. Biomol Ther (Seoul). 2017 May 1;25(3):272-278. doi: 10.4062/biomolther.2016.098
APK Signaling Pathway. Biomol Ther (Seoul). 2017 May 1;25(3):272-278. doi: 10.4062/biomolther.2016.098
APK Signaling Pathway. Biomol Ther (Seoul). 2017 May 1;25(3):272-278. doi: 10.4062/biomolther.2016.098
APK Signaling Pathway. Biomol Ther (Seoul). 2017 May 1;25(3):272-278. doi: 10.4062/biomolther.2016.098
APK Signaling Pathway. Biomol Ther (Seoul). 2017 May 1;25(3):272-278. doi: 10.4062/biomolther.2016.098
APK Signaling Pathway. Biomol Ther (Seoul). 2017 May 1;25(3):272-278. doi: 10.4062/biomolther.2016.098

IAPK Signaling Pathway. Biomol Ther (Seoul). 2017 May 1;25(3):272-278. doi: 10.4062/biomolther.2016.09[illegible]
IAPK Signaling Pathway. Biomol Ther (Seoul). 2017 May 1;25(3):272-278. doi: 10.4062/biomolther.2016.09[illegible]
IAPK Signaling Pathway. Biomol Ther (Seoul). 2017 May 1;25(3):272-278. doi: 10.4062/biomolther.2016.09[illegible]
rk JH. Antioxidant Properties of Fucoidan Alleviate Acceleration and Exacerbation of Hippocampal Neurona
rk JH. Antioxidant Properties of Fucoidan Alleviate Acceleration and Exacerbation of Hippocampal Neurona
rk JH. Antioxidant Properties of Fucoidan Alleviate Acceleration and Exacerbation of Hippocampal Neurona
rk JH. Antioxidant Properties of Fucoidan Alleviate Acceleration and Exacerbation of Hippocampal Neurona
rk JH. Antioxidant Properties of Fucoidan Alleviate Acceleration and Exacerbation of Hippocampal Neurona
Pretreated fucoidan confers neuroprotection against transient global cerebral ischemic injury in the gerbil h
Pretreated fucoidan confers neuroprotection against transient global cerebral ischemic injury in the gerbil h
Pretreated fucoidan confers neuroprotection against transient global cerebral ischemic injury in the gerbil h
Pretreated fucoidan confers neuroprotection against transient global cerebral ischemic injury in the gerbil h
Pretreated fucoidan confers neuroprotection against transient global cerebral ischemic injury in the gerbil h
iol Confers Neuroprotection in Rats in a Model of Transient Global Cerebral Ischemia: Impact of Hippocamp
iol Confers Neuroprotection in Rats in a Model of Transient Global Cerebral Ischemia: Impact of Hippocamp
istration after hypoxia-ischemia to newborn rats reduces long-term brain injury and restores neurobehavic
abidiol against hippocampal cell death and cognitive impairment induced by bilateral common carotid arte
ion and promotes neuroplasticity and functional recovery after brain ischemia. Prog Neuropsychopharmac
ion and promotes neuroplasticity and functional recovery after brain ischemia. Prog Neuropsychopharmac
ion and promotes neuroplasticity and functional recovery after brain ischemia. Prog Neuropsychopharmac
ion and promotes neuroplasticity and functional recovery after brain ischemia. Prog Neuropsychopharmac
il in Cinnamon Powder, Ameliorates Cerebral Ischemia-Induced Brain Injury via Inhibition of Neuroinflamm
il in Cinnamon Powder, Ameliorates Cerebral Ischemia-Induced Brain Injury via Inhibition of Neuroinflamm
il in Cinnamon Powder, Ameliorates Cerebral Ischemia-Induced Brain Injury via Inhibition of Neuroinflamm
il in Cinnamon Powder, Ameliorates Cerebral Ischemia-Induced Brain Injury via Inhibition of Neuroinflamm
il in Cinnamon Powder, Ameliorates Cerebral Ischemia-Induced Brain Injury via Inhibition of Neuroinflamm
il in Cinnamon Powder, Ameliorates Cerebral Ischemia-Induced Brain Injury via Inhibition of Neuroinflamm
il in Cinnamon Powder, Ameliorates Cerebral Ischemia-Induced Brain Injury via Inhibition of Neuroinflamm
il in Cinnamon Powder, Ameliorates Cerebral Ischemia-Induced Brain Injury via Inhibition of Neuroinflamm
il in Cinnamon Powder, Ameliorates Cerebral Ischemia-Induced Brain Injury via Inhibition of Neuroinflamm
il in Cinnamon Powder, Ameliorates Cerebral Ischemia-Induced Brain Injury via Inhibition of Neuroinflamm

5;85(6):396-402. doi: 10.1016/j.brainresbull.2011.05.002. Epub 2011 May 11. PMID: 21600966.
5;85(6):396-402. doi: 10.1016/j.brainresbull.2011.05.002. Epub 2011 May 11. PMID: 21600966.
5;85(6):396-402. doi: 10.1016/j.brainresbull.2011.05.002. Epub 2011 May 11. PMID: 21600966.
5;85(6):396-402. doi: 10.1016/j.brainresbull.2011.05.002. Epub 2011 May 11. PMID: 21600966.
103. doi: 10.1016/j.brainres.2016.03.019. Epub 2016 Mar 23. PMID: 27016057.
ain Res Bull. 2013 Jan;90:1-9. doi: 10.1016/j.brainresbull.2012.09.014. Epub 2012 Oct 2. PMID: 230
ain Res Bull. 2013 Jan;90:1-9. doi: 10.1016/j.brainresbull.2012.09.014. Epub 2012 Oct 2. PMID: 230
ain Res Bull. 2013 Jan;90:1-9. doi: 10.1016/j.brainresbull.2012.09.014. Epub 2012 Oct 2. PMID: 230
ain Res Bull. 2013 Jan;90:1-9. doi: 10.1016/j.brainresbull.2012.09.014. Epub 2012 Oct 2. PMID: 230
ain Res Bull. 2013 Jan;90:1-9. doi: 10.1016/j.brainresbull.2012.09.014. Epub 2012 Oct 2. PMID: 230
ain Res Bull. 2013 Jan;90:1-9. doi: 10.1016/j.brainresbull.2012.09.014. Epub 2012 Oct 2. PMID: 230
ain Res Bull. 2013 Jan;90:1-9. doi: 10.1016/j.brainresbull.2012.09.014. Epub 2012 Oct 2. PMID: 230
ain Res Bull. 2013 Jan;90:1-9. doi: 10.1016/j.brainresbull.2012.09.014. Epub 2012 Oct 2. PMID: 230
aling Axes in Neuron-Astrocyte Cocultures. Front Pharmacol. 2021 Jun 21;12:599543. doi: 10.3389/
aling Axes in Neuron-Astrocyte Cocultures. Front Pharmacol. 2021 Jun 21;12:599543. doi: 10.3389/
aling Axes in Neuron-Astrocyte Cocultures. Front Pharmacol. 2021 Jun 21;12:599543. doi: 10.3389/
Eur J Pharmacol. 2015 Nov 15;767:201-10. doi: 10.1016/j.ejphar.2015.10.027. Epub 2015 Oct 17. P
Eur J Pharmacol. 2015 Nov 15;767:201-10. doi: 10.1016/j.ejphar.2015.10.027. Epub 2015 Oct 17. P
Eur J Pharmacol. 2015 Nov 15;767:201-10. doi: 10.1016/j.ejphar.2015.10.027. Epub 2015 Oct 17. P
Eur J Pharmacol. 2015 Nov 15;767:201-10. doi: 10.1016/j.ejphar.2015.10.027. Epub 2015 Oct 17. P
d Pharmacother. 2019 Sep;117:109102. doi: 10.1016/j.biopha.2019.109102. Epub 2019 Jun 19. PM
d Pharmacother. 2019 Sep;117:109102. doi: 10.1016/j.biopha.2019.109102. Epub 2019 Jun 19. PM
d Pharmacother. 2019 Sep;117:109102. doi: 10.1016/j.biopha.2019.109102. Epub 2019 Jun 19. PM
d Pharmacother. 2019 Sep;117:109102. doi: 10.1016/j.biopha.2019.109102. Epub 2019 Jun 19. PM
d Pharmacother. 2019 Sep;117:109102. doi: 10.1016/j.biopha.2019.109102. Epub 2019 Jun 19. PM
d Pharmacother. 2019 Sep;117:109102. doi: 10.1016/j.biopha.2019.109102. Epub 2019 Jun 19. PM
d Pharmacother. 2019 Sep;117:109102. doi: 10.1016/j.biopha.2019.109102. Epub 2019 Jun 19. PM
d Pharmacother. 2019 Sep;117:109102. doi: 10.1016/j.biopha.2019.109102. Epub 2019 Jun 19. PM
d Pharmacother. 2019 Sep;117:109102. doi: 10.1016/j.biopha.2019.109102. Epub 2019 Jun 19. PM
16/j.neuint.2016.04.007. Epub 2016 Apr 20. PMID: 27105682.
16/j.neuint.2016.04.007. Epub 2016 Apr 20. PMID: 27105682.
16/j.neuint.2016.04.007. Epub 2016 Apr 20. PMID: 27105682.
16/j.neuint.2016.04.007. Epub 2016 Apr 20. PMID: 27105682.
ulation of MAPKs Signaling Pathway. Neuromolecular Med. 2020 Jun;22(2):264-277. doi: 10.1007/s1
ulation of MAPKs Signaling Pathway. Neuromolecular Med. 2020 Jun;22(2):264-277. doi: 10.1007/s1
ulation of MAPKs Signaling Pathway. Neuromolecular Med. 2020 Jun;22(2):264-277. doi: 10.1007/s1
ulation of MAPKs Signaling Pathway. Neuromolecular Med. 2020 Jun;22(2):264-277. doi: 10.1007/s1
ulation of MAPKs Signaling Pathway. Neuromolecular Med. 2020 Jun;22(2):264-277. doi: 10.1007/s1
005. Epub 2011 Apr 19. PMID: 21524691.
005. Epub 2011 Apr 19. PMID: 21524691.
005. Epub 2011 Apr 19. PMID: 21524691.
005. Epub 2011 Apr 19. PMID: 21524691.
005. Epub 2011 Apr 19. PMID: 21524691.
005. Epub 2011 Apr 19. PMID: 21524691.
005. Epub 2011 Apr 19. PMID: 21524691.
005. Epub 2011 Apr 19. PMID: 21524691.

16/j.pbb.2014.01.003. Epub 2014 Jan 11. PMID: 24423938.
16/j.pbb.2014.01.003. Epub 2014 Jan 11. PMID: 24423938.
16/j.pbb.2014.01.003. Epub 2014 Jan 11. PMID: 24423938.
16/j.pbb.2014.01.003. Epub 2014 Jan 11. PMID: 24423938.
16/j.pbb.2014.01.003. Epub 2014 Jan 11. PMID: 24423938.
2019 Mar 12. PMID: 30876978
2019 Mar 12. PMID: 30876978
2019 Mar 12. PMID: 30876979
01.067. Epub 2018 Feb 20. PMID: 29710456.
01.067. Epub 2018 Feb 20. PMID: 29710456.
01.067. Epub 2018 Feb 20. PMID: 29710456.
01.067. Epub 2018 Feb 20. PMID: 29710456.
01.067. Epub 2018 Feb 20. PMID: 29710456.
01.067. Epub 2018 Feb 20. PMID: 29710456.
01.067. Epub 2018 Feb 20. PMID: 29710456.
01.067. Epub 2018 Feb 20. PMID: 29710456.
01.067. Epub 2018 Feb 20. PMID: 29710456.
01.067. Epub 2018 Feb 20. PMID: 29710456.
01.067. Epub 2018 Feb 20. PMID: 29710456.
18/7017204. PMID: 30622670; PMCID: PMC6304859.
18/7017204. PMID: 30622670; PMCID: PMC6304859.
18/7017204. PMID: 30622670; PMCID: PMC6304859.
18/7017204. PMID: 30622670; PMCID: PMC6304859.
18/7017204. PMID: 30622670; PMCID: PMC6304859.
doi: 10.12659/msm.902770. PMID: 28821706; PMCID: PMC5572783.
doi: 10.12659/msm.902770. PMID: 28821706; PMCID: PMC5572783.
doi: 10.12659/msm.902770. PMID: 28821706; PMCID: PMC5572783.
doi: 10.12659/msm.902770. PMID: 28821706; PMCID: PMC5572783.
doi: 10.12659/msm.902770. PMID: 28821706; PMCID: PMC5572783.
em Int. 2016 Jun;96:56-61. doi: 10.1016/j.neuint.2016.02.015. Epub 2016 Mar 3. PMID: 26939761.
em Int. 2016 Jun;96:56-61. doi: 10.1016/j.neuint.2016.02.015. Epub 2016 Mar 3. PMID: 26939761.
em Int. 2016 Jun;96:56-61. doi: 10.1016/j.neuint.2016.02.015. Epub 2016 Mar 3. PMID: 26939761.
acol. 2016 Jan;30:157-162. doi: 10.1016/j.intimp.2015.11.035. Epub 2015 Dec 8. PMID: 26679678.
acol. 2016 Jan;30:157-162. doi: 10.1016/j.intimp.2015.11.035. Epub 2015 Dec 8. PMID: 26679678.
acol. 2016 Jan;30:157-162. doi: 10.1016/j.intimp.2015.11.035. Epub 2015 Dec 8. PMID: 26679678.
acol. 2016 Jan;30:157-162. doi: 10.1016/j.intimp.2015.11.035. Epub 2015 Dec 8. PMID: 26679678.
0.1016/j.lfs.2020.117847. Epub 2020 May 27. PMID: 32470450.
0.1016/j.lfs.2020.117847. Epub 2020 May 27. PMID: 32470450.
0.1016/j.lfs.2020.117847. Epub 2020 May 27. PMID: 32470450.
0.1016/j.lfs.2020.117847. Epub 2020 May 27. PMID: 32470450.
0.1016/j.lfs.2020.117847. Epub 2020 May 27. PMID: 32470450.
0.1016/j.lfs.2020.117847. Epub 2020 May 27. PMID: 32470450.
0.1016/j.lfs.2020.117847. Epub 2020 May 27. PMID: 32470450.
0.1016/j.lfs.2020.117847. Epub 2020 May 27. PMID: 32470450.
0.1016/j.lfs.2020.117847. Epub 2020 May 27. PMID: 32470450.
0.1016/j.lfs.2020.117847. Epub 2020 May 27. PMID: 32470450.

).1016/j.lfs.2020.117847. Epub 2020 May 27. PMID: 32470450.
).1016/j.lfs.2020.117847. Epub 2020 May 27. PMID: 32470450.
).1016/j.lfs.2020.117847. Epub 2020 May 27. PMID: 32470450.
).1016/j.lfs.2020.117847. Epub 2020 May 27. PMID: 32470450.
0.1007/s11011-021-00731-6. Epub 2021 May 12. PMID: 33978900.
0.1007/s11011-021-00731-6. Epub 2021 May 12. PMID: 33978900.
0.1007/s11011-021-00731-6. Epub 2021 May 12. PMID: 33978900.
0.1007/s11011-021-00731-6. Epub 2021 May 12. PMID: 33978900.
0.1007/s11011-021-00731-6. Epub 2021 May 12. PMID: 33978900.
0.1007/s11011-021-00731-6. Epub 2021 May 12. PMID: 33978900.
0.1007/s11011-021-00731-6. Epub 2021 May 12. PMID: 33978900.
0.1007/s11011-021-00731-6. Epub 2021 May 12. PMID: 33978900.
0.1007/s11011-021-00731-6. Epub 2021 May 12. PMID: 33978900.
0.1007/s11011-021-00731-6. Epub 2021 May 12. PMID: 33978900.
.1016/j.neuroscience.2020.01.015. Epub 2020 Jan 23. PMID: 31982469.
.1016/j.neuroscience.2020.01.015. Epub 2020 Jan 23. PMID: 31982469.
.1016/j.neuroscience.2020.01.015. Epub 2020 Jan 23. PMID: 31982469.
3 Mar 25;284:32-40. doi: 10.1016/j.cbi.2018.02.017. Epub 2018 Feb 16. PMID: 29454613.
3 Mar 25;284:32-40. doi: 10.1016/j.cbi.2018.02.017. Epub 2018 Feb 16. PMID: 29454613.
3 Mar 25;284:32-40. doi: 10.1016/j.cbi.2018.02.017. Epub 2018 Feb 16. PMID: 29454613.
3 Mar 25;284:32-40. doi: 10.1016/j.cbi.2018.02.017. Epub 2018 Feb 16. PMID: 29454613.
3 Mar 25;284:32-40. doi: 10.1016/j.cbi.2018.02.017. Epub 2018 Feb 16. PMID: 29454613.
2012 Oct 30. PMID: 23123262.
2012 Oct 30. PMID: 23123262.
g Microglia in Rats. J Neuroimmune Pharmacol. 2020 Jun;15(2):326-339. doi: 10.1007/s11481-019-0
g Microglia in Rats. J Neuroimmune Pharmacol. 2020 Jun;15(2):326-339. doi: 10.1007/s11481-019-0
o 2018 Jan 25. PMID: 29402799.
o 2018 Jan 25. PMID: 29402799.
o 2018 Jan 25. PMID: 29402799.
o 2018 Jan 25. PMID: 29402799.
o 2018 Jan 25. PMID: 29402799.
7(4):2417-2425. doi: 10.1007/s11033-019-05128-4. Epub 2020 Feb 27. PMID: 32108303.
int.2019.104629. Epub 2019 Dec 6. PMID: 31816340.
int.2019.104629. Epub 2019 Dec 6. PMID: 31816340.
int.2019.104629. Epub 2019 Dec 6. PMID: 31816340.
int.2019.104629. Epub 2019 Dec 6. PMID: 31816340.
021.4885. Epub 2021 Feb 12. PMID: 33576458; PMCID: PMC7895518.
021.4885. Epub 2021 Feb 12. PMID: 33576458; PMCID: PMC7895518.
021.4885. Epub 2021 Feb 12. PMID: 33576458; PMCID: PMC7895518.
021.4885. Epub 2021 Feb 12. PMID: 33576458; PMCID: PMC7895518.
021.4885. Epub 2021 Feb 12. PMID: 33576458; PMCID: PMC7895518.
021.4885. Epub 2021 Feb 12. PMID: 33576458; PMCID: PMC7895518.
)-7212. doi: 10.1007/s12035-015-9627-y. Epub 2015 Dec 19. Erratum in: Mol Neurobiol. 2017 Apr;5
)-7212. doi: 10.1007/s12035-015-9627-y. Epub 2015 Dec 19. Erratum in: Mol Neurobiol. 2017 Apr;5
acol. 2012 Jan 15;674(2-3):132-42. doi: 10.1016/j.ejphar.2011.11.014. Epub 2011 Nov 19. PMID: 22
acol. 2012 Jan 15;674(2-3):132-42. doi: 10.1016/j.ejphar.2011.11.014. Epub 2011 Nov 19. PMID: 22

acol. 2012 Jan 15;674(2-3):132-42. doi: 10.1016/j.ejphar.2011.11.014. Epub 2011 Nov 19. PMID: 22
008. Epub 2017 May 8. PMID: 28495517; PMCID: PMC5812778.
008. Epub 2017 May 8. PMID: 28495517; PMCID: PMC5812778.
008. Epub 2017 May 8. PMID: 28495517; PMCID: PMC5812778.
008. Epub 2017 May 8. PMID: 28495517; PMCID: PMC5812778.
421-7. doi: 10.1016/j.bbrc.2015.05.088. Epub 2015 Jun 1. PMID: 26043690.
421-7. doi: 10.1016/j.bbrc.2015.05.088. Epub 2015 Jun 1. PMID: 26043690.
421-7. doi: 10.1016/j.bbrc.2015.05.088. Epub 2015 Jun 1. PMID: 26043690.
X414533. PMID: 20501058.
X414533. PMID: 20501058.
7119851260. Epub 2019 Jun 28. PMID: 31250662.
7119851260. Epub 2019 Jun 28. PMID: 31250662.
7119851260. Epub 2019 Jun 28. PMID: 31250662.
7119851260. Epub 2019 Jun 28. PMID: 31250662.
7119851260. Epub 2019 Jun 28. PMID: 31250662.
.142/S0192415X10007865. PMID: 20387227.
.142/S0192415X10007865. PMID: 20387227.
.142/S0192415X10007865. PMID: 20387227.
.142/S0192415X10007865. PMID: 20387227.
5(3):391-399. doi: 10.1007/s12031-018-1063-9. Epub 2018 Jul 4. PMID: 29974381.
5(3):391-399. doi: 10.1007/s12031-018-1063-9. Epub 2018 Jul 4. PMID: 29974381.
5(3):391-399. doi: 10.1007/s12031-018-1063-9. Epub 2018 Jul 4. PMID: 29974381.
5(3):391-399. doi: 10.1007/s12031-018-1063-9. Epub 2018 Jul 4. PMID: 29974381.
d reperfusion: Involvement of Src kinase. Sci Rep. 2015 Jun 10;5:11155. doi: 10.1038/srep11155. P
d reperfusion: Involvement of Src kinase. Sci Rep. 2015 Jun 10;5:11155. doi: 10.1038/srep11155. P
d reperfusion: Involvement of Src kinase. Sci Rep. 2015 Jun 10;5:11155. doi: 10.1038/srep11155. P
):400-415. doi: 10.1111/bph.14537. Epub 2018 Dec 18. PMID: 30414381; PMCID: PMC6329622.
):129-139. doi: 10.1007/s12031-017-1006-x. Epub 2017 Dec 15. PMID: 29243061.
):129-139. doi: 10.1007/s12031-017-1006-x. Epub 2017 Dec 15. PMID: 29243061.
):129-139. doi: 10.1007/s12031-017-1006-x. Epub 2017 Dec 15. PMID: 29243061.
):129-139. doi: 10.1007/s12031-017-1006-x. Epub 2017 Dec 15. PMID: 29243061.
):129-139. doi: 10.1007/s12031-017-1006-x. Epub 2017 Dec 15. PMID: 29243061.

hway. Biochem Biophys Res Commun. 2018 Feb 5;496(2):515-522. doi: 10.1016/j.bbrc.2018.01.089
hway. Biochem Biophys Res Commun. 2018 Feb 5;496(2):515-522. doi: 10.1016/j.bbrc.2018.01.089
hway. Biochem Biophys Res Commun. 2018 Feb 5;496(2):515-522. doi: 10.1016/j.bbrc.2018.01.089
72; PMCID: PMC5822585.
72; PMCID: PMC5822585.
72; PMCID: PMC5822585.
72; PMCID: PMC5822585.
2019 Apr 9;13:1135-1144. doi: 10.2147/DDDT.S194182. PMID: 31040648; PMCID: PMC6461000.
2019 Apr 9;13:1135-1144. doi: 10.2147/DDDT.S194182. PMID: 31040648; PMCID: PMC6461000.

Jul;184:1-6. doi: 10.1016/j.jphotobiol.2018.05.010. Epub 2018 May 7. PMID: 29777940.
oi: 10.1007/s11064-014-1315-1. Epub 2014 Apr 29. PMID: 24777807.
oi: 10.1007/s11064-014-1315-1. Epub 2014 Apr 29. PMID: 24777807.
oi: 10.1007/s11064-014-1315-1. Epub 2014 Apr 29. PMID: 24777807.
oi: 10.1007/s11064-014-1315-1. Epub 2014 Apr 29. PMID: 24777807.
doi: 10.1016/j.neuint.2012.06.009. Epub 2012 Jun 16. PMID: 22709670.
doi: 10.1016/j.neuint.2012.06.009. Epub 2012 Jun 16. PMID: 22709670.
doi: 10.1016/j.neuint.2012.06.009. Epub 2012 Jun 16. PMID: 22709670.
doi: 10.1016/j.neuint.2012.06.009. Epub 2012 Jun 16. PMID: 22709670.
doi: 10.1016/j.neuint.2012.06.009. Epub 2012 Jun 16. PMID: 22709670.
Neurosci. 2015 Apr;55(4):872-9. doi: 10.1007/s12031-014-0441-1. Epub 2014 Oct 21. PMID: 253308
Neurosci. 2015 Apr;55(4):872-9. doi: 10.1007/s12031-014-0441-1. Epub 2014 Oct 21. PMID: 253308
Neurosci. 2015 Apr;55(4):872-9. doi: 10.1007/s12031-014-0441-1. Epub 2014 Oct 21. PMID: 253308
col. 2017 Sep;50:208-215. doi: 10.1016/j.intimp.2017.06.029. Epub 2017 Jul 3. PMID: 28683365.
col. 2017 Sep;50:208-215. doi: 10.1016/j.intimp.2017.06.029. Epub 2017 Jul 3. PMID: 28683365.
col. 2017 Sep;50:208-215. doi: 10.1016/j.intimp.2017.06.029. Epub 2017 Jul 3. PMID: 28683365.
col. 2017 Sep;50:208-215. doi: 10.1016/j.intimp.2017.06.029. Epub 2017 Jul 3. PMID: 28683365.
col. 2017 Sep;50:208-215. doi: 10.1016/j.intimp.2017.06.029. Epub 2017 Jul 3. PMID: 28683365.
hav. 2014 Jul;122:7-15. doi: 10.1016/j.pbb.2014.03.004. Epub 2014 Mar 12. PMID: 24631487.
hav. 2014 Jul;122:7-15. doi: 10.1016/j.pbb.2014.03.004. Epub 2014 Mar 12. PMID: 24631487.
hav. 2014 Jul;122:7-15. doi: 10.1016/j.pbb.2014.03.004. Epub 2014 Mar 12. PMID: 24631487.
hav. 2014 Jul;122:7-15. doi: 10.1016/j.pbb.2014.03.004. Epub 2014 Mar 12. PMID: 24631487.
hav. 2014 Jul;122:7-15. doi: 10.1016/j.pbb.2014.03.004. Epub 2014 Mar 12. PMID: 24631487.
hav. 2014 Jul;122:7-15. doi: 10.1016/j.pbb.2014.03.004. Epub 2014 Mar 12. PMID: 24631487.

018-1081-0. PMID: 29426336; PMCID: PMC5807735.
018-1081-0. PMID: 29426336; PMCID: PMC5807735.
018-1081-0. PMID: 29426336; PMCID: PMC5807735.
018-1081-0. PMID: 29426336; PMCID: PMC5807735.
018-1081-0. PMID: 29426336; PMCID: PMC5807735.
018-1081-0. PMID: 29426336; PMCID: PMC5807735.
018-1081-0. PMID: 29426336; PMCID: PMC5807735.
018-1081-0. PMID: 29426336; PMCID: PMC5807735.
018-1081-0. PMID: 29426336; PMCID: PMC5807735.
018-1081-0. PMID: 29426336; PMCID: PMC5807735.
018-1081-0. PMID: 29426336; PMCID: PMC5807735.
018-1081-0. PMID: 29426336; PMCID: PMC5807735.
njury by inhibiting the TLR4/NF-κB signaling pathway. Ann Transl Med. 2021 Nov;9(22):1694. doi: 10
njury by inhibiting the TLR4/NF-κB signaling pathway. Ann Transl Med. 2021 Nov;9(22):1694. doi: 10
njury by inhibiting the TLR4/NF-κB signaling pathway. Ann Transl Med. 2021 Nov;9(22):1694. doi: 10
njury by inhibiting the TLR4/NF-κB signaling pathway. Ann Transl Med. 2021 Nov;9(22):1694. doi: 10
ither than Complement C3 Activity. Inflammation. 2019 Oct;42(5):1830-1842. doi: 10.1007/s10753-
ither than Complement C3 Activity. Inflammation. 2019 Oct;42(5):1830-1842. doi: 10.1007/s10753-
ither than Complement C3 Activity. Inflammation. 2019 Oct;42(5):1830-1842. doi: 10.1007/s10753-

ather than Complement C3 Activity. Inflammation. 2019 Oct;42(5):1830-1842. doi: 10.1007/s10753-
macol. 2020 Jul;84:106547. doi: 10.1016/j.intimp.2020.106547. Epub 2020 Apr 30. PMID: 3236165
macol. 2020 Jul;84:106547. doi: 10.1016/j.intimp.2020.106547. Epub 2020 Apr 30. PMID: 3236165
macol. 2020 Jul;84:106547. doi: 10.1016/j.intimp.2020.106547. Epub 2020 Apr 30. PMID: 3236165
macol. 2020 Jul;84:106547. doi: 10.1016/j.intimp.2020.106547. Epub 2020 Apr 30. PMID: 3236165
macol. 2020 Jul;84:106547. doi: 10.1016/j.intimp.2020.106547. Epub 2020 Apr 30. PMID: 3236165
-716. doi: 10.1159/000480657. Epub 2017 Sep 22. PMID: 28957809.

p.12261. Epub 2014 Apr 10. PMID: 24720795.
p.12261. Epub 2014 Apr 10. PMID: 24720795.
p.12261. Epub 2014 Apr 10. PMID: 24720795.

000101.
pub 2015 Aug 4. PMID: 26240040.
pub 2015 Aug 4. PMID: 26240040.
pub 2015 Aug 4. PMID: 26240040.
pub 2015 Aug 4. PMID: 26240040.
pub 2015 Aug 4. PMID: 26240040.
pub 2015 Aug 4. PMID: 26240040.
pub 2015 Aug 4. PMID: 26240040.
pub 2015 Aug 4. PMID: 26240040.
pub 2015 Aug 4. PMID: 26240040.
pub 2015 Aug 4. PMID: 26240040.
2):267-278. doi: 10.1007/s12031-016-0870-0. Epub 2017 Jan 4. PMID: 28054226.
2):267-278. doi: 10.1007/s12031-016-0870-0. Epub 2017 Jan 4. PMID: 28054226.
2):267-278. doi: 10.1007/s12031-016-0870-0. Epub 2017 Jan 4. PMID: 28054226.
2):267-278. doi: 10.1007/s12031-016-0870-0. Epub 2017 Jan 4. PMID: 28054226.
PMID: 23646480.
PMID: 23646480.
PMID: 23646480.
s]. Zhongguo Zhong Xi Yi Jie He Za Zhi. 2013 Dec;33(12):1696-700. Chinese. PMID: 24517072.
MID: 31412899; PMCID: PMC6694654.
MID: 31412899; PMCID: PMC6694654.
MID: 31412899; PMCID: PMC6694654.
n Int. 2012 Apr;60(5):458-65. doi: 10.1016/j.neuint.2012.01.026. Epub 2012 Feb 8. PMID: 2234282
n Int. 2012 Apr;60(5):458-65. doi: 10.1016/j.neuint.2012.01.026. Epub 2012 Feb 8. PMID: 2234282
-119. doi: 10.1016/j.neulet.2016.12.046. Epub 2016 Dec 21. PMID: 28011393.
J Ethnopharmacol. 2020 Feb 10;248:112319. doi: 10.1016/j.jep.2019.112319. Epub 2019 Oct 19. PM
J Ethnopharmacol. 2020 Feb 10;248:112319. doi: 10.1016/j.jep.2019.112319. Epub 2019 Oct 19. PM
J Ethnopharmacol. 2020 Feb 10;248:112319. doi: 10.1016/j.jep.2019.112319. Epub 2019 Oct 19. PM
J Ethnopharmacol. 2020 Feb 10;248:112319. doi: 10.1016/j.jep.2019.112319. Epub 2019 Oct 19. PM
J Ethnopharmacol. 2020 Feb 10;248:112319. doi: 10.1016/j.jep.2019.112319. Epub 2019 Oct 19. PM
J Ethnopharmacol. 2020 Feb 10;248:112319. doi: 10.1016/j.jep.2019.112319. Epub 2019 Oct 19. PM
J Ethnopharmacol. 2020 Feb 10;248:112319. doi: 10.1016/j.jep.2019.112319. Epub 2019 Oct 19. PM

J Ethnopharmacol. 2020 Feb 10;248:112319. doi: 10.1016/j.jep.2019.112319. Epub 2019 Oct 19. PM
J Ethnopharmacol. 2020 Feb 10;248:112319. doi: 10.1016/j.jep.2019.112319. Epub 2019 Oct 19. PM
);2021:9925561. doi: 10.1155/2021/9925561. PMID: 35003524; PMCID: PMC8739174.
);2021:9925561. doi: 10.1155/2021/9925561. PMID: 35003524; PMCID: PMC8739174.
);2021:9925561. doi: 10.1155/2021/9925561. PMID: 35003524; PMCID: PMC8739174.
);2021:9925561. doi: 10.1155/2021/9925561. PMID: 35003524; PMCID: PMC8739174.
);2021:9925561. doi: 10.1155/2021/9925561. PMID: 35003524; PMCID: PMC8739174.
);2021:9925561. doi: 10.1155/2021/9925561. PMID: 35003524; PMCID: PMC8739174.
mac.2013.10.034. Epub 2013 Nov 1. PMID: 24189392.
mac.2013.10.034. Epub 2013 Nov 1. PMID: 24189392.
mac.2013.10.034. Epub 2013 Nov 1. PMID: 24189392.
mac.2013.10.034. Epub 2013 Nov 1. PMID: 24189392.
11 Oct;77(15):1669-79. doi: 10.1055/s-0030-1271019. Epub 2011 Apr 21. PMID: 21512969.
11 Oct;77(15):1669-79. doi: 10.1055/s-0030-1271019. Epub 2011 Apr 21. PMID: 21512969.
tion of cerebral endothelial cell apoptosis. Pharm Biol. 2013 Sep;51(9):1150-7. doi: 10.3109/138802
ygenase 1 signaling in primary cerebral endothelial cells for definite protection against ischemic stro
PMID: 34368862; PMCID: PMC8416143.
PMID: 34368862; PMCID: PMC8416143.
PMID: 34368862; PMCID: PMC8416143.
PMID: 34368862; PMCID: PMC8416143.
PMID: 34368862; PMCID: PMC8416143.

o. Cell Stress Chaperones. 2019 Mar;24(2):441-452. doi: 10.1007/s12192-019-00977-1. Epub 2019 F
o. Cell Stress Chaperones. 2019 Mar;24(2):441-452. doi: 10.1007/s12192-019-00977-1. Epub 2019 F
o. Cell Stress Chaperones. 2019 Mar;24(2):441-452. doi: 10.1007/s12192-019-00977-1. Epub 2019 F
o. Cell Stress Chaperones. 2019 Mar;24(2):441-452. doi: 10.1007/s12192-019-00977-1. Epub 2019 F
o. Cell Stress Chaperones. 2019 Mar;24(2):441-452. doi: 10.1007/s12192-019-00977-1. Epub 2019 F
o. Cell Stress Chaperones. 2019 Mar;24(2):441-452. doi: 10.1007/s12192-019-00977-1. Epub 2019 F
o. Cell Stress Chaperones. 2019 Mar;24(2):441-452. doi: 10.1007/s12192-019-00977-1. Epub 2019 F
o. Cell Stress Chaperones. 2019 Mar;24(2):441-452. doi: 10.1007/s12192-019-00977-1. Epub 2019 F
a Pharmacol Sin. 2018 Aug;39(8):1259-1272. doi: 10.1038/aps.2017.149. Epub 2018 Mar 15. PMID:
a Pharmacol Sin. 2018 Aug;39(8):1259-1272. doi: 10.1038/aps.2017.149. Epub 2018 Mar 15. PMID:
a Pharmacol Sin. 2018 Aug;39(8):1259-1272. doi: 10.1038/aps.2017.149. Epub 2018 Mar 15. PMID:
J Ethnopharmacol. 2020 Mar 1;249:112365. doi: 10.1016/j.jep.2019.112365. Epub 2019 Oct 31. PM
J Ethnopharmacol. 2020 Mar 1;249:112365. doi: 10.1016/j.jep.2019.112365. Epub 2019 Oct 31. PM
J Ethnopharmacol. 2020 Mar 1;249:112365. doi: 10.1016/j.jep.2019.112365. Epub 2019 Oct 31. PM
J Ethnopharmacol. 2020 Mar 1;249:112365. doi: 10.1016/j.jep.2019.112365. Epub 2019 Oct 31. PM
30(9):105987. doi: 10.1016/j.jstrokecerebrovasdis.2021.105987. Epub 2021 Jul 14. PMID: 3427370
30(9):105987. doi: 10.1016/j.jstrokecerebrovasdis.2021.105987. Epub 2021 Jul 14. PMID: 3427370
30(9):105987. doi: 10.1016/j.jstrokecerebrovasdis.2021.105987. Epub 2021 Jul 14. PMID: 3427370
cription factor κappaB signaling pathway. Neuroscience. 2011 Mar 10;176:408-19. doi: 10.1016/j.n
cription factor κappaB signaling pathway. Neuroscience. 2011 Mar 10;176:408-19. doi: 10.1016/j.n
cription factor κappaB signaling pathway. Neuroscience. 2011 Mar 10;176:408-19. doi: 10.1016/j.n

cription factor kappaB signaling pathway. Neuroscience. 2011 Mar 10;176:408-19. doi: 10.1016/j.n
Jun;102:874-883. doi: 10.1016/j.biopha.2018.03.087. Epub 2018 Apr 5. PMID: 29728011.
Jun;102:874-883. doi: 10.1016/j.biopha.2018.03.087. Epub 2018 Apr 5. PMID: 29728011.
Jun;102:874-883. doi: 10.1016/j.biopha.2018.03.087. Epub 2018 Apr 5. PMID: 29728011.
Jun;102:874-883. doi: 10.1016/j.biopha.2018.03.087. Epub 2018 Apr 5. PMID: 29728011.
Jun;102:874-883. doi: 10.1016/j.biopha.2018.03.087. Epub 2018 Apr 5. PMID: 29728011.
Jun;102:874-883. doi: 10.1016/j.biopha.2018.03.087. Epub 2018 Apr 5. PMID: 29728011.
Jun;102:874-883. doi: 10.1016/j.biopha.2018.03.087. Epub 2018 Apr 5. PMID: 29728011.
l. 2021 Mar 4;73(2):272-280. doi: 10.1093/jpp/rgaa028. PMID: 33793797.
l. 2021 Mar 4;73(2):272-280. doi: 10.1093/jpp/rgaa028. PMID: 33793797.
l. 2021 Mar 4;73(2):272-280. doi: 10.1093/jpp/rgaa028. PMID: 33793797.
l. 2021 Mar 4;73(2):272-280. doi: 10.1093/jpp/rgaa028. PMID: 33793797.
l. 2021 Mar 4;73(2):272-280. doi: 10.1093/jpp/rgaa028. PMID: 33793797.
l. 2021 Mar 4;73(2):272-280. doi: 10.1093/jpp/rgaa028. PMID: 33793797.
l. 2021 Mar 4;73(2):272-280. doi: 10.1093/jpp/rgaa028. PMID: 33793797.
l. 2021 Mar 4;73(2):272-280. doi: 10.1093/jpp/rgaa028. PMID: 33793797.
485. doi: 10.3389/fphar.2021.772485. PMID: 35095491; PMCID: PMC8795828.
485. doi: 10.3389/fphar.2021.772485. PMID: 35095491; PMCID: PMC8795828.
485. doi: 10.3389/fphar.2021.772485. PMID: 35095491; PMCID: PMC8795828.
485. doi: 10.3389/fphar.2021.772485. PMID: 35095491; PMCID: PMC8795828.
88:13-20. doi: 10.1016/j.jep.2016.04.058. Epub 2016 May 2. PMID: 27151150.
88:13-20. doi: 10.1016/j.jep.2016.04.058. Epub 2016 May 2. PMID: 27151150.
88:13-20. doi: 10.1016/j.jep.2016.04.058. Epub 2016 May 2. PMID: 27151150.
20 Apr 21;21(8):2899. doi: 10.3390/ijms21082899. PMID: 32326191; PMCID: PMC7215870.
20 Apr 21;21(8):2899. doi: 10.3390/ijms21082899. PMID: 32326191; PMCID: PMC7215870.
20 Apr 21;21(8):2899. doi: 10.3390/ijms21082899. PMID: 32326191; PMCID: PMC7215870.
20 Apr 21;21(8):2899. doi: 10.3390/ijms21082899. PMID: 32326191; PMCID: PMC7215870.
.052. Epub 2011 May 1. PMID: 21554870.
.052. Epub 2011 May 1. PMID: 21554870.
.052. Epub 2011 May 1. PMID: 21554870.
.052. Epub 2011 May 1. PMID: 21554870.
.052. Epub 2011 May 1. PMID: 21554870.
j.ejphar.2019.04.015. Epub 2019 Apr 10. PMID: 30978320.
uroinflammation. 2021 Jun 15;18(1):137. doi: 10.1186/s12974-021-02188-x. PMID: 34130727; PMC
uroinflammation. 2021 Jun 15;18(1):137. doi: 10.1186/s12974-021-02188-x. PMID: 34130727; PMC
uroinflammation. 2021 Jun 15;18(1):137. doi: 10.1186/s12974-021-02188-x. PMID: 34130727; PMC
uroinflammation. 2021 Jun 15;18(1):137. doi: 10.1186/s12974-021-02188-x. PMID: 34130727; PMC
uroinflammation. 2021 Jun 15;18(1):137. doi: 10.1186/s12974-021-02188-x. PMID: 34130727; PMC
uroinflammation. 2021 Jun 15;18(1):137. doi: 10.1186/s12974-021-02188-x. PMID: 34130727; PMC
3. PMID: 27871155; PMCID: PMC5424637.
3. PMID: 27871155; PMCID: PMC5424637.
3. PMID: 27871155; PMCID: PMC5424637.
3. PMID: 27871155; PMCID: PMC5424637.
3. PMID: 27871155; PMCID: PMC5424637.
3. PMID: 27871155; PMCID: PMC5424637.
3. PMID: 27871155; PMCID: PMC5424637.

3. PMID: 27871155; PMCID: PMC5424637.
3. PMID: 27871155; PMCID: PMC5424637.
3. PMID: 27871155; PMCID: PMC5424637.
l Death Following Transient Global Cerebral Ischemia in High-Fat Diet-Induced Obese Gerbils. Int J N
l Death Following Transient Global Cerebral Ischemia in High-Fat Diet-Induced Obese Gerbils. Int J N
l Death Following Transient Global Cerebral Ischemia in High-Fat Diet-Induced Obese Gerbils. Int J N
l Death Following Transient Global Cerebral Ischemia in High-Fat Diet-Induced Obese Gerbils. Int J N
l Death Following Transient Global Cerebral Ischemia in High-Fat Diet-Induced Obese Gerbils. Int J N
hippocampal CA1 area via reducing of glial cell activation and oxidative stress. Biomed Pharmacothe
hippocampal CA1 area via reducing of glial cell activation and oxidative stress. Biomed Pharmacothe
hippocampal CA1 area via reducing of glial cell activation and oxidative stress. Biomed Pharmacothe
hippocampal CA1 area via reducing of glial cell activation and oxidative stress. Biomed Pharmacothe
hippocampal CA1 area via reducing of glial cell activation and oxidative stress. Biomed Pharmacothe
bal Synaptic Neuroplasticity. Mol Neurobiol. 2021 Oct;58(10):5338-5355. doi: 10.1007/s12035-021-(
bal Synaptic Neuroplasticity. Mol Neurobiol. 2021 Oct;58(10):5338-5355. doi: 10.1007/s12035-021-(
oral function. Neuropharmacology. 2012 Oct;63(5):776-83. doi: 10.1016/j.neuropharm.2012.05.034
ery occlusion in mice. Neurotox Res. 2014 Nov;26(4):307-16. doi: 10.1007/s12640-014-9457-0. Epub
ol Biol Psychiatry. 2017 Apr 3;75:94-105. doi: 10.1016/j.pnpbp.2016.11.005. Epub 2016 Nov 23. PM
ol Biol Psychiatry. 2017 Apr 3;75:94-105. doi: 10.1016/j.pnpbp.2016.11.005. Epub 2016 Nov 23. PM
ol Biol Psychiatry. 2017 Apr 3;75:94-105. doi: 10.1016/j.pnpbp.2016.11.005. Epub 2016 Nov 23. PM
ol Biol Psychiatry. 2017 Apr 3;75:94-105. doi: 10.1016/j.pnpbp.2016.11.005. Epub 2016 Nov 23. PM
ation Through Attenuation of iNOS, COX-2 Expression and NFκ-B Signaling Pathway. Neuromolecula
ation Through Attenuation of iNOS, COX-2 Expression and NFκ-B Signaling Pathway. Neuromolecula
ation Through Attenuation of iNOS, COX-2 Expression and NFκ-B Signaling Pathway. Neuromolecula
ation Through Attenuation of iNOS, COX-2 Expression and NFκ-B Signaling Pathway. Neuromolecula
ation Through Attenuation of iNOS, COX-2 Expression and NFκ-B Signaling Pathway. Neuromolecula
ation Through Attenuation of iNOS, COX-2 Expression and NFκ-B Signaling Pathway. Neuromolecula
ation Through Attenuation of iNOS, COX-2 Expression and NFκ-B Signaling Pathway. Neuromolecula
ation Through Attenuation of iNOS, COX-2 Expression and NFκ-B Signaling Pathway. Neuromolecula
ation Through Attenuation of iNOS, COX-2 Expression and NFκ-B Signaling Pathway. Neuromolecula
ation Through Attenuation of iNOS, COX-2 Expression and NFκ-B Signaling Pathway. Neuromolecula

41106.
41106.
41106.
41106.
41106.
41106.
41106.
41106.
fphar.2021.599543. PMID: 34234667; PMCID: PMC8255628.
fphar.2021.599543. PMID: 34234667; PMCID: PMC8255628.
fphar.2021.599543. PMID: 34234667; PMCID: PMC8255628.
MID: 26485504.
MID: 26485504.
MID: 26485504.
MID: 26485504.
ID: 31228802.
ID: 31228802.
ID: 31228802.
ID: 31228802.
ID: 31228802.
ID: 31228802.
ID: 31228802.
ID: 31228802.
ID: 31228802.

2017-019-08582-2. Epub 2019 Dec 2. PMID: 31792810.
2017-019-08582-2. Epub 2019 Dec 2. PMID: 31792810.
2017-019-08582-2. Epub 2019 Dec 2. PMID: 31792810.
2017-019-08582-2. Epub 2019 Dec 2. PMID: 31792810.
2017-019-08582-2. Epub 2019 Dec 2. PMID: 31792810.

)9903-9. Epub 2020 Jan 11. PMID: 31927682.
)9903-9. Epub 2020 Jan 11. PMID: 31927682.

4(3):2373-2374. PMID: 26687185.
4(3):2373-2374. PMID: 26687185.
119079.
119079.

119079.

MID: 26059793; PMCID: PMC4461916.
MID: 26059793; PMCID: PMC4461916.
MID: 26059793; PMCID: PMC4461916.
. Epub 2018 Jan 17. PMID: 29353037.
. Epub 2018 Jan 17. PMID: 29353037.
. Epub 2018 Jan 17. PMID: 29353037.

360.
360.
360.

0.21037/atm-21-5752. PMID: 34988203; PMCID: PMC8667139
0.21037/atm-21-5752. PMID: 34988203; PMCID: PMC8667140
0.21037/atm-21-5752. PMID: 34988203; PMCID: PMC8667140
0.21037/atm-21-5752. PMID: 34988203; PMCID: PMC8667140
019-01045-7. PMID: 31230155.
019-01045-7. PMID: 31230155.
019-01045-7. PMID: 31230155.

019-01045-7. PMID: 31230155.
2.
2.
2.
2.
2.

3.
3.

MID: 31639488.
MID: 31639488.
MID: 31639488.
MID: 31639488.
MID: 31639488.
MID: 31639488.
MID: 31639488.

MID: 31639488.
MID: 31639488.

209.2013.782051. Epub 2013 Jun 12. PMID: 23930775.
oke in rats. Transl Res. 2016 Apr;170:57-72. doi: 10.1016/j.trsl.2015.12.002. Epub 2015 Dec 17. PMID: 26746

Feb 27. PMID: 30815818; PMCID: PMC6439064.
Feb 27. PMID: 30815818; PMCID: PMC6439064.
Feb 27. PMID: 30815818; PMCID: PMC6439064.
Feb 27. PMID: 30815818; PMCID: PMC6439064.
Feb 27. PMID: 30815818; PMCID: PMC6439064.
Feb 27. PMID: 30815818; PMCID: PMC6439064.
Feb 27. PMID: 30815818; PMCID: PMC6439064.
Feb 27. PMID: 30815818; PMCID: PMC6439064.
29542683; PMCID: PMC6289361.
29542683; PMCID: PMC6289361.
29542683; PMCID: PMC6289361.
MID: 31678414.
MID: 31678414.
MID: 31678414.
MID: 31678414.
3.
3.
3.
euroscience.2010.11.029. Epub 2010 Dec 16. PMID: 21168474.
euroscience.2010.11.029. Epub 2010 Dec 16. PMID: 21168474.
euroscience.2010.11.029. Epub 2010 Dec 16. PMID: 21168474.

euroscience.2010.11.029. Epub 2010 Dec 16. PMID: 21168474.

ID: PMC8207641.
ID: PMC8207641.
ID: PMC8207641.
ID: PMC8207641.
ID: PMC8207641.
ID: PMC8207641.

Mol Sci. 2019 Jan 28;20(3):554. doi: 10.3390/ijms20030554. PMID: 30696078; PMCID: PMC6387260.
Mol Sci. 2019 Jan 28;20(3):554. doi: 10.3390/ijms20030554. PMID: 30696078; PMCID: PMC6387260.
Mol Sci. 2019 Jan 28;20(3):554. doi: 10.3390/ijms20030554. PMID: 30696078; PMCID: PMC6387260.
Mol Sci. 2019 Jan 28;20(3):554. doi: 10.3390/ijms20030554. PMID: 30696078; PMCID: PMC6387260.
Mol Sci. 2019 Jan 28;20(3):554. doi: 10.3390/ijms20030554. PMID: 30696078; PMCID: PMC6387260.
r. 2019 Jan;109:1718-1727. doi: 10.1016/j.biopha.2018.11.015. Epub 2018 Nov 20. PMID: 30551426.
r. 2019 Jan;109:1718-1727. doi: 10.1016/j.biopha.2018.11.015. Epub 2018 Nov 20. PMID: 30551426.
r. 2019 Jan;109:1718-1727. doi: 10.1016/j.biopha.2018.11.015. Epub 2018 Nov 20. PMID: 30551426.
r. 2019 Jan;109:1718-1727. doi: 10.1016/j.biopha.2018.11.015. Epub 2018 Nov 20. PMID: 30551426.
r. 2019 Jan;109:1718-1727. doi: 10.1016/j.biopha.2018.11.015. Epub 2018 Nov 20. PMID: 30551426.
02479-7. Epub 2021 Jul 24. PMID: 34302281.
02479-7. Epub 2021 Jul 24. PMID: 34302281.
. Epub 2012 May 30. PMID: 22659086.
2014 Feb 15. PMID: 24532152.
ID: 27889412.
ID: 27889412.
ID: 27889412.
ID: 27889412.
r Med. 2016 Sep;18(3):322-33. doi: 10.1007/s12017-016-8395-9. Epub 2016 Apr 18. PMID: 27087648.
r Med. 2016 Sep;18(3):322-33. doi: 10.1007/s12017-016-8395-9. Epub 2016 Apr 18. PMID: 27087648.
r Med. 2016 Sep;18(3):322-33. doi: 10.1007/s12017-016-8395-9. Epub 2016 Apr 18. PMID: 27087648.
r Med. 2016 Sep;18(3):322-33. doi: 10.1007/s12017-016-8395-9. Epub 2016 Apr 18. PMID: 27087648.
r Med. 2016 Sep;18(3):322-33. doi: 10.1007/s12017-016-8395-9. Epub 2016 Apr 18. PMID: 27087648.
r Med. 2016 Sep;18(3):322-33. doi: 10.1007/s12017-016-8395-9. Epub 2016 Apr 18. PMID: 27087648.
r Med. 2016 Sep;18(3):322-33. doi: 10.1007/s12017-016-8395-9. Epub 2016 Apr 18. PMID: 27087648.
r Med. 2016 Sep;18(3):322-33. doi: 10.1007/s12017-016-8395-9. Epub 2016 Apr 18. PMID: 27087648.
r Med. 2016 Sep;18(3):322-33. doi: 10.1007/s12017-016-8395-9. Epub 2016 Apr 18. PMID: 27087648.
r Med. 2016 Sep;18(3):322-33. doi: 10.1007/s12017-016-8395-9. Epub 2016 Apr 18. PMID: 27087648.

302.
